# Exploring Limit Cycles of Differential Equations through Information Geometry Unveils the Solution to Hilbert’s 16th Problem

**DOI:** 10.3390/e26090745

**Published:** 2024-08-30

**Authors:** Vinícius Barros da Silva, João Peres Vieira, Edson Denis Leonel

**Affiliations:** 1 Department of Physics, Universidade Estadual Paulista “Júlio de Mesquita Filho”, Campus de Rio Claro, São Paulo 13506-900, Brazil; edson-denis.leonel@unesp.br; 2 Department of Mathematics, Universidade Estadual Paulista “Júlio de Mesquita Filho”, Campus de Rio Claro, São Paulo 13506-900, Brazil; joao.peres@unesp.br

**Keywords:** differential equations, dynamical systems, limit cycles, Hilbert’s sixteenth problem

## Abstract

The detection of limit cycles of differential equations poses a challenge due to the type of the nonlinear system, the regime of interest, and the broader context of applicable models. Consequently, attempts to solve Hilbert’s sixteenth problem on the maximum number of limit cycles of polynomial differential equations have been uniformly unsuccessful due to failing results and their lack of consistency. Here, the answer to this problem is finally obtained through information geometry, in which the Riemannian metrical structure of the parameter space of differential equations is investigated with the aid of the Fisher information metric and its scalar curvature R. We find that the total number of divergences of |R| to infinity provides the maximum number of limit cycles of differential equations. Additionally, we demonstrate that real polynomial systems of degree n≥2 have the maximum number of 2(n−1)(4(n−1)−2) limit cycles. The research findings highlight the effectiveness of geometric methods in analyzing complex systems and offer valuable insights across information theory, applied mathematics, and nonlinear dynamics. These insights may pave the way for advancements in differential equations, presenting exciting opportunities for future developments.

## 1. Introduction

On 8 August 1900, D. Hilbert presented a magnificent list of problems at the Second International Congress of Mathematicians in Paris. The following elementary, and yet not quite solved, problem is known as the second half of the sixteenth problem of Hilbert [1]:

In connection with this purely algebraic problem, I wish to bring forward a question which, it seems to me, may be attacked by the same method of continuous variations of coefficients, and whose answer is of corresponding value for the topology of families of curves defined by differential equations. This is the question as to the maximum number and position of Poincaré’s boundary cycles (limit cycles) for a differential equation of the first order and degree of the form

$$\frac{dy}{dx}=\frac{Y}{X},$$

where X and Y are rational integral functions of the *n*th degree in *x* and *y*.

In other words, the second part of Hilbert’s question asks for the maximum number and location of limit cycles of planar polynomial systems of degree *n*. A limit cycle is an isolated closed trajectory in the phase space of an autonomous system of ordinary differential equations [2,3,4,5]. The predictions that regard the number and position of limit cycles from the perspective of classical bifurcation theory (CBT) have long been discredited given the nature of the contradictory results of previous investigations. Despite the simplicity of the question, several attempts have been made throughout history to solve it. However, all such efforts have been fruitless, either failing in the avowed purpose or lacking consistency. The failure to answer the sixteenth problem of Hilbert can be ascribed only to an essential deficiency of CBT and standard methods to study limit cycles rather than a faulty application thereof.

In general, there are three main classes of bifurcation of limit cycles: First, multiple-limit bifurcations. Second, separatrix-cycle bifurcations (from homoclinic or heteroclinic trajectories). Finally, Andronov–Hopf bifurcations from a center or a focus in two-dimensional dynamic systems [6,7,8,9,10,11,12].

The first and second classes of limit-cycle bifurcations are more challenging to investigate since there are few techniques and no alternative methods to the Melnikov function. In the scenario of homoclinic and heteroclinic bifurcations, one may only estimate bifurcation points through the Melnikov method. Although that approach is a universal technique, there are still fundamental and technical difficulties in its practical use in global bifurcations [13,14].

Despite extensive developments in the concepts and techniques to study Hopf bifurcations, it is still not a simple task to analytically determine isolated trajectories in the phase portrait of dynamical systems [15,16]. Moreover, the usual methods employed in the study of limit cycles (e.g., normal form theory, the Bendixson–Dulac criterion, and the Poincaré–Hopf theorem) only provide clues about the location of the possible limit cycles in the phase portraits. More specifically, these methods have several drawbacks that impose difficulties and challenges in the investigation of limit cycles in complex systems. We stress some of them here.

First, the computation of explicit equations of the higher-order normal forms of nonlinear dynamical systems, which is particularly important in the study of Hilbert’s sixteenth problem, is an arduous task and not yet uniquely defined [8,16]. Second, Bendixson’s criterion does not guarantee whether or not a dynamical system has a limit cycle, even if the divergence of a plane vector field is zero [16,17]. Third, even when it is well known that there is no limit cycle, there is no method to determine the smooth function of Dulac’s criterion [16,17,18]. Fourth, one cannot apply the Poincaré–Hopf theorem to higher-dimensional systems [19,20]. Furthermore, if a system has more than one equilibrium point, we cannot determine the exact position of a limit cycle or whether it is unique, since the Poincaré–Hopf theorem does not distinguish between the cases of an equilibrium point and a limit cycle [16,19,20,21]. Finally, the Bendixson–Dulac criterion and Poincaré–Hopf theorem provide only clues about the location of the possible limit cycles in the phase portrait of two-dimensional systems.

Thus, the lack of effective methods for the study of limit cycles indicates that a revision of the classical bifurcation theory is necessary. Despite the nature of the contradictory results on the understanding of Hilbert’s sixteenth problem, it should be emphasized that the application of invariant theories along with algebraic and geometric methods for the investigation of dynamical systems have been pointed out to be useful both for the complete solution of Hilbert’s sixteenth challenge and for solving other open problems of qualitative theory of differential equations [22,23,24].

Recently, a new covariant and geometric formulation of the bifurcation theory was developed and applied to local and global bifurcations without introducing fundamentally new concepts [25,26]. In the geometric bifurcation theory (GBT), a Riemannian metrical and invariant structure of the parameter space was introduced and investigated for dynamical systems described in the framework of differential equations. In our previous papers, we have shown that GBT presents a clear improvement over the original CBT in essentially four ways. First, our formulation addresses local and global stability conditions. Second, it involves the essential features of CBT. Third, homoclinic and heteroclinic bifurcations may be investigated without considering approximate methods or numerical simulations. Finally, GBT provides an alternative way to study problems where the conventional nonlinear methods show limitations.

Based on the above, the fundamental question that we may naturally ask now is whether GBT can unambiguously provide the maximum number and position of limit cycles of dynamical systems in the framework of differential equations. Hence, the major purpose of the present paper is to supply an affirmative answer to the second half of the sixteenth problem of Hilbert by employing the geometric methods of GBT.

While there have been various attempts to solve Hilbert’s 16th problem through CBT and other traditional methods, applying GBT to this problem is a novel approach. Previous studies overwhelmingly depended on approximate methods and numerical simulations, which consistently failed to provide comprehensive solutions. Conversely, the GBT framework, with its Riemannian metrical and invariant structure of the parameter space, offers a more robust and theoretically sound approach. This work represents a significant advancement over previous methodologies by addressing the conditions for the existence and the non-existence of limit cycles without the need for approximation or numerical methods.

The present manuscript introduces several new features that distinguish it from previous studies. Firstly, it provides a covariant and geometric formulation of bifurcation theory specifically tailored to studying limit cycles in dynamical systems. Secondly, it demonstrates the application of GBT in investigating limit cycles in nine different examples. Thirdly, it highlights the practical advantages of GBT over conventional nonlinear methods in determining isolated trajectories in the phase space. These advancements not only shed new light on the second half of Hilbert’s sixteenth problem but also provide a possible avenue for solving other complex issues in the qualitative theory of differential equations.

The study of periodic trajectories and limit cycles in dynamical systems is of great importance not only due to its extensions but also in interpreting practical results, which are interesting theoretically and certainly relevant experimentally. Some notable applications of limit cycles involve bifurcations, biology, chemical reactions, and electrical circuits, among others [20]. However, on account of the fact that limit cycles might be difficult to locate and study, the application of new geometrical methods may shed new light on their study, particularly where the ordinary methods provide either little or no solution.

This article is organized as follows. Section 2 is concerned with the relevant theoretical background. Here, we present the formalism of GBT with particular emphasis on the Riemannian scalar curvature. Section 3 is devoted to the application of GBT to the phenomena of limit cycles. In order to construct a suitable definition and a characterization of limit cycles in the framework of GBT, three classes of dynamical systems described by differential equation are investigated: (I) systems with no limit cycle; (II) systems with only one limit cycle; (III) systems with more than one limit cycle. The analysis is carried out with two alternative formulations, one employing the standard methods to detect limit cycles, and the other using GBT. Section 4 focuses on the second half of Hilbert’s sixteenth problem. Finally, Section 5 is devoted to discussion of our results and conclusions.

## 2. General Theory

Here, we present the theoretical background of GBT. Hence, this section is organized as follows. First, we construct the Riemannian metrical structure of the parameter space for autonomous dynamical systems described as differential equations in the framework of Fisher information geometry.

It is demonstrated that a parameter Gaussian probability distribution leads to a Riemannian metric of the parameter space with components of the metric tensor expressed by derivatives of the nonlinear functions that characterize the system under study. Second, we devote ourselves to the scalar curvature R. To avoid a work of excessive length, we present a revised account of the construction of information geometry and the main developments contained in the initial papers [25,26].

We start by introducing the mathematical model of dynamical systems and their respective Gaussian probability distribution. Let **V** be a real planar vector field and its correspondent nonlinear differential equations,

(1) $$\left\{\begin{array}{ccc} & \beta_{1} & =\frac{ds_{1}}{d\tau }=\Phi_{1}\left(s_{1},s_{2}\right),\\ & \beta_{2} & =\frac{ds_{2}}{d\tau }=\Phi_{2}\left(s_{1},s_{2}\right), \end{array}\right.$$

represent the mathematical model of autonomous dynamical systems in which we employ the following notation: Greek subscripts assume values ranging from 1 to 2. The momenta are expressed by $\beta_{1}$ and $\beta_{2}$. Here, $s_{1}$ and $s_{2}$ denote the order parameters, while τ is time. Furthermore, $\Phi_{1}$ and $\Phi_{2}$ denote the nonlinear functions of the dynamical system, Equation (1), where it is understood that $\Phi_{i}$ may show dependence on one or more control parameters. Finally, we shall consider an equilibrium point of the two-dimensional vector field V to be a point $p_{i}\left(s_{i}^{*}\right)$ such that $V(p_{i})=0$, or equivalently, a point in which $\Phi_{i}\left(p_{i}\right)=0$. It should be remarked that the points in which $\Phi_{i}\left(s_{1},s_{2}\right)=0$ are also known as singular points, critical points, equilibrium points, or equilibrium states of the order parameters [25,26,27].

Also, it should be remarked that a singular point *p* is isolated if V does not vanish at points other than *p* in a sufficiently small neighborhood of *p*. Finally, a non-degenerate singular point of V is always isolated if the determinant of its Jacobian matrix at $p_{i}$ is not zero. That is,

(2) $$\det\;J=|\frac{\partial \left(\Phi_{1},\Phi_{2}\right)}{\partial (s_{1},s_{2})}\left(p_{i}\right)|\neq 0.$$

As we have discussed previously in Ref. [25], the above differential equations, Equation (1), naturally arise from Hamiltonian equations. In addition, the present model is doubtlessly advantageous, as it describes the general form of most of the systems studied in nonlinear dynamics [18,25,26,27,28,29].

With the aid of Fourier’s characteristic functions, the nonlinear dynamical system Equation (1) immediately leads to the following generalized Gaussian probability density [25]:

(3) $$\rho \left(\beta_{1},\beta_{2}\right)=\rho \left(\beta \right)=\left(\frac{T}{\pi }\right)\prod_{i=1}^{2}\exp\left[-T{\left(\beta_{i}-\Phi_{i}\right)}^{2}\right],$$

in which the limit T→∞ is understood. The derivation of the probability density, Equation (3), follows the exact same procedure used to derive Equations (38)–(41) in Ref. [25]. It should be noted that Equation (3) is only valid when *T* is large. According to our discussions on the initial paper [25], the limit T→∞ is essential to make the Gaussian probability density, Equation (3), both physical and mathematical meaningful. That is to say, one must interpret the period of time *T* as a limiting case to what is expected to be a correct approximation to the solutions of Equation (1) at long but finite times. With the knowledge of Equation (3), we can now construct the Riemannian structure into the parameter space of the dynamical system under study. Let us consider Ω a family of probability distributions that is parametrized by two real-order parameters, in which [25,30,31,32,33]

(4) $$\Omega =\left\{P_{X}=\frac{\rho (\beta ;X)}{T};T\in R^{+};X\in R^{2}\right\},$$

whence Ω carries the structure of a Riemannian manifold M of variables $\beta_{1}$ and $\beta_{2}$. In this Gaussian statistical model, $X=\left(X^{1},X^{2}\right)=\left(s_{1},s_{2}\right)$ designates the role of coordinates of a point $P_{X}\in \Omega$. The matrix $G_{\alpha \;\mu }\left(X\right)$ is a covariant symmetric tensor of the second order. This led us to the Fisher information metric,

(5) $$d\ell^{2}=G_{11}{\left(ds_{1}\right)}^{2}+G_{22}{\left(ds_{2}\right)}^{2},$$

which is a positive definite and geometrically invariant Riemannian metric. Following the procedures and techniques developed in section III of Ref. [25], this yields the following metric elements

(6) $$G_{11}=2\left[{\left(\frac{\partial \Phi_{1}}{\partial s_{1}}\right)}^{2}+{\left(\frac{\partial \Phi_{2}}{\partial s_{1}}\right)}^{2}\right],$$

(7) $$G_{22}=2\left[{\left(\frac{\partial \Phi_{1}}{\partial s_{2}}\right)}^{2}+{\left(\frac{\partial \Phi_{2}}{\partial s_{2}}\right)}^{2}\right].$$

The derivation of Equations (6) and (7) follows the same well-developed treatment of Ref. [25]. In addition, here, and henceforth, we confine ourselves to the summation convention. That is, if a letter figures twice in a product, once as subscript and once as superscript, then we omit the summation sign. Please, see Ref. [34] for more details.

Having obtained the information metric, which describes a proper distance in the parameter space *X* of the nonlinear system of Equation (1), we now turn to the calculation of the Ricci scalar curvature. The metric above defines the fourth-rank Riemannian curvature tensor $R_{\xi \;\eta \;l}^{\alpha }$ in terms of the derivatives of the nonlinear functions $\Phi_{1}$ and $\Phi_{2}$. The contraction of $R_{\xi \;\eta \;l}^{\alpha }$ yields the scalar curvature R.

Consequently, we may write that the Riemannian metric, Equation (5), induces the following curvature R on the two-dimensional manifold of the parameter space *X*:

(8) $$R=\frac{1}{\sqrt{G}}\left[\frac{\partial }{\partial s_{1}}\left(\frac{1}{\sqrt{G}}\frac{\partial G_{22}}{\partial s_{1}}\right)+\frac{\partial }{\partial s_{2}}\left(\frac{1}{\sqrt{G}}\frac{\partial G_{11}}{\partial s_{2}}\right)\right],$$

where

(9) $$G\equiv G_{11}G_{22}.$$

As one may note, the metric elements and the covariant Fisher matrix are both independent of the momenta. Here, it should be emphasized that we henceforth confine ourselves to Weinberg’s sign convention, in which the negative sign of the scalar curvature R is suppressed. See Refs. [25,35,36] for more details.

Having obtained the information metric, which describes a proper distance in the parameter space *X* of the nonlinear system of Equation (1), one may immediately verify, without the necessity of further calculation, that the curvature R is expressed in terms of the second and third derivatives of the nonlinear functions $\Phi_{i}\left(s_{1},s_{2}\right)$. This allows us to conclude that the scalar curvature is a natural corollary of the covariant formulation of Fisher geometry.

Table 1 summarizes the general notation employed in this work.

In addition, the results of our previous papers show clear evidence that R is a distinct quantity to investigate dynamical systems. Hence, five features of the curvature R should be noted. First, it reveals the intrinsic properties of dynamical systems, especially in the neighborhood of bifurcation points. Second, the calculation of R is, of course, invariant to any allowable transformation of coordinates. Third, phenomenologically, the sign of R provides a new measure of the local structural stability of parameter spaces. Fourth, R is in agreement with the modern theory of critical phenomena. Finally, we have demonstrated, independently of whether the sign of R is positive or negative, that |R|→∞ at the singularities of dynamical systems, and it is rigorously related to the well-known phenomena of bifurcation.

In accordance with our initial paper, Ref. [25], we proved that the scalar curvature diverges at the bifurcation points of dynamical systems described by differential equations. More specifically, the curvature scalar R shows the possibility to diverge to positive infinity when R>0, or negative infinity when R<0, as the dynamical systems under consideration approach their critical points. This naturally implies that |R|→∞, i.e., the divergent behavior of the scalar curvature is rigorously related to the phenomena of bifurcations. This interpretation is identical to the one found and discussed earlier in Ref. [25].

The divergent behavior of |R| in the neighborhood of critical points or singularities is not a new idea here, and agrees precisely with those obtained some time ago by other authors and in somewhat different contexts [35,37,38,39,40,41].

Table 2 reviews the sign and divergences of the scalar curvature for local and global bifurcations. On account of the fact that the sign of R is rigorously related to the local structural stability, as discussed earlier in Ref. [25], the positive and negative directions to infinity were analyzed for different classes of dynamical systems described by differential equations. In agreement with the results of Refs. [25,26], the curvature’s sign for the transcritical and saddle-node models are all negative, as one can readily see in Table 2. Also, R→−∞ in both models. This indicates that both bifurcations are locally structurally unstable in the neighborhood of bifurcation points.

There are two cases in Table 2 that have both positive and negative curvatures. In the supercritical pitchfork bifurcation, the curvature R diverges to positive infinity in the region −∞<m<0, in which *m* is the control parameter of the pitchfork bifurcation model. Therefore, this indicates that supercritical pitchfork bifurcations present a gain in stability in the region −∞<m<0. Nonetheless, R is negative and diverges to negative infinity in the region 0<m<+∞. Consequently, this latter behavior indicates that the system tends to lose stability in the region 0<m<+∞. In the subcritical pitchfork bifurcation, however, we have observed that R is positive when m>0, thus indicating a gain in stability. Nevertheless, R is negative when m<0, which allows us to conclude that the system loses stability in the neighborhood of the bifurcation point.

In addition to local bifurcations, we could investigate the stability and curvature R for the homoclinic bifurcations present in a Duffing-like model. The analysis of the unperturbed oscillator revealed that the sign of R is positive for homoclinic bifurcations. Furthermore, it has been demonstrated that R has bifurcation point divergence R→+∞. This result shows that homoclinic bifurcations represent smooth transitions in the parameter space *X* of Duffing’s system. Conversely, the study of the behavior of R for the perturbed oscillator reveals that the scalar curvature is mostly positive and presents signatures of not only one but rather two different transitions. The first is evident due to the positive divergence of the scalar curvature in the neighborhood of the bifurcation point. The second happens due to the change in the sign of R.

Finally, at the bottom of Table 2, we have the two-dimensional Kuramoto model, which is the most successful model for investigating spontaneous synchronization of interacting phase oscillators [26,42,43,44].

In our previous paper [26], the study of the R-diagrams for the saddle-node bifurcation of the Kuramoto model revealed that a constant R marks the synchronization of two interacting fireflies. Second, the regime of incoherence is characterized by R→0. Finally, the sign of R is always negative and R→∞ in the neighborhood of the bifurcation point (sign convention assumed). Therefore, the two-dimensional system of Kuramoto is locally structurally unstable [25].

### The Geometrical Properties of R Sign

We conclude this section by examining the geometrical properties of the sign of R. Despite the sign of the scalar curvature being physically interpreted as a new measure of stability in Ref. [25], geometrically, the sign provides the character of the trajectories in the state space. It is well known that the orbits determined by Hamiltonian equations are geodesics on a Riemannian metrical structure of the parameter space of systems described by some Kinect and potential energies [45,46,47,48].

In other words, the trajectories of the parameter space for dynamical systems of Equation (1) are geodesics in the Riemannian Fisher metric $G_{\alpha \;\mu }\left(X\right)$. Furthermore, we note, incidentally, that the state space of Equation (1) coincides with the parameter space *X* outlined above. Since the curvature R is a natural consequence of the Riemannian metrical structure, we then may characterize the trajectories of the phase space through the sign of their scalar curvature [49,50].

In particular, R>0 reveals that the orbits of the parameter space are periodic. The latter agrees with the results of our previous investigations on the homoclinic bifurcations of the Duffing oscillator.

In the study of the unperturbed Duffing oscillator [25], which is an autonomous Hamiltonian (conservative) system, the curvature’s sign is positive and has critical point divergence. That is, it is not difficult to realize that |R|→∞ at the homoclinic bifurcation point. From the geometrical interpretation of R, we thus infer that the critical point, in which the curvature becomes singular, reveals the position of the periodic trajectory. Furthermore, we may conclude that the orbits of the parameter space of the unperturbed Duffing oscillator are periodic. This latter agrees, of course, with the geometrical standpoint of the sign of the scalar curvature since a homoclinic orbit is, actually, a periodic trajectory of the infinite period [51,52].

Conversely, the non-periodic nature of the motion in the phase space can be exploited when R≤0. More specifically, R≤0 implies that the trajectories are not periodic for conservative systems. In particular, R=0 indicates signatures of parabolic orbits that separate elliptical and hyperbolic regions of the state space. However, R<0 reveals that the trajectories are hyperbolic [49,50,53].

Despite the trajectories being characterized through their Gaussian curvature’s sign in Refs. [49,50], it is important to recognize that the Ricci scalar curvature is twice the Gauss curvature for two-dimensional Riemannian manifolds. Therefore, the geometrical interpretation of the curvature sign here is the same as that of the Gaussian curvature in conservative systems and remains valid without restriction.

To conclude, we have dedicated ourselves to presenting the covariant formulation of GBT, with special emphasis on the calculation and interpretation of the curvature R. As one may conclude, GBT allows us to investigate dynamical systems through intriguing Riemannian manifolds, in which the scalar curvature is of remarkable interest.

In the framework of GBT, the curvature R becomes a powerful function because of its relation to physics, statistics, and geometry. Phenomenologically, R provides a new measure of local structural stability since R is expressed by the second and third derivatives of the nonlinear functions $\Phi_{i}\left(s_{1},s_{2}\right)$. Physically, this requires a study of the sign of R and the divergent behavior of the scalar curvature to positive or negative infinity. Statistically, R is a function of the second and the third moments of the variables of the statistical model Ω. Geometrically, the sign of R teaches us an additional and interesting feature of the Riemannian metrical structure of the parameter space *X*, that is, the character of the trajectories in the parameter space of dynamical systems described by differential equations. In particular, if R>0, this reveals that the orbits of the parameter space are periodic and the critical point in which |R|→∞ indicates the positions of the periodic trajectory.

In the next section, we apply the geometrical methods outlined here to construct a suitable characterization of limit cycles for dynamical systems in the framework of GBT.

## 3. Definition of Limit Cycles

The covariant formulation of GBT, outlined in the previous section, is here applied to study the behavior of the scalar curvature R for the elementary problem of limit cycles. Nevertheless, it is first necessary to construct a suitable covariant definition for limit cycles in the framework of GBT and extend the geometrical interpretation of the sign of R for non-conservative systems.

From CBT, a limit cycle is an isolated closed trajectory in the phase space of an autonomous system of ordinary differential equations [2,3,4,5]. According to Liouville’s theorem, a limit-cycle trajectory may only occur in dissipative dynamical systems [54]. Conservative systems, however, cannot have either locally stable equilibrium points or limit cycles but non-isolated periodic orbits in their phase space [20,54,55,56].

As we showed in Section 2, the geometrical viewpoint of the sign of R reveals the character of trajectories in parameter spaces. On account of the fact that the divergence of |R| to infinity guarantees the position and existence of periodic trajectories in a Riemannian metrical structure of the parameter space associated with conservative systems, we now ask the following fundamental questions.

First, suppose R≤0 in dissipative systems. May we infer that there are no limit cycles from the geometrical interpretation of the sign of curvature R? More specifically, what should be the nature of motion in the phase space of dissipative systems when R≤0?

Second, let R>0. Will the divergence of |R| to infinity guarantee the position and existence of limit cycles for dissipative systems?

With these questions in mind, we wish to precisely inquire whether GBT may or may not unambiguously account for the total number and positions of the limit cycles of dynamical systems described by differential equations.

To facilitate the explicit construction of the definition of limit cycles from the standpoint of GBT, we organize this section as follows. Section 3.1 is devoted to the first fundamental question raised above. Here, we dedicate ourselves to dynamical systems without limit cycles. Precisely, we aim to determine the nature of motion in the phase space when R≤0. Section 3.2 and Section 3.3 are concerned with the second fundamental question. In Section 3.2, we explore nonlinear models with only one limit cycle, while systems with more than one limit cycle are treated in Section 3.3.

Our goal here is to confirm whether GBT may account for limit cycles in the state space of autonomous systems of differential equations. In each subsection, three contrasting examples are introduced and explored. The purpose of these investigations relies on the construction of a proper and suitable definition of limit cycles by deriving several new results that have thus far been considered striking examples of the power of GBT applied to the study of nonlinear systems. The analysis is carried out with two alternative formulations, one employing the standard methods to detect limit cycles (e.g., Bendixson–Dulac criterion and Poincaré–Hopf index theorem) and the other using the approach of GBT. In this latter formulation, we investigate and interpret R. Of exceptional interest here are the cases in which R≤0, R>0 and |R|→∞, along with the geometrical interpretation of the scalar curvature’s sign. These analyses have been carried out with the aid of Mathematica 13.2.

Based on the conclusions of these investigations, we shall covariantly define limit cycles from the standpoint of GBT.

### 3.1. Systems without Limit Cycles

#### 3.1.1. First Example

As a first simple example, we have

(10) $$\left\{\begin{array}{ccc} & \frac{ds_{1}}{d\tau } & =\Phi_{1}\left(s_{1},s_{2}\right)=-3s_{1}+2s_{2},\\ & \frac{ds_{2}}{d\tau } & =\Phi_{2}\left(s_{1},s_{2}\right)=-s_{2}. \end{array}\right.$$

According to the Bendixson criterion, let the planar autonomous system above be continuously differentiable in some real domain *D*. Suppose, further, that $D\subset R^{2}$ is simply connected, i.e., there are no “separated parts” or “holes” in the present domain. Then, Equation (10) can only have periodic solutions if $\nabla \cdot \left(\Phi_{1}\left(s_{1},s_{2}\right),\Phi_{2}\left(s_{1},s_{2}\right)\right)=\partial \Phi_{1}/\partial s_{1}+\partial \Phi_{2}/\partial s_{2}$ changes sign in *D* or if it is zero in *D*. Alternatively, one may also conclude that if in the simply connected domain *D* the resulting equation of $\nabla \cdot \left(\Phi_{1}\left(s_{1},s_{2}\right),\Phi_{2}\left(s_{1},s_{2}\right)\right)$, by virtue of Equation (10), has a constant sign, that is to say, the sign remains unchanged, or $\nabla \cdot \left(\Phi_{1}\left(s_{1},s_{2}\right),\Phi_{2}\left(s_{1},s_{2}\right)\right)$ vanishes only at isolated points or on a curve, then the dynamical system, Equation (10), has no periodic trajectories [16,57,58].

Based on Equation (10), we then encounter that $\nabla \cdot \left(\Phi_{1}\left(s_{1},s_{2}\right),\Phi_{2}\left(s_{1},s_{2}\right)\right)=-4$. On account of the fact that the value of $\nabla \cdot \left(\Phi_{1}\left(s_{1},s_{2}\right),\Phi_{2}\left(s_{1},s_{2}\right)\right)$ does not change sign in any region of the phase space, we then conclude that Equation (10) does not have a limit cycle.

**Figure 1:**
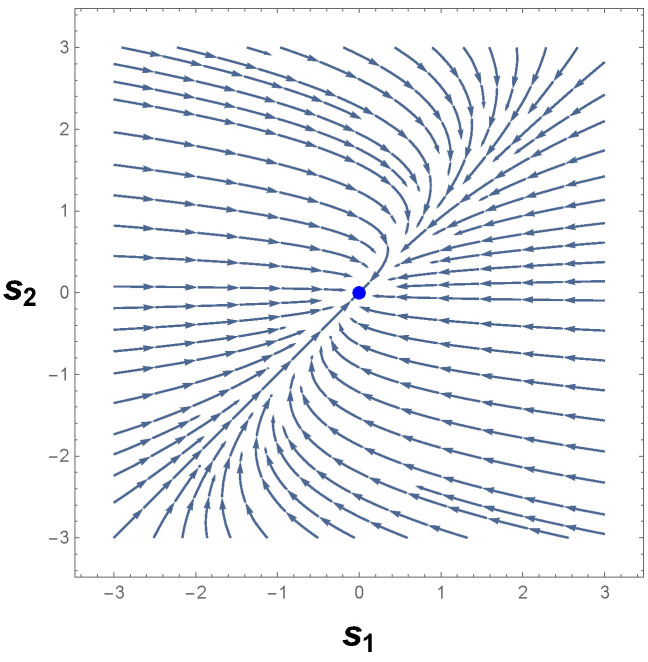
Qualitative depiction of the behavior of the phase portrait of $ds_{1}/d\tau =-3s_{1}+2s_{2}$ and $ds_{2}/d\tau =-s_{2}$ in the neighborhood of the equilibrium point $p_{0}\equiv p=\left(0,0\right)$. Here, blue dots refer to stability in the framework of CBT. As we may observe, the phase space has no limit cycles, which is in agreement with Bendixson’s criterion. In addition, one may classify the equilibrium point as a stable node. So, the trajectories are, of course, described by parabolic curves in the phase portrait.

Figure 1 shows the behavior of the phase portrait for the system of Equation (10) in the neighborhood of its equilibrium point. In the framework of CBT, it is not difficult to conclude that p=(0,0) is a stable node [16,59,60]. Consequently, the orbits approach the equilibrium point along parabolic trajectories, as we may observe in Figure 1. Hence, as one can readily see, there are no isolated closed orbits in the phase space. This result agrees with Bendixson’s criterion.

Having discussed one of CBT’s standard methods to determine whether or not there are limit cycles, we show it is also possible to predict the non-existence of limit cycles by employing our formulation of information geometry. From Equation (10), we then may write that the first example outlined above has only two varying parameters: $s_{1}$ and $s_{2}$. Therefore, the Riemannian metrical structure of the parameter space $X=(s_{1},s_{2})$ corresponds to a two-dimensional Riemannian geometry. From GBT and Equations (5)–(7), we have

(11) $$d\ell^{2}=18{\left(ds_{1}\right)}^{2}+10{\left(ds_{2}\right)}^{2},$$

which is a positive definite metric. As discussed previously in Section 2, the Fisher information metric, Equation (11), induces a scalar curvature R on the two-dimensional manifold of the parameter space *X* of Equation (10). Thus, the curvature R for our first example yields

(12) R=0,

in which we have arrived at a null scalar curvature. This latter, together with the geometrical interpretation of the sign of R, proves that the trajectories of the parameter space are not periodic for the autonomous system of Equation (10). More precisely, Equation (12) teaches us that the phase portrait of Equation (10) has parabolic trajectories with no instances of limit cycles. This result is in remarkable agreement with that obtained from the analysis of the phase portrait in Figure 1, along with the conclusions obtained from the Bendixson method.

#### 3.1.2. Second Example

As a second elementary example, we consider

(13) $$\left\{\begin{array}{ccc} & \frac{ds_{1}}{d\tau } & =\Phi_{1}\left(s_{1},s_{2}\right)=4s_{1}-s_{1}^{2}-2s_{1}s_{2},\\ & \frac{ds_{2}}{d\tau } & =\Phi_{2}\left(s_{1},s_{2}\right)=3s_{2}-s_{2}^{2}-s_{1}s_{2}. \end{array}\right.$$

According to the Bendixson criterion, Equation (13) has $\nabla \cdot \left(\Phi_{1}\left(s_{1},s_{2}\right),\Phi_{2}\left(s_{1},s_{2}\right)\right)=7-3s_{1}-4s_{2}$. Consequently, if a limit cycle exists, then it must necessarily cross the curve $7-3s_{1}-4s_{2}=0$. However, we cannot specify whether or not the system of Equation (13) has limit cycles only through that method.

Therefore, we leave this task for the Dulac criterion, which is a generalization of Bendixson’s theorem. In accordance with the theorem of Dulac, let the planar autonomous system above, Equation (13), be continuously differentiable in some real simply connected domain *D* in $\left(s_{1},s_{2}\right)$. In addition, if the nonlinear functions $\Phi_{1}\left(s_{1},s_{2}\right)$ and $\Phi_{2}\left(s_{1},s_{2}\right)\in C^{1}\left(D\right)$ and, if a function $\varphi \left(s_{1},s_{2}\right)\in C^{1}\left(D\right)$ can be found such that

(14) $$\frac{\partial \left(\varphi \Phi_{1}\right)}{\partial s_{1}}+\frac{\partial \left(\varphi \Phi_{2}\right)}{\partial s_{2}}$$

is not identically zero and has a constant sign in *D*, then the dynamical system of Equation (13) does not have limit cycles. We note, incidentally, that one retrieves the original criterion of Bendixson if $\varphi (s_{1},s_{2})=1$.

Despite the Dulac method above alternatively demonstrating the non-existence of limit cycles, one of the drawbacks of this criterion is the fact that it is not usually simple to determine a suitable $\varphi (s_{1},s_{2})$. The lack of methods and techniques to generate φ makes its possible derivation an arduous task [16,61]. That is to say, the study of limit cycles may become challenging through Dulac’s criterion, even when one knows that a particular dynamical system does not have limit cycles, because there are no general methods to determine $\varphi (s_{1},s_{2})$.

Based on the above, and after some algebraic manipulations, it is evident from Equation (13) and $\varphi \left(s_{1},s_{2}\right)=1/s_{1}s_{2}$ that

(15) $$\frac{\partial \left(\varphi \Phi_{1}\right)}{\partial s_{1}}+\frac{\partial \left(\varphi \Phi_{2}\right)}{\partial s_{2}}=-\frac{1}{s_{1}}-\frac{1}{s_{2}},$$

which is not identically zero and its sign remains unchanged in the domain *D*, where $s_{1}>0$ and $s_{2}>0$. Owing to the fact that this region of the phase space is simply connected, we thus may conclude that the dynamical system of Equation (13) does not have limit cycles.

**Figure 2:**
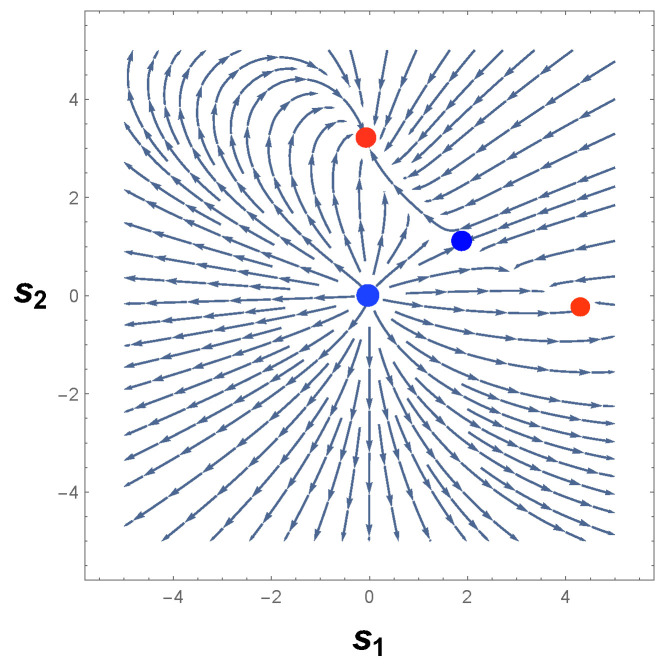
The phase space of Equation (13). Here, blue dots represent unstable equilibrium points, while red dots refer to stable points. According to the classification of equilibrium points, one may observe that the neighborhood of the saddle-node point $p_{1}=\left(2,1\right)$ is characterized by hyperbolic trajectories. Precisely, the orbits around $p_{1}$ tend to deviate from this equilibrium point through hyperbolic curves. In the neighborhood of the node equilibrium points $p_{2}=\left(0,3\right)$, $p_{3}=\left(4,0\right)$, and $p_{4}=\left(0,0\right)$, however, their respective trajectories are parabolic. In particular, one should observe that the parabolic trajectories never approach $p_{4}=\left(0,0\right)$, since this is an unstable node. Furthermore, one may realize that there are no isolated periodic orbits, which is in accordance with Dulac’s criterion.

Figure 2 shows the phase portrait for the dynamical system of Equation (13). As we may observe, there are four equilibrium points in Figure 2, in which $p_{1}=\left(2,1\right)$, $p_{2}=\left(0,3\right)$, $p_{3}=\left(4,0\right)$, and $p_{4}=\left(0,0\right)$. In accordance with CBT, we may conclude that $p_{1}=\left(2,1\right)$ is an unstable saddle-node point. Furthermore, $p_{2}=\left(0,3\right)$ and $p_{3}=\left(4,0\right)$ are classified as stable nodes, while $p_{4}=\left(0,0\right)$ is an unstable node [16,59,60]. Furthermore, one can readily see that the trajectories of this phase portrait are described by hyperbolic and parabolic orbits for the saddle and node points, respectively [59]. Therefore, there are no limit cycles.

We now examine whether it is possible to determine the existence of limit cycles through the geometrical methods of GBT. With the knowledge of Equations (5)–(7) and (13), we have

(16) $$d\ell^{2}=2\left[{\left(4-2s_{1}-2s_{2}\right)}^{2}+s_{2}^{2}\;\right]{\left(ds_{1}\right)}^{2}+2\left[4s_{1}^{2}+{\left(3-s_{1}-2s_{2}\right)}^{2}\;\right]{\left(ds_{2}\right)}^{2},$$

which is a positive invariant definite metric. The Fisher information metric, Equation (16), imposes a curvature R on the two-dimensional manifold of the parameter space *X* of the dynamical system of Equation (13). Thus, the curvature R for our second example yields.

(17) $$\begin{array}{c} R=A{\left(s_{1},s_{2}\right)}^{-1}[132+56s_{1}^{4}-382s_{2}+394s_{2}^{2}-171s_{2}^{3}+26s_{2}^{4}+6s_{1}^{3}\left(31s_{2}-47\right)\\ +s_{1}^{2}\left(608-782s_{2}+240s_{2}^{2}+s_{1}\left(1130s_{2}-716s_{2}^{2}+153s_{2}^{3}-602\right)\right)], \end{array}$$

whence,

(18) $$A\left(s_{1},s_{2}\right)={\left(-2\right)}^{-1}\left[{\left(16+4s_{1}^{2}+8s_{1}\left(s_{2}-2\right)-16s_{2}+5s_{2}^{2}\right)}^{2}{\left(5s_{1}^{2}+{\left(3-2s_{2}\right)}^{2}+s_{1}\left(4s_{2}-6\right)\right)}^{2}\right].$$

The Fisher metric gives us a scalar curvature R as a function of the derivatives of $\Phi_{1}\left(s_{1},s_{2}\right)$ and $\Phi_{2}\left(s_{1},s_{2}\right)$. We now focus on the analysis of the sign of R. As we may see, the curvature R, Equation (17), is a negative function, which implies that the dynamical system, Equation (13), does not have isolated periodic trajectories. This latter is a critical result because it agrees precisely with the conclusions extracted from the local analysis of the phase portrait, Figure 2, and Dulac method.

#### 3.1.3. Third Example

As a final example, let us consider the following dynamical system:

(19) $$\left\{\begin{array}{ccc} & \frac{ds_{1}}{d\tau } & =\Phi_{1}\left(s_{1},s_{2}\right)=s_{1},\\ & \frac{ds_{2}}{d\tau } & =\Phi_{2}\left(s_{1},s_{2}\right)=1+s_{1}+s_{2}^{2}. \end{array}\right.$$

Our last example involves a particular system in which the application of neither the Bendixson nor Dulac criteria provides conclusions about the existence of limit cycles [16]. Hence, we must employ an alternative method that enables us to determine whether Equation (19) has limit cycles and yet offers something akin to the standard criteria of Bendixson and Dulac.

We note, incidentally, that the autonomous system, Equation (19), has no equilibrium points. Hence, the desired alternative to determine the existence of limit cycles is to be found through the Poincaré–Hopf index theorem [16,62], which we shall now expand more fully.

We recall from Equations (1) and (4) that **V** designates the differentiable vector field on M and a point p∈M is said to be a singular point of **V** if V(p)=0. Bearing this in mind, the index theorem proved by Poincaré and later extended by Hopf asserts that the sum of indices at the singular points of **V** on the Riemannian manifold M is independent of **V** and is equal to the Euler–Poincaré characteristic χ(M) of M. More formally [16,62,63,64,65,66,67,68,69,70]:

**Theorem** **1.** *(Poincaré–Hopf index theorem) Let M be a compact manifold without boundary and let V be the vector field on M with only isolated singularities $p_{1},\ldots p_{k}$. Then,*

(20) $$\sum_{i=1}^{k}I_{i}\left(p_{i}\right)=\chi \left(M\right).$$

**Proof.**  A direct proof is given in Refs. [67,68,69,70]. □

In other words, if the vector field **V** has a finite number of singular points $p_{1},\ldots ,p_{k}$ of the type of sources, sinks, centers, and saddles, then the index *I* of the surface M associated with **V** is defined as the sum of the indices at each of these singular points $p_{1},\ldots ,p_{k}$ in M. In particular, Poincaré has demonstrated that saddles have an index −1, while sinks, sources, and centers have an index +1. Thus, we are led to conclude that Theorem 1 reveals surprising connections between the behavior of vector fields and the topology of manifolds. More precisely, the index of M, which is denoted by the Euler–Poincaré characteristic χ(M), is not dependent of the vector field V, but rather the topology of the surface M.

The special problem to which this particular theorem applies is that of dissipative dynamical systems possessing an isolated periodic trajectory in simply connected regions [17,68]. Precisely, Theorem 1 demonstrates that the index of an isolated closed trajectory is the algebraic sum of the indices of the enclosed singular points. Hence, the existence of a limit cycle requires at least one equilibrium point, and if the dynamical system under consideration possesses a unique singular point *p*, then the index of *p*, I(p), cannot be negative. That is to say, *p* cannot be of a saddle-type equilibrium point [71,72].

Armed with this theorem, we turn back to the vector field of Equation (19). It is not difficult to recognize that Equation (19) has no equilibrium points. Thus, one may conclude, without the necessity of the analysis of the phase portrait, that our last example indeed has no limit cycles as an immediate consequence of the Poincaré–Hopf theorem [73].

Having discussed the standard methods to determine the existence of limit cycles, we now dedicate ourselves to the covariant formulation of GBT. From Equation (19) and the general rules developed in Section 2, we obtain the following invariant positive definite metric:

(21) $$d\ell^{2}=4{\left(ds_{1}\right)}^{2}+4s_{2}^{2}{\left(ds_{2}\right)}^{2},$$

which induces a null curvature R on the manifold of the parameter space *X* of Equation (21). This result appropriately states that our last example has no limit cycles. Further, it is consistent with conclusions obtained from the Poincaré index theorem and demonstrates that dissipative systems with R≤0 have no limit cycles from the geometrical interpretation of the sign of R.

To conclude, we have investigated three contrasting examples of dynamical systems throughout this subsection. Here, the non-existence of limit cycles has been demonstrated by two alternative formulations. First, we have employed the standard approaches to detect limit cycles through the Bendixson–Dulac criterion and Poincaré–Hopf index theorem. Second, we have considered the geometrical methods of GBT.

Despite the standard methods being reasonably accurate in determining the non-existence of limit cycles, these suffer from drawbacks and impose several challenges. First, the Bendixson criterion neither excludes nor guarantees the existence of limit cycles in phase spaces even if $\nabla \cdot \left(\Phi_{1}\left(s_{1},s_{2}\right),\Phi_{2}\left(s_{1},s_{2}\right)\right)$ is zero (we prove this in the next subsection). Second, Dulac’s method requires a particular function $\varphi (s_{1},s_{2})$, so that one may conclude that there are no limit cycles. As we have outlined, there are no standard methods or techniques to generate such functions, even if it is known that the dynamical systems under study have no limit cycles. Third, if an autonomous system has more than one equilibrium point, then the corollaries of Poincaré index theorem do not allow us to determine the exact locations of limit cycles. Finally, all the standard methods to investigate limit cycles outlined above do not provide the actual number of the existing limit cycles, either their possible existence in non-simply connected regions or their accurate positions. Nevertheless, our geometrical treatment surprisingly demonstrates that autonomous dynamical systems do not have limit cycles when their associated scalar curvature R is either negative or zero, that is, R≤0.

This result provides an affirmative answer to the first fundamental question raised above. Consequently, we may write that if R≤0 in dissipative systems, there are, indeed, no limit cycles, accordingly to the geometrical viewpoint of the sign of R. More specifically, R=0 teaches us that the trajectories of the parameter space are parabolic. However, a negative curvature R reveals that the orbits in the phase space of dissipative systems could be either hyperbolic or parabolic or both. This agrees with the nature of motions of the state space of our second example, in which the existence of both hyperbolic and parabolic orbits in the neighborhood of the equilibrium points of Equation (13) became evident. Furthermore, we may conclude that the covariant formulation of GBT expands the state of the art because the geometric methods outlined here introduce an alternative approach to determine the non-existence of limit cycles.

Based on the above, we now prove that our geometric formulation also allows us to predict the position and actual number of limit cycles in the phase space of dynamical systems described by differential equations.

### 3.2. Systems with Only One Limit Cycle

Here, we will first dedicate ourselves to the nonlinear models with one limit cycle, closely following the developments of Section 2.

As we have mentioned, the sign of R reveals intrinsic properties of the parameter space, that is, the character of the orbits of conservative dynamical systems in the phase space. In particular, if the sign of scalar curvature is positive and has critical point divergence, i.e., |R|→∞, then one may conclude that periodic trajectories characterize the parameter space of the dynamical system under consideration. After the discussion in Section 3.1, one might now well ask whether the divergence of |R| to infinity guarantees the position and existence of limit cycles for dissipative systems.

To answer this, we must demonstrate that GBT can account for limit cycles in the state space of autonomous systems of differential equations. To this end, we start with nonlinear differential equations with only one limit cycle. Systems with more than one limit cycle are discussed in Section 3.3.

#### 3.2.1. First Example

As the first example of a dynamical system with one limit cycle, we start with the van der Pol equation. This particular mathematical model, which is a special case of the Liénard equation, is introduced to represent the effects of the auto-oscillations of a generator on a triode for a tube with a cubic characteristic [16,74].

Despite being one of the simplest oscillating systems, the van der Pol oscillator does lead to a qualitative understanding of the concept of limit cycles. The nonlinear differential equations of the van der Pol oscillator may be written explicitly in the form

(22) $$\left\{\begin{array}{ccc} & \frac{ds_{1}}{d\tau } & =\Phi_{1}\left(s_{1},s_{2}\right)=s_{2},\\ & \frac{ds_{2}}{d\tau } & =\Phi_{2}\left(s_{1},s_{2}\right)=-\left(s_{1}^{2}-1\right)s_{2}-s_{1}. \end{array}\right.$$

From the criterion of Bendixson and Equation (22), we have

(23) $$\nabla \cdot \left(\Phi_{1}\left(s_{1},s_{2}\right),\Phi_{2}\left(s_{1},s_{2}\right)\right)=1-s_{1}^{2}.$$

**Figure 3:**
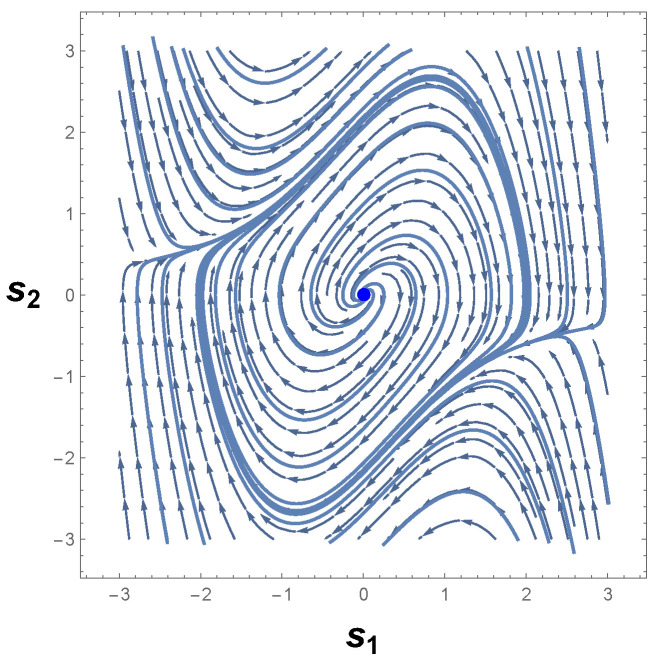
The phase portrait of the van der Pol equation. Here, blue dots refer to unstable equilibrium points. As one can readily see, there is indeed a limit cycle in the neighborhood of p=(0,0), which agrees with the index theorem. In addition, the periodic trajectory crosses the lines $s_{1}=\pm 1$, which is in agreement with the theorem of Bendixson. While we have qualitatively confirmed the presence of this limit cycle from the analysis of the phase space and the standard methods discussed previously, one may also infer the existence of isolated periodic orbits in Liénard’s equations from the theorems of Liénard, Levinson, and Smith.

Therefore, we are led to conclude that if a limit cycle exists, then it must necessarily cross the curve $-\left(s_{1}^{2}-1\right)=0$. Figure 3 shows the phase portrait of Equation (22). As one can readily see, there is indeed a limit cycle that involves the unstable equilibrium point $p_{0}\equiv$p=(0,0). This result is in accordance with Poincaré–Hopf index theorem since an isolated periodic trajectory of the phase space must contain at least one equilibrium point.

Furthermore, it should be noted that the limit cycle crosses the lines $s_{1}=\pm 1$ in Figure 3. According to the Bendixson criterion, if a limit cycle exists in a simply connected region, then this limit cycle must, necessarily, cross the curve in which $\nabla \cdot \left(\Phi_{1}\left(s_{1},s_{2}\right),\Phi_{2}\left(s_{1},s_{2}\right)\right)=0$. As one may realize,

(24) $$\nabla \cdot \left(\Phi_{1}\left(s_{1},s_{2}\right),\Phi_{2}\left(s_{1},s_{2}\right)\right)=0\;\mathrm{at}\;s_{1}=\pm 1.$$

From Equation (24), it follows that the periodic trajectory of the phase portrait, Figure 3, indeed represents a limit cycle in agreement with the Bendixson criterion and the theorems proved by Liénard, Levinson, and Smith [75,76].

Having examined the existence of limit cycles from the standpoint of standard methods, let us turn our attention to the examination of limit cycles in the framework of GBT. We start from the covariant formulation of GBT in Section 2. With the knowledge of Equations (22) and (5)–(7), we obtain the following positive definite metric

(25) $$d\ell^{2}=2{\left[-1-2s_{1}s_{2}\right]}^{2}{\left(ds_{1}\right)}^{2}+2\left[1+{\left(1-s_{1}^{2}\right)}^{2}\right]{\left(ds_{2}\right)}^{2}.$$

The Fisher information metric above, Equation (25), naturally imposes a curvature R on the two-dimensional manifold of the phase space *X* of the dynamical system of Equation (22). Following the procedure given in Section 2, we obtain the curvature R for the van der Pol oscillator in terms of the second and third derivatives of the nonlinear functions $\Phi_{i}$:

(26) $$R=\frac{2\left(-2+6s_{1}^{2}-3s_{1}^{4}+s_{1}^{6}+4s_{2}s_{1}^{3}\right)}{{\left(2-2s_{1}^{2}+s_{1}^{4}\right)}^{2}{\left(1+2s_{1}s_{2}\right)}^{3}}.$$

From the knowledge of R, we now may determine the character of the trajectories of the phase space associated with Equation (22) from the standpoint of GBT. Precisely, we determine whether or not the divergence of the scalar curvature to infinity may account for the total number and positions of the limit cycles in the state space of autonomous dissipative systems. To exploit this and provide an affirmative answer to the second fundamental question raised above, we dedicate ourselves to the study of the positive sign and divergent behavior of curvature R, Equation (26), following the same procedure used in Refs. [25,26].

To discuss the positivity of R, as defined by Equation (26), in the framework of Riemannian geometry, it is convenient, for this purpose, to consider the geometric counterpart of the Poincaré index theorem. More precisely, the Gauss–Bonnet theorem.

The well-known theorem of Gauss and Bonnet states that for any compact, oriented, and boundaryless two-dimensional Riemannian manifold M, the Euler–Poincaré characteristic, χ(M), becomes the integral of the Gaussian curvature over the entire manifold M. However, on account of the fact that our statistical model carries the structure of a smooth two-dimensional Riemannian manifold M, the scalar curvature is twice the Gaussian curvature. Consequently, this allows us to state the Gauss–Bonnet theorem more formally as follows [34,67,77,78,79].

**Theorem** **2.** *(Gauss–Bonnet theorem) Let $M^{2}$ be a two-dimensional compact oriented differentiable manifold. Let V be a differentiable vector field on M with isolated singularities $p_{1},\ldots ,p_{k}$ whose indices are $I_{1},\ldots ,I_{k}$. Then, for any Riemannian metric on M,*

(27) $$\frac{1}{4\pi }\int_{M}R\left(s_{1},s_{2}\right)\sqrt{G}ds_{1}ds_{2}=\sum_{i=1}^{k}I_{i}\left(p_{i}\right)=\chi \left(M\right).$$

**Proof.**  The proof of this remarkable theorem is most transparent and given in detail in Ref. [67]. □

This theorem, together with the Poincaré–Hopf index theory, represents one of the most important contributions of mathematics. More specifically, the Gauss–Bonnet theorem provides a deep connection between information geometry and topology since it relates the R of $M^{2}$ to its Euler characteristic. In other words, this theorem is a natural consequence of the Poincaré–Hopf index theorem presented earlier [34,67,77,78,79].

One of the special applications to which this theorem applies is that of the positivity of scalar curvatures [80]. Precisely, if M is a closed, that is, compact, with no boundary or boundaryless two-dimensional manifold, then the Gauss–Bonnet theorem states that $M^{2}$ may exhibit a positive scalar curvature only if its Euler characteristic is positive. Therefore, since this holds for any compact, boundaryless two-dimensional manifold, $M^{2}$, we then obtain the positivity condition for the curvature R,

(28) $$R>0\;\mathrm{for}\;\chi \left(M\right)=\sum_{i=1}^{k}I_{i}>0.$$

This basically teaches us that if one demonstrates that the Euler characteristic is positive, that is, the sum of indices at each of the singular points $p_{1},\ldots ,p_{k}$ of the vector field V that characterizes the dynamical system under study is positive, then the condition expressed by Equation (28) assures the positivity of R for the respective dynamical system.

Thus, in order to carry out this program, we must determine the nature of $p_{i}$ and its respective indices following the Poincaré–Hopf index theorem. This can be very simply accomplished by analyzing the trace and determinant of the Jacobian matrix of Equation (4) evaluated at its singular points $p_{i}$.

With these lessons in mind, we now turn back to Equation (22). As we saw earlier, the van der Pol oscillator possesses a unique and unstable equilibrium point $p_{1}=\left(0,0\right)$. The Jacobian matrix of Equation (22) at $p_{1}$ is trivially evaluated as follows:

(29) $$J\left(p_{1}\right)=\left[\begin{array}{cc} 0 & 1\\ -1 & 1 \end{array}\right].$$

With the aid of this result, one may recognize that both the trace and determinant of J are equal to one. We must recall that if detJ<0, then the singular point is a saddle. However, if detJ>0 and $\mathrm{Tr}\;J^{2}-4\left(\det\;J\right)>0$, the equilibrium point is a node. Finally, if detJ>0 and $\mathrm{Tr}\;J^{2}-4\left(\det\;J\right)<0$, then the equilibrium point is said to be a source equilibrium point for TrJ>0, a sink for TrJ<0, and a center for TrJ=0; see Refs. [16,81] for more details.

In light of the foregoing, $p_{1}=\left(0,0\right)$ denotes a source equilibrium point. Hence, we immediately identify, from the Poincaré–Hopf index theorem, the index $I\left(p_{1}\right)$ or the Euler characteristic of M to be

(30) $$\chi \left(M\right)=I_{1}\left(p_{1}\right)=1.$$

Owing to the fact that χ(M)>0, together with the positivity condition of Equation (28), we thus are led to conclude that the scalar curvature R of $M^{2}$, Equation (26), is a positive multivariable scalar function.

**Figure 4:**
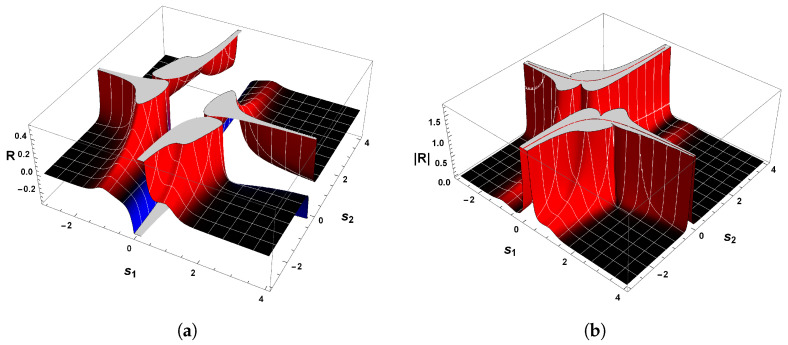
(**a**) shows the behavior of R as a function of $s_{1}$ and $s_{2}$ for variations in ($s_{1}$, $s_{2}$) in the van der Pol oscillator. Following the sign convention of Ref. [25], the red and blue colors represent positive and negative values of scalar curvature, Equation (26), respectively. However, the black color refers to null values of R. As one can readily see, the curvature R is mostly positive in the neighborhood of the singular points $p_{\pm }^{C}=\left(\pm 1,\mp 1/2\right)$. This indicates that the van der Pol oscillator has a periodic trajectory in the phase space. (**b**) depicts the magnitude of this scalar curvature, in which it is evident that |R|→∞ at $p_{\pm }^{C}=\left(\pm 1,\mp 1/2\right)$. In as much as these singular points violate the Bendixson criterion, the van der Pol oscillator has a unique limit cycle in its phase space.

The correctness of the sign of R can be confirmed by analyzing Figure 4a, which shows the behavior of scalar curvature for the van der Pol oscillator. Indeed, evaluations with Equation (26) reveal that the sign of curvature R is uniformly positive in the vicinity of p=(0,0). So, we may conclude that the van der Pol oscillator, Equation (23), indeed has a periodic trajectory in the phase space, according to the geometrical interpretation with regards to the sign of R.

Another remark concerning this oscillator is the divergence of the scalar curvature and its connection with limit cycles. From Equation (26), it is evident that the magnitude of Equation (26) becomes singular in the neighborhood of p=(0,0), as illustrated in Figure 4b. In general, the exact critical point in which |R|→∞ is to be found when the denominator of the scalar curvature, Equation (26), is zero [25,26,82]. In light of the foregoing, we may realize that |R| diverges to positive infinity when

(31) $${\left(2-2s_{1}^{2}+s_{1}^{4}\right)}^{2}{\left(1+2s_{1}s_{2}\right)}^{3}=0.$$

The problem of finding the exact roots or zeros of Equation (31) is not usually a simple task and admits several possible methods [83,84,85,86,87]. However, an inspection of Equation (31) shows that the denominator of curvature R is zero at $\left(\pm 1,\mp 1/2\right)$ by taking into account Equation (24). Consequently, it naturally follows that

(32) $$\left|R\right|\to \infty \;\mathrm{as}\;p_{1,2}^{C}\to \left(\pm 1,\mp \frac{1}{2}\right),$$

where we have introduced $p_{i}^{C}$ to denote the critical points or the singularities of |R|.

This last result teaches us that the magnitude of the scalar curvature diverges to infinity at the states $p_{1,2}^{C}\equiv p_{\pm }^{C}=\left(\pm 1,\mp 1/2\right)$, which are symmetrical singularities with respect to the origin. In addition, as one may realize, the singular points of |R| only differ by sign. Hence, it is not difficult to conclude that these two symmetrical points are mirror images of each other. With this lesson in mind, we now turn back to Equation (23), in which the Bendixson criterion has been applied to van der Pol’s differential equations. Accordingly, if a limit cycle exists in a simply connected region, then this isolated periodic orbit must, necessarily, cross the curve in which $\nabla \cdot \left(\Phi_{1}\left(s_{1},s_{2}\right),\Phi_{2}\left(s_{1},s_{2}\right)\right)=0$. A comparison with the resultant Equations (24) and (32) allows us to conclude that

(33) $$\begin{array}{cc} & \left|R\right|\to \infty \;\mathrm{and}\;\nabla \cdot \left(\Phi_{1}\left(s_{1},s_{2}\right),\Phi_{2}\left(s_{1},s_{2}\right)\right)\to 0\\ & \mathrm{as}\;p_{\pm }^{C}\to \left(\pm 1,\mp \frac{1}{2}\right). \end{array}$$

This result naturally suggests that a dissipative dynamical system with only one limit cycle is one in which its scalar curvature is positive and diverges to positive infinity at symmetrical singular points. In addition, this finding agrees with the analysis of the phase portrait for the van der Pol oscillator along with the conclusions extracted from the theorem of Bendixson. Evidently, if $p_{\pm }^{C}=\left(\pm 1,\mp 1/2\right)$ satisfies both Equations (24) and (32), together with the sign interpretation of R, then one may infer that the singular points of R are the same points that violate Bendixson’s criterion. This offers a possible and clearly superior alternative to demonstrate the existence of a single limit cycle in the phase space of autonomous dynamical systems described by differential equations.

But, before we construct the proper answer to the second fundamental question raised above, we supply additional evidence concerning the existence of single isolated periodic orbits in the framework of GBT. To enforce the conclusions derived above, let us now look at a few more examples.

#### 3.2.2. Second Example

As a second elementary dissipative system with only one limit cycle, we have the following Liénard system [16,20,88]:

(34) $$\left\{\begin{array}{ccc} & \frac{ds_{1}}{d\tau } & =\Phi_{1}\left(s_{1},s_{2}\right)=s_{2},\\ & \frac{ds_{2}}{d\tau } & =\Phi_{2}\left(s_{1},s_{2}\right)=-s_{1}-s_{2}\left(s_{1}^{4}-1\right), \end{array}\right.$$

in which $p_{1}=\left(0,0\right)$ is an unstable equilibrium point. By employing the Bendixson criterion, one may verify that

(35) $$\nabla \cdot \left(\Phi_{1}\left(s_{1},s_{2}\right),\Phi_{2}\left(s_{1},s_{2}\right)\right)=1-s_{1}^{4}.$$

From Equation (35) it follows that if there is an isolated periodic trajectory in a simply connected region, then $\nabla \cdot \left(\Phi_{1}\left(s_{1},s_{2}\right),\Phi_{2}\left(s_{1},s_{2}\right)\right)=0$. From this, it naturally follows that

(36) $$\nabla \cdot \left(\Phi_{1}\left(s_{1},s_{2}\right),\Phi_{2}\left(s_{1},s_{2}\right)\right)=0\;\mathrm{at}\;s_{1}=\pm 1.$$

**Figure 5:**
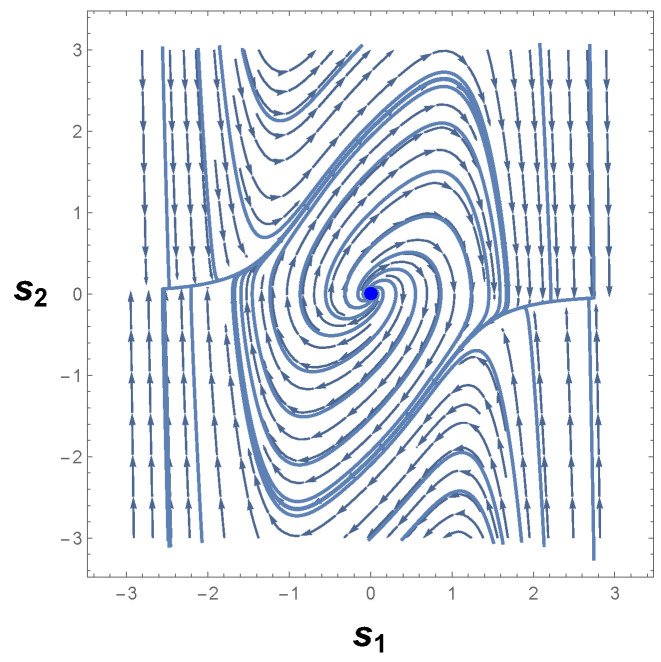
The behavior of the phase portrait of Equation (34). Here, blue dots refer to unstable equilibrium points. It is evident that there exists a limit cycle in the neighborhood of $p_{1}=\left(0,0\right)$, which is in agreement with the Poincaré–Hopf index theorem. Further, the lines $s_{1}=\pm 1$ are crossed by the isolated periodic trajectory, which agrees with the Bendixson theorem.

Figure 5 depicts the behavior of Equation (34). As we may see, the phase portrait of our second example shows an isolated periodic orbit that crosses the lines $s_{1}=\pm 1$, which agrees with the Bendixson theorem. In addition, this periodic curve encloses $p_{1}=\left(0,0\right)$, which agrees with the Poincaré–Hopf index theorem. Based on the above, we may thus conclude that the phase space of Equation (35) has a limit cycle.

Having examined the existence of isolated periodic trajectories from the standard methods of CBT, let us turn our attention to the examination of limit cycles in the framework of the covariant formulation of GBT. From Equation (34), it is not difficult to recognize that the Liénard system above has only two varying parameters: $s_{1}$ and $s_{2}$. So, the Riemannian metrical structure of the parameter space $X=(s_{1},s_{2})$ corresponds to a two-dimensional Riemannian geometry. Bearing this in mind, we work forward from GBT by deriving the invariant positive definite metric. From Equations (5)–(7), we have

(37) $$d\ell^{2}=2{\left[-1-4s_{1}^{3}s_{2}\;\right]}^{2}{\left(ds_{1}\right)}^{2}+2\left[1+{\left(1-s_{1}^{4}\right)}^{2}\;\right]{\left(ds_{2}\right)}^{2}.$$

The metric Equation (37) imposes a curvature R on the two-dimensional parameter space *X* of the Liénard system above. From Equations (8) and (34), we then encounter

(38) $$R=\frac{4s_{1}^{2}\left(-6+16s_{1}^{4}-9s_{1}^{8}+3s_{1}^{12}+16s_{1}^{7}s_{2}\right)}{{\left(2-2s_{1}^{4}+s_{1}^{8}\right)}^{2}{\left(1+4s_{1}^{3}s_{2}\right)}^{3}}.$$

Armed with the Riemannian metric and its correspondent scalar curvature R, we now seek to determine the character of the trajectories in the phase space of Equation (34). Of exceptional interest are the sign and divergent behavior of |R|.

To discuss the positivity of Equation (38), we must demonstrate that χ(M)>0. Our procedure will be precisely analogous to that of the van der Pol oscillator. First, we determine the nature of the equilibrium points of Equation (34) and their respective indices, following the Poincaré–Hopf index theorem. Second, we devote ourselves to the computation of the Euler-characteristic number χ(M). Finally, the sign of R is interpreted with the aid of the positivity condition expressed by Equation (28).

To carry out this program, we must observe that Equation (34) has a unique and unstable equilibrium point. The Jacobian matrix of Equation (34) at *p* is simply

(39) $$J\left(p_{1}\right)=\left[\begin{array}{cc} 0 & 1\\ -1 & 1 \end{array}\right],$$

whence one may infer again that both the trace and determinant of J are equal to one. Owing to the fact that detJ>0 and $\mathrm{Tr}\;J^{2}-4\det\;J<0$, then $p_{1}=\left(0,0\right)$ designates a source equilibrium point. As a final step, we immediately identify, from Theorem 2, the index of $p_{1}$ or the Euler characteristic of M to be

(40) $$\chi \left(M\right)=I_{1}\left(p_{1}\right)=1.$$

Since Equations (28) and (40) assure us that R>0, the necessary property of the positivity of the scalar curvature is demonstrated.

**Figure 6:**
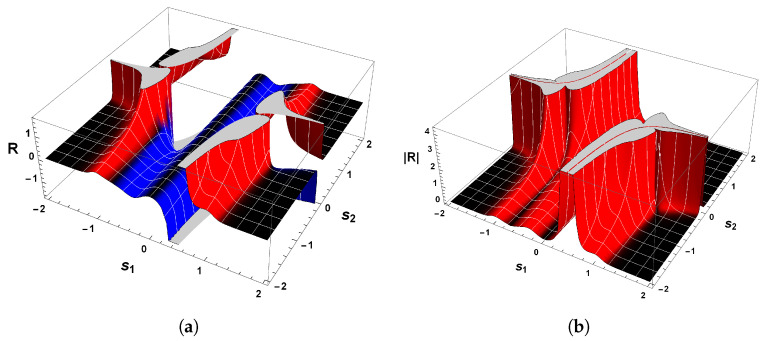
(**a**) presents the behavior of R as a function of $s_{1}$ and $s_{2}$ for variations in ($s_{1}$, $s_{2}$) for our second example of Equation (34). Following the sign convention of Ref. [25], the red and blue colors represent positive and negative values of the scalar curvature, respectively. The black color refers to null values of the curvature. The curvature R is positive in the neighborhood of the singular points $p_{\pm }^{C}=\left(\pm 1,\mp 1/4\right)$. Thus, one may conclude that periodic orbits characterize the corresponding phase portrait of Equation (34). (**b**) shows the magnitude of the curvature R, in which it naturally follows that |R|→∞ at $p_{\pm }^{C}=\left(\pm 1,\mp 1/4\right)$. Since these singular points violate Bendixson’s criterion, one may conclude that Equation (34) has only one limit cycle. This result agrees with the analysis of the phase portrait and the Bendixson criterion.

Figure 6 shows the behavior of R for our second example. Evaluations with Equation (38) show that the curvature R is positive in the vicinity of the equilibrium point p=(0,0), which confirms the statement that guarantees the positivity of R. Hence, the state space of Equation (34) has periodic trajectories, which agrees with the analysis of the phase portrait above.

A further remark concerns the divergent behavior of the scalar curvature of Equation (34). From the equation of R, we may recognize that Equation (38) is singular in the neighborhood of the equilibrium point p=(0,0). By utilizing methods identical to those employed in our last example and Refs. [25,26,82], we are led to conclude that |R|→∞ when the denominator of Equation (38) is zero. With the aid of Equation (36), it naturally follows that

(41) $$\left|R\right|\to \infty \;\mathrm{as}\;p_{\pm }^{C}\to \left(\pm 1,\mp \frac{1}{4}\right).$$

This result coincides with that found for the van der Pol oscillator. That is to say, the scalar curvature of our second example, Equation (34), also diverges to positive infinity at symmetrical critical points with respect to the origin.

Before proceeding to the discussion of this result, let us compare Equation (41) with the one obtained by the Bendixson criterion. As we have seen, Equation (35) informs us that if a limit cycle exists in a simply connected region, then this isolated periodic trajectory must, necessarily, cross the curve in which $\nabla \cdot \left(\Phi_{1}\left(s_{1},s_{2}\right),\Phi_{2}\left(s_{1},s_{2}\right)\right)=0$. Therefore, a comparison with the divergent behavior of R for our second example and the resultant Equation (35) yields

(42) $$\begin{array}{cc} & \left|R\right|\to \infty \;\mathrm{and}\;\nabla \cdot \left(\Phi_{1}\left(s_{1},s_{2}\right),\Phi_{2}\left(s_{1},s_{2}\right)\right)\to 0\\ \; & \mathrm{as}\;p_{\pm }^{C}\to \left(\pm 1,\mp \frac{1}{4}\right). \end{array}$$

On account of the fact that $p_{\pm }^{C}=\left(\pm 1,\mp 1/4\right)$ satisfies both Equations (41) and (32), then this result again answers affirmatively the second fundamental question raised above, since the singular points of the scalar curvature are the same points that violate Bendixson’s criterion. Consequently, we thus may write that our second example has a unique limit cycle. In addition, the result expressed by Equation (42) enforces the understanding that dissipative dynamical systems with only one limit cycle are those in which the scalar curvature is positive and whose magnitude diverges to infinity at symmetrical singular points with respect to the origin. This agrees with the analysis of the phase portrait of Equation (34) and the Bendixson criterion.

As a final remark, one may note that our first and second examples belong to the family of Liénard systems. Therefore, the geometrical results found in our second example were somewhat expected as both systems, Equations (22) and (34), share almost the same qualitative dynamical properties.

Hence, we conclude this subsection by examining a final different example that does not belong to the family of Liénard systems, and yet, no conclusions can be extracted about the possible existence of limit cycles from the Bendixson criterion.

#### 3.2.3. Third Example

Let us consider the following autonomous dissipative system:

(43) $$\left\{\begin{array}{ccc} & \frac{ds_{1}}{d\tau } & =\Phi_{1}\left(s_{1},s_{2}\right)=s_{1}s_{2}^{2}-s_{1}-s_{2},\\ & \frac{ds_{2}}{d\tau } & =\Phi_{2}\left(s_{1},s_{2}\right)=s_{2}^{3}+s_{1}-s_{2}. \end{array}\right.$$

Upon applying the Bendixson criterion to Equation (43), we then encounter

(44) $$\nabla \cdot \left(\Phi_{1}\left(s_{1},s_{2}\right),\Phi_{2}\left(s_{1},s_{2}\right)\right)=-2+4s_{2}^{2}.$$

Although this criterion indicates that our last example does not have isolated periodic orbits in either half-plane $s_{2}<-1/\sqrt{2}$ or $s_{2}>1/\sqrt{2}$, one should resist the impulse to conclude that there are no limit cycles in the phase space of Equation (43).

**Figure 7:**
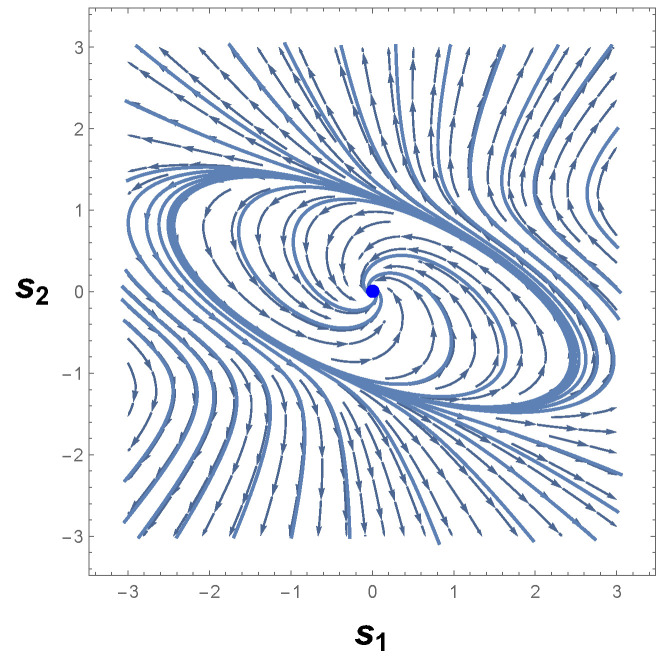
The phase portrait of the nonlinear system expressed by Equation (44). Here, the blue dot refers to an unstable equilibrium point. From Bendixson’s negative theorem, we may infer that there are no isolated periodic orbits in either half-plane $s_{2}<\pm 1/\sqrt{2}$ or $s_{2}>\pm 1/\sqrt{2}$. Nevertheless, it is evident that Equation (44) has an elliptical limit cycle. This result indicates that Bendixson’s criterion cannot either guarantee the existence or exclude the possibility of limit cycles in the phase space of Equation (43).

Figure 7 shows the behavior of our last example, Equation (43), in which we may observe an elliptic isolated periodic trajectory that involves the unstable equilibrium point $p_{1}=\left(0,0\right)$. Despite being in accordance with the index theorem of Poincaré and Hopf, we note that this result is in apparent contradiction with Bendixson’s theorem. That is to say, the criterion of Bendixson guarantees that there are no isolated periodic orbits in either half-plane $s_{2}<1/\sqrt{2}$ or $s_{2}>1/\sqrt{2}$. However, the analysis of the phase portrait reveals a limit cycle in the neighborhood of $p_{1}=\left(0,0\right)$.

This contradiction may be resolved by more careful consideration of the axioms of the Bendixson and Dulac theorems. It should be remarked that the application of these negative criteria does not assure the non-existence of closed trajectories in the whole phase space of Equation (43) since there may be such a periodic trajectory that crosses the lines $s_{2}=\pm 1/\sqrt{2}$. In fact, the Bendixson–Dulac criterion, along with the Poincaré–Hopf index theorem, only allows one to infer the existence of limit cycles in simply connected regions. Nevertheless, these neither exclude, guarantee, or assure isolated closed trajectories in non-simply connected regions [16,57,61,75].

Hence, we may conclude that the dissipative dynamical system, Equation (43), indeed presents a limit cycle in a non-simply connected region of the phase portrait, even if the existence of such an isolated closed trajectory is undetermined from the standpoint of the negative theorem of Bendixson.

Having explored the last example of this subsection from the standard methods of CBT, let us verify whether the existence of this elliptical limit cycle could be determined in the framework of GBT.

It is evident from Equation (43) that our third example has only two varying parameters: $s_{1}$ and $s_{2}$. Thus, the Riemannian metrical structure of the parameter space $X=(s_{1},s_{2})$ yields a two-dimensional Riemannian geometry. Based on the above, the positive definite Riemannian metric of the parameter space of Equation (43), with the aid of Equations (5)–(7), is given by

(45) $$d\ell^{2}=2\left[1+{\left(s_{2}^{2}-1\right)}^{2}\right]{\left(ds_{1}\right)}^{2}+2\left[{\left(-1+2s_{1}s_{2}\right)}^{2}+{\left(3s_{2}^{2}-1\right)}^{2}\right]{\left(ds_{2}\right)}^{2}.$$

As we saw earlier, it is implied by Equation (45) that this Fisher metric induces a curvature R on the manifold of the parameter space *X* of the dynamical system of Equation (43). By Equation (8), we thus have

(46) $$R=\left[\frac{-8+2s_{2}\left(8s_{1}^{2}s_{2}^{3}-2s_{1}\left(-2+8s_{2}^{2}-3s_{2}^{4}+s_{2}^{6}\right)+s_{2}\left(16-28s_{2}^{2}+46s_{2}^{4}-21s_{2}^{6}+9s_{2}^{8}\right)\right)}{{\left(2-2s_{2}^{2}+s_{2}^{4}\right)}^{2}{\left(2+s_{2}\left(-4s_{1}-6s_{2}+4s_{1}^{2}s_{2}+9s_{2}^{3}\right)\right)}^{2}}\right].$$

From the knowledge of R, we study its sign and divergent behavior. The sign of Equation (46) follows from the condition expressed in terms of the Euler characteristic, Equation (28). Proceeding in a manner parallel to that of the van der Pol oscillator and Liénard system studied above, we first obtain the nature of the equilibrium points associated with Equation (43) and their respective indices, closely following the index theorem of Poincaré. Second, we devote ourselves to the computation of the Euler-characteristic number χ(M). Finally, we interpret the sign of scalar curvature through the positivity condition. It should be noted that Equation (43) possesses a unique and unstable equilibrium point $p_{1}=\left(0,0\right)$. From Equation (43) and $p_{1}=\left(0,0\right)$, the Jacobian matrix is directly evaluated as follows:

(47) $$J\left(p_{1}\right)=\left[\begin{array}{cc} -1 & -1\\ 1 & -1 \end{array}\right].$$

From which, it is not difficult to realize that the TrJ=−2 and detJ=2. Hence, this immediately teaches us that $p_{1}=\left(0,0\right)$ is a sink. From the Poincaré–Hopf index theorem, we identify that the index $I_{1}\left(p_{1}\right)$ or the Euler characteristic of M is

(48) $$\chi \left(M\right)=I_{1}\left(p_{1}\right)=1.$$

**Figure 8:**
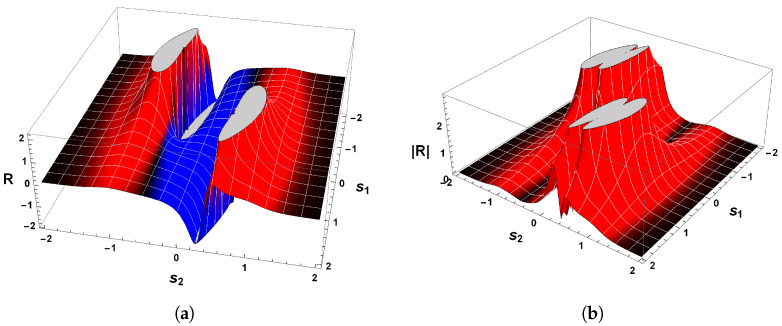
(**a**) presents the behavior of R as a function of $s_{1}$ and $s_{2}$ for variations in ($s_{1}$, $s_{2}$) for our last example of Equation (34). Following the sign convention of Ref. [25], the red and blue colors represent positive and negative values of the scalar curvature, respectively. The black color refers to null values of the curvature. The curvature R is positive in the neighborhood of the equilibrium points, which informs us that the phase portrait of Equation (34) is characterized by periodic trajectories. In addition, |R| becomes singular at the $p_{\pm }^{C}=\left(\pm \sqrt{3}/2,\mp 1/\sqrt{3}\right)$, as illustrated in (**b**). Since the singular points of R are the same as those that violate the Bendixson criterion, we then may conclude that Equation (34) has a single limit cycle. This result agrees with the analysis of the phase portrait and the Bendixson criterion.

On account of the fact that χ(M)>0, then we have thereby demonstrated the positiveness of the scalar curvature. Figure 8a shows the sign of the scalar curvature associated with Equation (43). As we may observe, the curvature R is real and positive in the physical regime of $s_{2}<-1/\sqrt{2}$ or $s_{2}>1/\sqrt{2}$. This confirms the consistency of the positivity condition for the sign of the scalar curvature. Furthermore, it is remarkable that |R| diverges to positive infinity when the denominator of Equation (46) is zero, as illustrated in Figure 8b. From this it naturally follows that

(49) $$\left|R\right|\to \infty \;\mathrm{as}\;p_{\pm }^{C}\to \left(\pm \frac{\sqrt{3}}{2},\mp \frac{1}{\sqrt{3}}\right).$$

This result makes it evident again that the trajectories of the phase space for our last example, which is a dissipative dynamical system, are described by an isolated periodic orbit, according to the geometrical interpretation of the sign of R.

Due to the fact that the two symmetrical singularities $p_{\pm }^{C}=\left(\pm \sqrt{3}/2,\mp 1/\sqrt{3}\right)$ satisfy both Equations (32) and (41), then we may answer affirmatively the second fundamental question raised above since the singular points of the scalar curvature, Equation (32), are the same points that violate the Bendixson criterion. Consequently, one may write that our third example has a unique limit cycle. This result is in remarkable agreement with those found in our first and second examples.

To conclude, we have introduced and investigated three contrasting examples of dynamical systems with unique limit cycles. Despite the standard methods providing a reasonable approach to determine the non-existence of limit cycles, it becomes clear that one cannot extract conclusions about the existence or uniqueness of an isolated periodic trajectory only through the Bendixson–Dulac negative theorem. The existence of limit cycles in the three examples discussed above had only been inferred by these negative theorems and confirmed by the analysis of phase portraits. Moreover, the standard methods considered above either exclude or guarantee the existence of isolated closed trajectories in non-simply connected regions. Hence, we may write that different methods are required to confirm whether a dynamical system has a limit cycle from the viewpoint of the theorems of Bendixson, Dulac, and Poincaré.

Surprisingly, this is quite unlike in the framework of GBT. The analysis of the three examples reveals that the sign of R is positive and |R| diverges to infinity at symmetrical critical points. This result, of course, affirmatively answers the second fundamental question raised above and allows us to conclude that two-dimensional dissipative autonomous systems with a unique limit cycle are those in which the scalar curvature is positive and the magnitude of R diverges to infinity at symmetrical singular points with respect to the origin.

However, it may now be argued whether the validity of this last statement holds based on what we have discovered from the study of the dissipative Duffing oscillator [25]. In our initial paper, we investigated a Duffing oscillator in the framework of a three-dimensional Riemannian geometry. Hence, the family of probability distributions, Ω, was parameterized by three real parameters, $X=\left(X^{1},X^{2}\right)=\left(s_{1},s_{2},m\right)$, in which *m* is a control parameter. There, our results revealed that the scalar curvature is also positive, and its magnitude diverges to infinity.

The apparent contradiction between these results may be resolved by a more detailed analysis of the curvature R for the perturbed model of Duffing. To construct the Riemannian metrical structure of the perturbed Duffing oscillator in accordance with the geometrical methods derived in Section 2, we shall regard the control and perturbation parameters as fixed so that a Duffing oscillator may be investigated in the framework of a two-dimensional Riemannian geometry. Therefore, the equations

(50) $$\left\{\begin{array}{ccc} & \frac{ds_{1}}{d\tau } & =\Phi_{1}\left(s_{1},s_{2}\right)=s_{2},\\ & \frac{ds_{2}}{d\tau } & =\Phi_{2}\left(s_{1},s_{2}\right)=s_{1}-s_{1}^{3}-\frac{1}{3}s_{2}+s_{1}^{2}s_{2}, \end{array}\right.$$

now describe the mathematical model of the perturbed Duffing oscillator, in conformance with Ref. [25]. Here, we should note that this model has three equilibrium points: $p_{1}=\left(0,0\right)$ and $p_{2,3}=\left(\pm 1,0\right)$.

By employing the geometrical methods outlined above and Equations (5)–(8), one obtains the following curvature R for the perturbed oscillator:

(51) $$R=\frac{6\left(60s_{1}^{2}-81s_{1}^{4}-54s_{1}^{6}+81s_{1}^{8}+108s_{1}^{3}s_{2}-10\right)}{{\left(10-6s_{1}^{2}+9s_{1}^{4}\right)}^{2}{\left(1-3s_{1}^{2}+2s_{1}s_{2}\right)}^{3}},$$

in which one may recognize that R is a multivariable function expressed only in terms of the order parameters $s_{1}$ and $s_{2}$. Despite the curvature R, Equation (51), being different from that obtained in our initial paper, Equation (74) of Ref. [25], we shall demonstrate that the same conclusions obtained in Ref. [25] may also be inferred from the analysis of Equation (51).

Following the developments of the earlier sections, we now dedicate ourselves to the sign of curvature R.

To discuss the positivity of the scalar curvature, as defined by Equation (51), we must demonstrate that χ(M)>0. In a manner quite analogous to that presented for the previous examples, we first determine the nature of the equilibrium points of Equation (50) and their respective indices. Second, we dedicate ourselves to the computation of the Euler-characteristic number χ(M). Finally, the sign of the scalar curvature is interpreted with the aid of the positivity condition expressed by Equation (28).

As we have outlined above, Equation (43) possesses three different equilibrium points. Without entering into repetitious details, the analysis of the Jacobian matrix evaluated at these equilibrium points reveals that $p_{1}=\left(0,0\right)$ is a saddle, while $p_{2}=\left(1,0\right)$ and $p_{3}=\left(-1,0\right)$ designate sources. From the Poincaré–Hopf index theorem, we identify the Euler characteristic of M to be

(52) $$\chi \left(M\right)=\sum_{i=1}^{3}I_{i}\left(p_{i}\right)=I_{1}\left(p_{1}\right)+I_{2}\left(p_{2}\right)+I_{3}\left(p_{3}\right)=-1+1+1=1.$$

Since Equations (28) and (52) assure us that R>0, the necessary property of the positivity of the scalar curvature is demonstrated. With this result before us, along with the geometrical interpretation of the sign of R, we may write that the orbits of the phase space are periodic trajectories. This result agrees with those obtained for the perturbed Duffing oscillator in the framework of the three-dimensional metrical structure considered in our initial paper.

A further remark concerns the divergent behavior of the magnitude of R. Independently of the algebraic sign of $s_{1}$, it is not difficult to realize that |R| diverges to infinity at $s_{2}=0$. Remarkably, this is the same result found previously for the homoclinic bifurcations in Ref. [25]. Hence, we may write that the divergence behavior of the scalar curvature, Equation (51), marks the phenomena of homoclinic bifurcations.

Furthermore, a more careful analysis of Equation (51) reveals that |R| has an additional divergence to infinity at $p_{\pm }^{C}=\left(\pm 1,\pm 1\right)$. In so far as R>0 and |R| is singular at the two symmetrical singularities $p_{\pm }^{C}=\left(\pm 1,\pm 1\right)$, we thus are led to conclude that the perturbed Duffing oscillator has, indeed, a limit cycle from the standpoint of GBT and the results found for our last examples.

A useful check on this last conclusion is afforded by analyzing Equation (50) with the aid of the well-known concept of saddle quantity [89,90,91]. Accordingly, let $p_{0}$ be a saddle equilibrium point of the autonomous dynamical system of Equation (1) with two real eigenvalues of opposite signs $\Lambda_{1}$ and $\Lambda_{2}$. The relation

(53) $$Q=\Lambda_{1}+\Lambda_{2}$$

is called the saddle quantity or value of the system under consideration [89,90,91]. This relation states that a homoclinic bifurcation may result in the appearance of a stable limit cycle by virtue of the perturbation or variation of the control parameters of Equation (1). In particular, if the sum of the two real eigenvalues associated with $p_{0}$ is less than zero, that is, $\Lambda_{1}$ + $\Lambda_{2}<0$, this indicates that the homoclinic orbit is stable from the inside, and there is a birth of a unique limit cycle.

With this definition in mind, we turn back to the equilibrium points of the perturbed Duffing oscillator. From Equation (50), it is not difficult to realize that $p_{1}=\left(0,0\right)$ is a saddle equilibrium point in accordance with CBT. Correspondingly, the eigenvalues associated with $p_{1}=\left(0,0\right)$ are

(54) $$\Lambda_{1}=\frac{\left(-1-\sqrt{37}\right)}{6}\;\mathrm{and}\;\Lambda_{2}=\frac{\left(-1+\sqrt{37}\right)}{6}.$$

From which it naturally follows that

(55) $$Q=-\frac{1}{3}<0.$$

Hence, we may conclude that the homoclinic orbit bifurcation in the perturbed Duffing oscillator leads to the birth of a stable limit cycle [89,90,91].

This result agrees precisely with the conclusions found here from the viewpoint of the covariant formulation of GBT. As we have previously remarked, |R|→∞ at the homoclinic bifurcation point in the perturbed Duffing oscillator. However, just beyond this bifurcation point, the magnitude of R becomes singular again at the two symmetrical critical points $p_{\pm }^{C}=\left(\pm 1,\pm 1\right)$. This behavior indicates that the homoclinic trajectory disappears and is replaced by a limit cycle as a result of the large perturbations in the model of Duffing. Rigorously, this result teaches us that homoclinic bifurcations lead to the birth of limit cycles.

Furthermore, this last result demonstrates the validity of our geometrical approach, as it enforces that the dissipative dynamical systems with only one limit cycle are those in which their scalar curvature is positive and whose magnitude |R| diverges to infinity at symmetrical singular points with respect to the origin. Therefore, we may write that those conclusions affirmatively answer the second fundamental question raised above.

We now proceed to the final subsection, where we employ the geometrical methods of Section 2 to investigate dynamical systems with more than one limit cycle.

### 3.3. Systems with More than One Limit Cycle

In the previous subsection, we proved that if scalar curvature is positive and its magnitude diverges to infinity at symmetrical singularities with respect to the origin, then the dynamical system under consideration exhibits a unique limit cycle. With our aim of proving Hilbert’s sixteenth problem in mind, we must extend our conclusion in Section 3.2 to dynamical systems with more than one limit cycle.

Based on the above, we now dedicate ourselves to the class of two-dimensional dynamic systems with two limit cycles. Hence, we organize this subsection as follows. First, three contrasting examples with more than one limit cycle are introduced and investigated in the framework of Fisher information geometry. Here, we analyze the sign and magnitude of curvature R, closely following the program of Section 2. Finally, we construct a suitable covariant definition of limit cycles from the viewpoint of GBT based on the conclusions derived in these subsections.

#### 3.3.1. First Example

As a first example of a dynamical system with more than one limit cycle, we have [17]

(56) $$\begin{array}{c} \left\{\begin{array}{ccc} & \frac{ds_{1}}{d\tau } & =\Phi_{1}\left(s_{1},s_{2}\right)=-\frac{s_{1}}{2}-s_{1}^{2}-s_{2}+s_{1}s_{2}+s_{2}^{2},\\ & \frac{ds_{2}}{d\tau } & =\Phi_{2}\left(s_{1},s_{2}\right)=s_{1}\left(1+s_{1}-3s_{2}\right). \end{array}\right. \end{array}$$

It is not difficult to realize that this system has two equilibrium points $p_{1}=\left(0,0\right)$ and $p_{2}=\left(0,1\right)$. Let us now confirm the existence of limit cycles of Equation (56) from the standard methods presented above. From Bendixson’s criterion and Equation (56) it follows that

(57) $$\nabla \cdot \left(\Phi_{1}\left(s_{1},s_{2}\right),\Phi_{2}\left(s_{1},s_{2}\right)\right)=-\frac{1}{2}-5s_{1}+s_{2}.$$

Therefore, we are led to conclude that if a limit cycle exists, it must necessarily cross the curve $-1/2-5s_{1}+s_{2}=0$. However, the Bendixson criterion neither assures the existence of limit cycles nor the possible number of isolated periodic orbits of Equation (56). That is to say, we cannot extract substantial evidence about limit cycles for our first example only through the negative Bendixson theorem, and additional methods become necessary to provide a proper answer to the problem.

**Figure 9:**
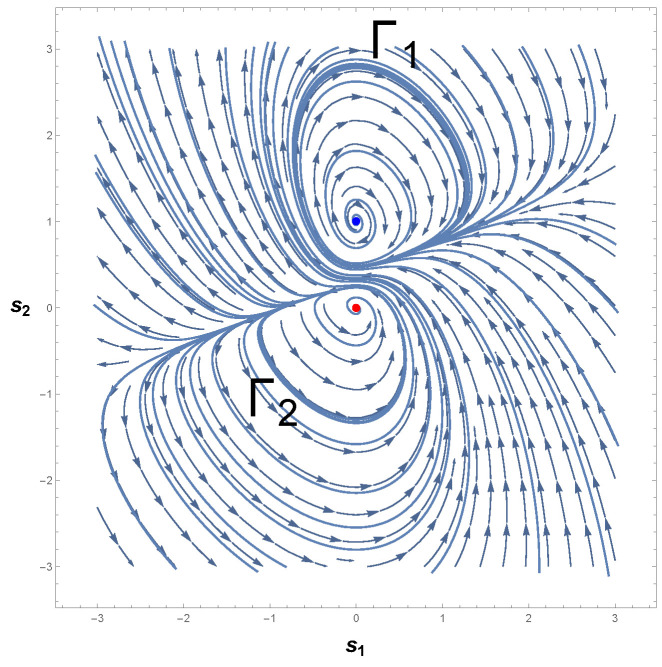
The phase portrait of Equation (56) in the neighborhood of its equilibrium points. Here, blue dots refer to unstable equilibrium points, while red dots represent stable points in accordance with CBT. As one may observe, there are two different limit cycles, which are indicated by $\Gamma_{1}$ and $\Gamma_{2}$. The first isolated periodic trajectory, $\Gamma_{1}$, involves the equilibrium point $p_{1}=\left(0,1\right)$ (blue dot), while $p_{2}=\left(0,0\right)$ (red dot) is found to be involved in the second isolated periodic orbit $\Gamma_{2}$. This is in complete agreement with the Poincaré–Hopf index theorem and allows one to conclude that the dissipative dynamical system, Equation (56), has more than one limit cycle.

This problem could be resolved by a careful analysis of the phase portrait of Equation (56). Figure 9 shows the behavior of Equation (22). As we may observe, the phase portrait confirms the existence of two limit cycles in the neighborhood of the equilibrium points $p_{1}=\left(0,0\right)$ and $p_{2}=\left(0,1\right)$. In addition, one may realize that the first isolated periodic trajectory, $\Gamma_{1}$, involves the equilibrium point $p_{1}=\left(0,1\right)$, while $\Gamma_{2}$ entails $p_{2}=\left(0,0\right)$. This result agrees with the Poincaré–Hopf index theorem. Consequently, we may see that the dynamical system, Equation (56), has two limit cycles.

Having examined the existence of limit cycles from the standard methods, let us turn our attention to the examination of limit cycles in the framework of GBT. We start from the covariant formulation of GBT outlined in Section 2. From Equations (56) and (5)–(7), we then obtain

(58) $$d\ell^{2}=2\left[{\left(1+2s_{1}-3s_{2}\right)}^{2}+{\left(s_{2}-\frac{1}{2}-2s_{1}\right)}^{2}\right]{\left(ds_{1}\right)}^{2}+2\left[9s_{1}^{2}+{\left(s_{1}-1+2s_{2}\right)}^{2}\right]{\left(ds_{2}\right)}^{2},$$

which corresponds to an invariant positive definite metric. Naturally, Equation (58) induces a curvature R on the two-dimensional parameter space *X* of Equation (56),

(59) $$\begin{array}{c} R=[92-4s_{1}\left(95+2s_{1}\left(27+8s_{1}\left(7+128s_{1}\right)\right)\right)+8\left(-87+2s_{1}\left(211+4s_{1}\left(1+56s_{1}\right)\right)\right)s_{2}\\ +96\left(19+s_{1}\left(16s_{1}-97\right)\right)s_{2}^{2}+896\left(9s_{1}-2\right)s_{2}^{3}+384s_{2}^{4}]B{\left(s_{1},s_{2}\right)}^{-1}, \end{array}$$

in which we have written

(60) $$B\left(s_{1},s_{2}\right)=\left[{\left(10s_{1}^{2}+{\left(1-2s_{2}\right)}^{2}+s_{1}\left(4s_{2}-2\right)\right)}^{2}{\left(5+32s_{1}^{2}+8s_{1}\left(3-8s_{2}\right)+4s_{2}\left(10s_{2}-7\right)\right)}^{2}\right].$$

From Equation (59), it is not difficult to verify that R is a multivariable function expressed in terms of the second and third derivatives of the nonlinear function $\Phi_{i}$ of Equation (56). With this preparation, we now investigate the trajectories of the phase space of Equation (34) through a general analysis of the sign and the magnitude of scalar curvature R.

The sign of Equation (59) follows from the condition Equation (28), expressed in terms of the Euler characteristic. Hence, we first need to determine the nature of the equilibrium points of Equation (56) and their respective indices. Second, the computation of the Euler-characteristic number χ(M) is made. Finally, we interpret the sign of scalar curvature through the positivity condition discussed above.

The analysis of the Jacobian matrix of Equation (56) evaluated at the equilibrium points reveals that $p_{1}=\left(0,0\right)$ is a sink, while $p_{2}=\left(0,1\right)$ designates a source. However, sources and sinks have the same index according to the Poincaré–Hopf theorem. Consequently, this permits the Euler characteristic of M to be

(61) $$\chi \left(M\right)=\sum_{i=1}^{2}I_{i}\left(p_{i}\right)=I_{1}\left(p_{1}\right)+I_{2}\left(p_{2}\right)=2.$$

**Figure 10:**
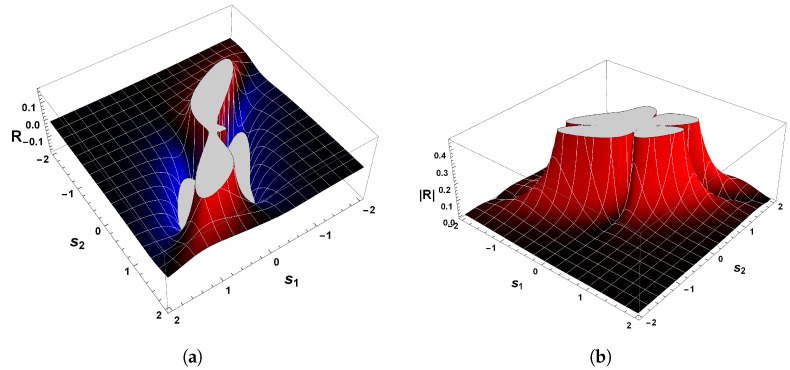
(**a**) shows the behavior of the scalar curvature R as a function of $s_{1}$ and $s_{2}$ for variations of ($s_{1}$, $s_{2}$) for our first example of Equation (56). Following the sign convention of Ref. [25], the red and blue colors represent positive and negative values of the curvature R, respectively. The black color refers to null values of R. As we may observe, the scalar curvature is positive in the neighborhood of its singular points, which teaches us that the phase portrait of Equation (56) has periodic trajectories. (**b**) depicts the magnitude of R. It is evident that |R| becomes singular at two different singularities: $p_{1}^{C}=\left(0,1/2\right)$ and $p_{2}^{C}=\left(-1/8,1/4\right)$.

Hence, we are led to conclude that the scalar curvature R is a positive multivariable function, as can be confirmed by Figure 10a, which shows the behavior of scalar curvature for our first example. Evaluations with Equation (59) show that the real curvature R is positive in the physical regime, which reveals that the state space of Equation (59) has periodic trajectories and it is in agreement with the analysis of the phase portrait, as illustrated in Figure 9.

In as much as the necessary condition expressed by Equations (28) guarantees the positivity of R, we may now proceed to the magnitude of scalar curvature. In order to determine the character of the orbits of the phase space and demonstrate the existence of limit cycles in our first example, a careful analysis of the magnitude of R is required. Hence, let us now direct our attention to the divergent behavior of |R| shown in Figure 10b. By employing geometrical methods identical to those of Refs. [25,26,82], we may realize that |R|→∞ when the denominator of Equation (59) vanishes. Consequently, it naturally follows that

(62) $$\left|R\right|\to \infty \;\mathrm{as}\;p_{1}^{C}\to \left(0,\frac{1}{2}\right).$$

This result basically teaches us that the magnitude of R diverges at a critical point $p_{1}^{C}=\left(0,1/2\right)$, which clearly does not follow the nature of the singular points determined in Section 3.2. With this lesson in mind, we must now return to our starting point and carefully re-examine Equation (57), in which the Bendixson criterion has been applied to Equation (56). Accordingly, we have demonstrated that if a limit cycle exists in a simply connected region, then this isolated periodic orbit must, necessarily, cross the curve $s_{2}-1/2-5s_{1}=0$, in which $\nabla \cdot \left(\Phi_{1}\left(s_{1},s_{2}\right),\Phi_{2}\left(s_{1},s_{2}\right)\right)=0$. Hence, comparing Equation (57) with Equation (62), we obtain

(63) $$\begin{array}{cc} & \left|R\right|\to \infty \;\mathrm{and}\;\nabla \cdot \left(\Phi_{1}\left(s_{1},s_{2}\right),\Phi_{2}\left(s_{1},s_{2}\right)\right)\to 0\\ & \mathrm{as}\;p_{1}^{C}\to \left(0,\frac{1}{2}\right). \end{array}$$

from which it is evident that the singular critical point $p_{1}^{C}=\left(0,1/2\right)$ of |R| is the same point that violates Bendixson’s criterion. This fact, together with the sign interpretation of R and |R|→∞, allows us to conclude that our first example of this subsection indeed has a limit cycle whose relative position corresponds to the singular critical point $p_{1}^{C}=\left(0,1/2\right)$ of the magnitude of Equation (59). This result is in complete agreement with the analysis of the phase portrait of Equation (56) and conclusions extracted from the theorem of Bendixson. Furthermore, it is not difficult to realize from the analysis of the phase portrait that this limit cycle is properly the isolated periodic trajectory denoted by $\Gamma_{1}$, as illustrated in Figure 9.

Nevertheless, a careful analysis of the denominator of Equation (59) clearly reveals that |R| has an additional divergence to infinity. That is,

(64) $$\left|R\right|\to \infty \;\mathrm{as}\;p_{2}^{C}\to \left(-\frac{1}{8},\frac{1}{4}\right).$$

This result, together with the sign interpretation of R and |R|→∞, allows us to realize that Equation (56) has a second limit cycle, whose relative position corresponds to the second singular critical point $p_{2}^{C}=\left(-1/8,1/4\right)$ of the magnitude of scalar curvature. Nevertheless, comparing Equation (57) with Equation (64), we realize that

(65) $$\begin{array}{cc} & \left|R\right|\to \infty \;\mathrm{and}\;\nabla \cdot \left(\Phi_{1}\left(s_{1},s_{2}\right),\Phi_{2}\left(s_{1},s_{2}\right)\right)\to \left(\frac{3}{8}\right)\\ & \mathrm{as}\;p_{2}^{C}\to \left(-\frac{1}{8},\frac{1}{4}\right). \end{array}$$

This result basically tells us that the second critical point of |R| does not violate the Bendixson theorem. On account of the fact that $\nabla \cdot \left(\Phi_{1}\left(s_{1},s_{2}\right),\Phi_{2}\left(s_{1},s_{2}\right)\right)$ does not change sign, and it is not zero, we then would be led to conclude that there are no limit cycles in the neighborhood of $p_{2}^{C}=\left(-1/8,1/4\right)$. However, this apparent contradiction is resolved by recalling that the Bendixson and Dulac theorems only allow one to infer the existence of limit cycles in simply connected regions. Nevertheless, these neither exclude, guarantee, or assure isolated closed trajectories in non-simply connected domains.

Therefore, we may conclude that our first example of this subsection, Equation (56), presents an additional limit cycle whose relative position is determined by the second critical point of the magnitude of scalar curvature R, precisely, $p_{2}^{C}=\left(-1/8,1/4\right)$, which relies upon a non-simply connected region of the phase space of Equation (56). As a check upon this result, one may verify that this second limit cycle corresponds to the isolated periodic trajectory denoted by $\Gamma_{2}$, as shown in Figure 9.

These results, which are in remarkable agreement with the analysis of the phase portrait of Equation (56), confirm that our first example has two limit cycles from the standpoint of GBT. Also, we may write that the conclusions obtained through the geometrical methods of Section 2 affirmatively answer the second fundamental question raised above. In addition, it suggests that two-dimensional dissipative dynamic systems with more than one limit cycle are those in which their scalar curvature is positive and the magnitude of R diverges to infinity at distinct points. More precisely, critical points not symmetrical with respect to the origin. Alternatively, we may write that if R>0 and |R|→∞ at distinct points, then the total number of divergences of |R| to infinity is the total number of limit cycles that a dissipative dynamical system may exhibit in its phase portrait.

For the validity of this last statement, we provide additional evidence concerning the existence of more than one limit cycle in dissipative systems through the framework of GBT. Hence, let us now look at two more examples to enforce the conclusions derived above.

#### 3.3.2. Second Example

As a second elementary example of a dynamical system with more than one limit cycle, we have [92]

(66) $$\begin{array}{c} \left\{\begin{array}{ccc} & \frac{ds_{1}}{d\tau } & =\Phi_{1}\left(s_{1},s_{2}\right)=-s_{2}-3s_{1}^{2}+s_{1}s_{2}+s_{2}^{2},\\ & \frac{ds_{2}}{d\tau } & =\Phi_{2}\left(s_{1},s_{2}\right)=s_{1}\left(1+\frac{s_{1}}{6}-3s_{2}\right). \end{array}\right. \end{array}$$

From this, it naturally follows that our second example has two equilibrium points: $p_{1}=\left(0,0\right)$ and $p_{2}=\left(0,1\right)$. Let us now confirm the existence of limit cycles of Equation (66) from the usual standard methods presented above. From Bendixson’s criterion and Equation (66), we see that

(67) $$\nabla \cdot \left(\Phi_{1}\left(s_{1},s_{2}\right),\Phi_{2}\left(s_{1},s_{2}\right)\right)=-9s_{1}+s_{2}.$$

Hence, we may conclude that if Equation (66) has an isolated periodic trajectory in a simply connected region, then this isolated periodic orbit must necessarily cross the curve $-9s_{1}+s_{2}=0$. Again, it does not seem possible to confirm whether or not this second example has limit cycles from Bendixson’s criterion.

Unfortunately, it does not seem possible to confirm whether or not our second example has one or more isolated periodic trajectories, since the negative Bendixson theorem alone neither assures the existence of limit cycles nor the possible number of limit cycles of Equation (66).

**Figure 11:**
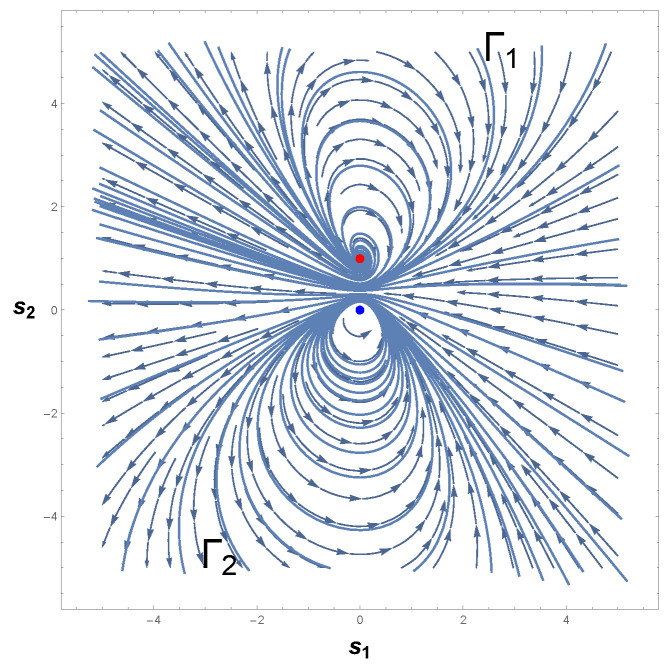
The phase portrait of Equation (66) in the neighborhood of its equilibrium points $p_{1}=\left(0,0\right)$ and $p_{2}=\left(0,1\right)$. Here, blue dots refer to unstable equilibrium points, while red dots represent stable points in accordance with CBT. As one may observe, there are two different limit cycles, which are indicated by $\Gamma_{1}$ and $\Gamma_{2}$. The first limit cycle, $\Gamma_{1}$, involves the equilibrium point $p_{1}=\left(0,1\right)$ (red dot), while $p_{2}=\left(0,0\right)$ (blue dot) is found to be involved in the second limit cycle $\Gamma_{2}$. This agrees with the Poincaré–Hopf index theorem and allows us to conclude that the our second example, Equation (66), has more than one limit cycle.

This problem, again, can be resolved by a careful analysis of the phase portrait of Equation (66). Figure 11 shows the behavior of our second example of this subsection. As one can readily see, the phase portrait confirms the existence of two limit cycles: $\Gamma_{1}$ and $\Gamma_{2}$. In particular, we observe that the first isolated periodic trajectory, $\Gamma_{1}$, entails the equilibrium point $p_{1}=\left(0,1\right)$, while $\Gamma_{2}$ involves $p_{2}=\left(0,0\right)$. This result agrees with that obtained by Zhang [92], who demonstrated that there is at least one limit cycle surrounding each of $p_{1}=\left(0,0\right)$ and $p_{2}=\left(0,1\right)$.

Furthermore, the behavior of Equation (66) is in accordance with the Poincaré–Hopf index theorem, as an isolated periodic trajectory of the phase space must contain at least one equilibrium point. Thus, we may infer that the dynamical system, Equation (66), indeed has two limit cycles.

Having investigated the existence of limit cycles from the well-known standard methods, we now turn our attention to the examination of limit cycles in the framework of information geometry. With the aid of Equations (5)–(7) and (66), we see that

(68) $$\begin{array}{c} d\ell^{2}=2\left[{\left(1+\frac{s_{1}}{3}-3s_{2}\right)}^{2}+{\left(s_{2}-6s_{1}\right)}^{2}\right]{\left(ds_{1}\right)}^{2}+2\left[9s_{1}^{2}+{\left(s_{1}+2s_{2}-1\right)}^{2}\right]{\left(ds_{2}\right)}^{2}, \end{array}$$

corresponds to an invariant positive definite metric.

Hence, the Fisher metric, Equation (68), will induce the following curvature R on the two-dimensional parameter space *X* of Equation (66):

(69) $$\begin{array}{c} R=C{\left(s_{1},s_{2}\right)}^{-2}[9\left(140s_{1}^{4}-8s_{1}^{3}\left(141s_{2}+233\right)+s_{1}^{2}\left(2s_{2}\left(11-816s_{2}\right)+778\right)\right)\\ +9\left(s_{1}\left(4s_{2}\left(s_{2}\left(412s_{2}-303\right)+90\right)-101\right)+12s_{2}^{2}\left(s_{2}\left(12s_{2}-77\right)+75\right)-315s_{2}+39\right)], \end{array}$$

whence,

(70) $$C\left(s_{1},s_{2}\right)=\left[\left(325s_{1}^{2}-18\left(7s_{1}+3\right)s_{2}+6s_{1}+90s_{2}^{2}+9\right)\left(10s_{1}^{2}+s_{1}\left(4s_{2}-2\right)+{\left(1-2s_{2}\right)}^{2}\right)\right].$$

One should note that the scalar curvature is a real multivariable function. From the knowledge of R, we now investigate the trajectories of the phase space of Equation (66) through a general analysis of the sign and the magnitude of the scalar curvature R. To discuss the positivity of Equation (69), it is a simple matter to carry out the program previously mentioned and demonstrate that χ(M)>0. It should be noted that Equation (69) has two equilibrium points. The analysis of the Jacobian matrix of Equation (66) reveals that $p_{1}=\left(0,0\right)$ is a center, while $p_{2}=\left(0,1\right)$ is a focus. From the Poincaré index theorem, we may identify the index $I(p_{i})$ or the Euler characteristic of M to be

(71) $$\chi \left(M\right)=\sum_{i=1}^{2}I_{i}\left(p_{i}\right)=I_{1}\left(p_{1}\right)+I_{2}\left(p_{2}\right)=2.$$

**Figure 12:**
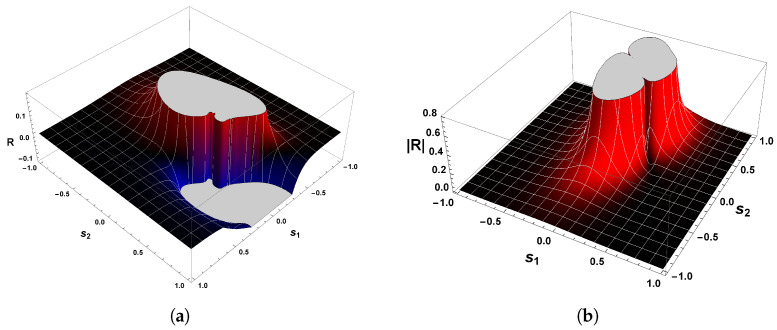
(**a**) shows the behavior of the scalar curvature as a function of $s_{1}$ and $s_{2}$ for variations in ($s_{1}$, $s_{2}$) for our second example of Equation (66). Following the sign convention of Ref. [25], the red and blue colors represent positive and negative values of the curvature R, respectively. The black color refers to null values of R. As we may observe, the scalar curvature is positive in the neighborhood of its singular points, which teaches us that the phase portrait of Equation (66) has periodic trajectories. (**b**) depicts the magnitude of R. It is evident that |R| becomes singular at two different critical points $p_{1}^{C}=\left(0,0\right)$ and $p_{2}^{C}=\left(0,1\right)$.

Since χ(M)>0, then we have thereby demonstrated the positiveness of the scalar curvature. Figure 12a shows the behavior of R for our second example. Evaluations with Equation (69) reveal that the curvature R is real and positive in the physical regime. From the geometrical interpretation of the sign of curvature R, we thus may conclude that the state space of Equation (66) has periodic trajectories, which is in agreement with the analysis of the phase portrait, as shown in Figure 11.

In order to exploit the character of the orbits of the phase space and demonstrate the existence of limit cycles for Equation (66), a careful analysis of the divergent behavior of |R| is also required. By employing the geometrical methods identical to those of Refs. [25,26,82], we conclude that

(72) $$\left|R\right|\to \infty \;\mathrm{as}\;p_{1}^{C}\to \left(0,\frac{1}{2}\right)$$

and

(73) $$\left|R\right|\to \infty \;\mathrm{as}\;p_{2}^{C}\to \left(\frac{3}{53},\frac{18}{53}\right).$$

The result expressed by Equations (72) and (73) reveals that the magnitude of R has two critical point divergences at $p_{1}^{C}=\left(0,1/2\right)$ and $p_{2}^{C}=\left(3/53,18/53\right)$, as illustrated in Figure 12b. Therefore, it is evident, of course, that this result does not follow the nature of the singular points determined in Section 3.2. That is to say, the magnitude of R has two distinct singularities, in which neither of them are symmetrical with respect to the origin.

Bearing this in mind, we now return to Equation (67). As discussed above, if a limit cycle exists in a simply connected region, then this isolated periodic orbit must, necessarily, cross the curve $-9s_{1}+s_{2}=0$, in which $\nabla \cdot \left(\Phi_{1}\left(s_{1},s_{2}\right),\Phi_{2}\left(s_{1},s_{2}\right)\right)$ becomes zero. However, on proceeding in a manner parallel to the corresponding discussion in our first example, of a dynamical system with two limit cycles, we see, after a comparison with Equations (67) and (72), that neither $p_{1}^{C}=\left(0,1/2\right)$ nor $p_{2}^{C}=\left(3/53,18/53\right)$ violate the Bendixson theorem.

On account of the fact that $\nabla \cdot \left(\Phi_{1}\left(s_{1},s_{2}\right),\Phi_{2}\left(s_{1},s_{2}\right)\right)$ does not change sign, and it is not zero, we then would be led to conclude that there are no limit cycles in the neighborhood of either $p_{1}^{C}=\left(0,1/2\right)$ or $p_{2}^{C}=\left(3/53,18/53\right)$. Nonetheless, as we have discussed previously, the contradiction between the results found from the analysis of R for Equation (66) along with the analysis of the phase portrait of Equation (66) and the negative theorem of Bendixson is resolved again by recalling that these theorems only allow one to infer the existence of limit cycles in simply connected regions. These neither exclude nor guarantee the existence of isolated closed trajectories in non-simply connected domains.

Under this interpretation, we thus conclude that the second example of this subsection, Equation (66), presents two limit cycles whose relative positions are determined by the two singularities of |R|, which lie on a non-simply connected region of the phase space of Equation (66). The first limit cycle corresponds to the isolated periodic trajectory denoted by $\Gamma_{1}$, where it is found in the vicinity of $p_{1}^{C}=\left(0,1/2\right)$. The second limit cycle corresponds to the isolated periodic trajectory denoted by $\Gamma_{2}$, where one may find it in the neighborhood of $p_{2}^{C}=\left(3/53,18/53\right)$. These results, which are in complete agreement with the conclusions found by Zhang and the analysis of the phase portrait of Equation (66), once again demonstrate the validity of our approach, because the study of curvature R confirms that Equation (66) has two limit cycles from the standpoint of GBT.

#### 3.3.3. Final Example

As a final example, we consider the following system of differential equations [4]:

(74) $$\begin{array}{c} \left\{\begin{array}{ccc} & \frac{ds_{1}}{d\tau } & =\Phi_{1}\left(s_{1},s_{2}\right)=-s_{2}-s_{2}^{2},\\ & \frac{ds_{2}}{d\tau } & =\Phi_{2}\left(s_{1},s_{2}\right)=s_{1}s_{2}+\left(\frac{s_{1}}{2}\right)-\left(\frac{s_{2}}{5}\right)-\left(\frac{6s_{2}^{2}}{5}\right), \end{array}\right. \end{array}$$

in which $p_{1}=\left(0,0\right)$ and $p_{2}=\left(-2,-1\right)$ are equilibrium points. We begin by employing the negative theorem of Bendixson presented earlier to confirm whether Equation (74) has limit cycles. Hence, we conclude from the Bendixson criterion and Equation (74) that

(75) $$\nabla \cdot \left(\Phi_{1}(s_{1},s_{2},\Phi_{2}\left(s_{1},s_{2}\right)\right)=s_{1}-\frac{1}{5}-\frac{12s_{2}}{5}.$$

That is, if our Equation (74) here exhibits a limit cycle, then this isolated periodic orbit must cross the curve $-1/5+s_{1}-12s_{2}/5=0$. However, one may see again that the Bendixson criterion provides no substantial evidence about the existence or a possible number of limit cycles for our third example. Consequently, more methods become necessary to appropriately answer this problem; notably, the analysis of the phase portrait of Equation (74).

**Figure 13:**
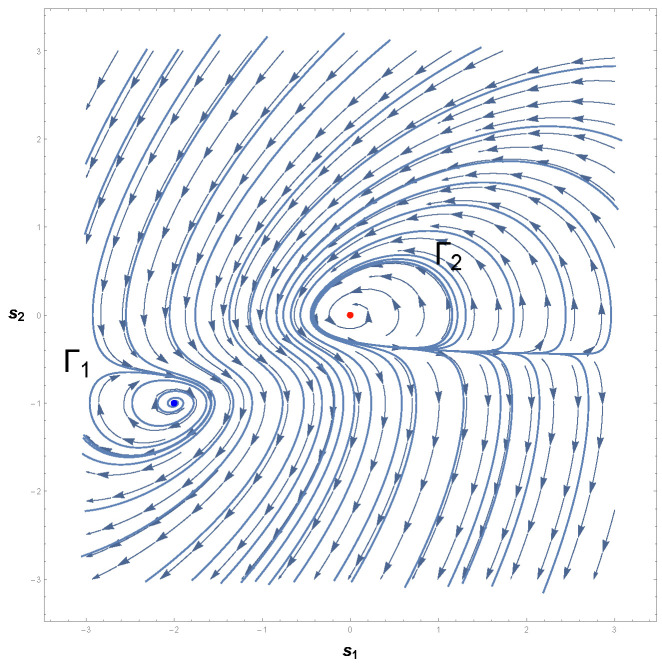
The phase portrait of Equation (74) in the neighborhood of the equilibrium points $p_{1}\left(s_{1}^{*},s_{2}^{*}\right)$ and $p_{2}\left(s_{1}^{*},s_{2}^{*}\right)$. Here, red dots represent stable points. Conversely, blue dots refer to unstable equilibrium points. As one may see, two different limit cycles are present in the phase portrait. Those isolated periodic trajectories are indicated by $\Gamma_{1}$ and $\Gamma_{2}$, respectively. The first isolated periodic trajectory, $\Gamma_{1}$, involves the equilibrium point $p_{1}\left(s_{1}^{*},s_{2}^{*}\right)=\left(-2,-1\right)$ (blue dot), while $p_{2}\left(s_{1}^{*},s_{2}^{*}\right)=\left(0,0\right)$ (red dot) is involved in the second isolated periodic orbit $\Gamma_{2}$. The phenomena shown in this figure are in complete agreement with the index theorem and allow one to infer that the dissipative dynamical system, Equation (74), has two limit cycles.

Figure 13 shows the behavior of Equation (74). As one may observe, the phase portrait confirms the existence of two limit cycles in the neighborhood of $p_{1}=\left(0,0\right)$ (blue dot) and $p_{2}=\left(-2,-1\right)$ (blue dot). Furthermore, one may realize that the first isolated periodic trajectory, $\Gamma_{1}$, entails the equilibrium point $p_{1}=\left(-2,-1\right)$, while $\Gamma_{2}$ involves $p_{2}=\left(0,0\right)$. This result agrees with that obtained by Chin Chu, who demonstrated the existence of these limit cycles by employing the standard methods outlined above [4]. Furthermore, the behavior of Equation (74) is in accordance with the Poincaré–Hopf index theorem. Consequently, we may infer that the dynamical system, Equation (74), has two limit cycles.

Having investigated the limit cycles of Equation (74) from the standpoint of standard methods, we now devote ourselves to the study of limit cycles in the framework of information geometry. With the aid of Equations (5)–(7) and (74), we see that

(76) $$d\ell^{2}=2{\left[\frac{1}{2}+s_{2}\right]}^{2}{\left(ds_{1}\right)}^{2}+2\left[{\left(-1-2s_{2}\right)}^{2}+{\left(s_{1}-\frac{1}{5}-\frac{12s_{2}}{5}\right)}^{2}\right]{\left(ds_{2}\right)}^{2},$$

corresponds to an invariant positive definite metric. As a result, the Fisher metric, Equation (76), imposes the following curvature R on the two-dimensional parameter space *X* of Equation (74):

(77) $$R=E{\left(s_{1},s_{2}\right)}^{-1}\left[600\left(5s_{1}-12s_{2}-1\right)\right],$$

where

(78) $$E\left(s_{1},s_{2}\right)=\left[2\left(\frac{1}{2}+s_{2}\right){\left(25{\left(s_{1}+1\right)}^{2}-120\left(s_{1}+1\right)\left(\frac{1}{2}+s_{2}\right)+244{\left(\frac{1}{2}+s_{2}\right)}^{2}\right)}^{2}\;\right].$$

One should note that the scalar curvature is a real multivariable function. From the knowledge of R, we now investigate the trajectories of the phase space of Equation (74) through a general analysis of the sign and the magnitude of scalar curvature R. It should be noted that Equation (74) has two equilibrium points $p_{1}=\left(0,0\right)$ and $p_{2}=\left(-2,-1\right)$.

The analysis of the Jacobian matrix of Equation (74) teaches us that the phase portrait associated with Equation (74) has two sources as equilibrium points. From Poincaré index theorem, we may identify the index $I(p_{i})$ or the Euler characteristic of M to be

(79) χ(M)=2.

**Figure 14:**
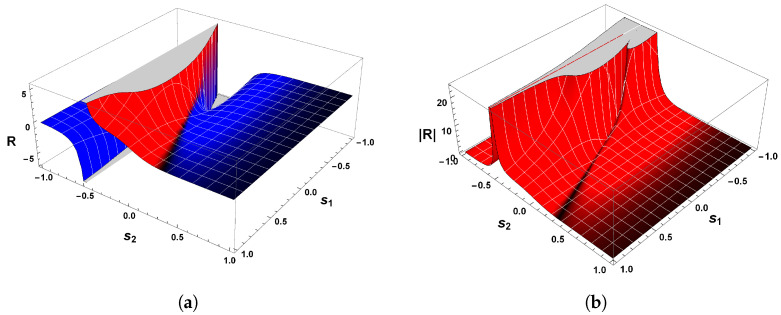
(**a**) The behavior of the scalar curvature as a function of $s_{1}$ and $s_{2}$ for variations in ($s_{1}$, $s_{2}$) for our first example of Equation (74). Following the sign convention of Ref. [25], the red and blue colors represent positive and negative values of the curvature R, respectively. The black color refers to null values of R. As we may observe, the scalar curvature is positive in the neighborhood of its singular points, which teaches us that the phase portrait of Equation (74) has periodic trajectories. (**b**) depicts the magnitude of R. It is evident that |R| becomes singular at $p^{C}=\left(-1,-1/2\right)$ and along $s_{2}=0$.

In as much as χ(M)>0, the defining property of the Euler characteristic guarantees the positiveness of Equation (77). That is, Equations (28) and (52) confirm the positivity of the scalar curvature R of our third example. Figure 14a shows the behavior of R for Equation (74). Evaluations with Equation (77) reveal that the scalar curvature is real and positive in the physical regime. From the geometrical interpretation of the sign of curvature R, we thus may conclude that the state space of Equation (74) has periodic trajectories, which is in agreement with the analysis of the phase portrait, as shown in Figure 13.

With the positiveness of R in mind, we may then exploit the character of the trajectories present in the phase space and provide the maximum number of limit cycles for Equation (74). To that end, we now dedicate ourselves to the study of the divergent behavior of |R|. By employing geometrical methods identical to those in Refs. [25,26,82], it follows from Equation (77) that the magnitude of scalar curvature diverges to infinity when $E(s_{1},s_{2})=0$.

One should notice, however, that Equation (78) is essentially a product of non-constant polynomial factors. Therefore, this naturally implies that the explicit solutions of $E(s_{1},s_{2})=0$ are simply the roots of those factors, which when solved yield

(80) $$s_{2}=-\frac{1}{2},\;p^{C}=\left(-1,-\frac{1}{2}\right).$$

which implies that $E(s_{1},s_{2})=0$, and consequently, that the magnitude of R diverges to infinity. More specifically,

(81) $$\left|R\right|\to \infty \;\mathrm{as}\;p^{C}\to \left(-1,-\frac{1}{2}\right)$$

and

(82) $$\left|R\right|\to \infty \;\mathrm{as}\;s_{2}\to -\frac{1}{2}.$$

In summary, we have found that the magnitude of |R| diverges to infinity at $s_{2}=-1/2$ and $p^{C}=\left(-1,-1/2\right)$, as illustrated in Figure 14b. Further, it is evident from Equation (80) that the maximum number of two singularities leads to the divergence of the magnitude of scalar curvature to infinity. The consistency of Equation (80) is confirmed by verifying that |R|→∞ at $s_{2}=-1/2$ and $p^{C}=\left(-1,-1/2\right)$.

Based on the above, let us return to the second fundamental question raised above. On account of the fact that the scalar curvature is positive and the magnitude of R diverges to infinity at two singularities, we thus may conclude that Equation (74) has exactly two different limit cycles from the standpoint of GBT. This latter fact provides an affirmative answer to the second fundamental problem, and consequently, shows the validity of GBT, since the result found for our third example is in complete agreement with the conclusion found by Chin Chu and the analysis of the phase portrait of Equation (74), as shown in Figure 13.

To conclude this subsection, we have investigated three contrasting mathematical models with two limit cycles, which led to the same geometrical conclusions found for the earlier examples of Section 3.3. These results enforce the interpretation in which two-dimensional dissipative systems with more than one limit cycle are those in which their scalar curvature is positive and |R| diverges to infinity at singularities not symmetrical to the origin, so that the total number of divergences of |R| to infinity provides the total number of limit cycles that a dissipative dynamical system may exhibit in its phase portrait. From the standpoint of the well-known standard methods, one cannot extract any substantial evidence of isolated periodic trajectories for any of the dynamical systems studied here.

Armed with the results found in Section 3.1, Section 3.2 and Section 3.3, we may now covariantly define limit cycles from the standpoint of GBT.

**Definition** **1.** *Let*

(83) $$\left\{\begin{array}{ccc} & \beta_{1} & =\frac{ds_{1}}{d\tau }=\Phi_{1}\left(s_{1},s_{2}\right),\\ & \beta_{2} & =\frac{ds_{2}}{d\tau }=\Phi_{2}\left(s_{1},s_{2}\right), \end{array}\right.$$

*represent the two-dimensional mathematical model of autonomous dynamical systems, where $\beta_{1}$ and $\beta_{2}$ are the momenta. Here, $s_{1}$ and $s_{2}$ designate the order parameters, while τ is time. Further, $\Phi_{1}$ and $\Phi_{2}$ denote the nonlinear functions of Equation (83). In the framework of GBT, a limit cycle is the periodic state of the dynamical system under study in which its correspondent scalar curvature is positive in the neighborhood of the equilibrium points $p_{i}$ of Equation (83) and |R| is singular. Precisely, if the scalar curvature is positive in the vicinity of $p_{i}$ and the magnitude of R diverges to infinity at symmetrical singularities with respect to the origin, then Equation (83) possesses only one limit cycle. Nonetheless, if the curvature *R* is positive in the neighborhood of the equilibrium points $p_{i}$ of Equation (83) and the magnitude of *R* diverges to infinity at different singularities, the present dynamical system under consideration has more than one limit cycle such that the total number of distinctive divergences of |R| to infinity provides the maximum number of limit cycles of Equation (83), with the relative positions of those isolated periodic trajectories being determined by the singularities of the magnitude of scalar curvature. More specifically, the singularities of the magnitude of *R* provide the relative positions of the limit cycles with respect to the equilibrium points involved in these isolated periodic trajectories. Finally, if R≤0, then Equation (83) presents no limit cycles.*

As one may realize, the definition above agrees with the definition of limit cycles in the framework of CBT as a limit cycle may also be understood as a periodic state; see Refs. [93,94,95]. In addition, from the standpoint of what is already known and consolidated from differential geometry, the existence of periodic trajectories is assured by the geometrical interpretation of the sign of R and the nature of the dynamical system. If R>0 and the dynamical system is dissipative, then the system undoubtedly possesses periodic trajectories, specifically limit cycles. This is because a limit cycle and a periodic solution cannot coexist within the same system [49,96].

As a result of all this, it is evident that our formulation expands the state of the art of nonlinear dynamics since GBT introduces an alternative way to understand and explore limit cycles in the framework of differential equations.

In the next section, we shall see the advantage of adopting this definition to successfully solve the second part of the sixteenth problem of Hilbert.

As a final remark, we must observe that if the dynamical system under consideration has two or more varying parameters and only one or two of them are analyzed, and consequently, R≤0, then one should resist the impulse to conclude that there are no limit cycles, since a careful analysis of the whole parameter space is required in order to obtain a more accurate conclusion about the existence of isolated periodic trajectories.

## 4. The Sixteenth Problem of Hilbert

In the previous section, we employed the covariant formulation of GBT to the elementary problem of isolated periodic trajectories of two-dimensional systems described by differential equations. Consequently, the concept of limit cycles had been covariantly defined.

The definition of limit cycles and the results expressed in Section 3 confirmed that GBT undoubtedly accounts for the maximum number and positions of the limit cycles of dynamical systems described by differential equations. With all this before us, we may now profitably return to the research problem raised above and devote ourselves to the second part of the sixteenth problem of Hilbert, closely following the geometrical methods of Section 2 and the developments of Section 3.

As we have already noted, Hilbert’s sixteenth problem is the quest for the maximum number and locations of limit cycles of planar polynomial systems of degree *n*. From a modern perspective, and taking into account practical applications, the second part of the sixteenth problem of Hilbert can be stated as follows [6,97,98,99,100]:

What is the maximum number and position of Poincaré limit cycles (isolated periodic trajectories) for the differential equation

(84) $$\frac{ds_{2}}{ds_{1}}=\frac{Q_{n}\left(s_{1},s_{2}\right)}{P_{n}\left(s_{1},s_{2}\right)}=\frac{\sum_{i,j\;\geq 0}^{n}b_{i\;j}{\left(s_{1}\right)}^{i}{\left(s_{2}\right)}^{j}}{\sum_{i,j\;\geq 0}^{n}a_{i\;j}{\left(s_{1}\right)}^{i}{\left(s_{2}\right)}^{j}},$$

in which $P_{n}\left(s_{1},s_{2}\right)$ and $Q_{n}\left(s_{1},s_{2}\right)$ are polynomials of nth degree with real coefficients **a** and **b**.

However, a more useful and convenient representation of Equation (84), and the one we actually employ for the calculations, is obtained by writing Equation (84) as

(85) $$\left\{\begin{array}{c} \frac{ds_{1}}{d\tau }=P_{n}\left(s_{1},s_{2}\right)=\sum_{j=0}^{n}\sum_{i=0}^{j}a_{i\;j-i}{\left(s_{1}\right)}^{i}{\left(s_{2}\right)}^{j-i},\\ \frac{ds_{2}}{d\tau }=Q_{n}\left(s_{1},s_{2}\right)=\sum_{j=0}^{n}\sum_{i=0}^{j}b_{i\;j-i}{\left(s_{1}\right)}^{i}{\left(s_{2}\right)}^{j-i}, \end{array}\right.$$

where, here and in the following, we take both $a_{00}$ and $b_{00}$ to be zero. Otherwise, Equation (85) would not possess equilibrium points.

As a natural consequence of the developments of Section 2 and Section 3, along with this modern viewpoint, we then realize that the problem of finding the maximum number and relative positions of limit cycles of Equation (85) is therefore equivalent to determining the total number of distinct divergences to infinity of the magnitude of the scalar curvature associated with Equation (85).

Therefore, from Equation (85) and proceeding in a manner similar to that of Section 3, we demonstrate that the covariant formulation of GBT directs us to a rigorous and explicit solution for the second part of Hilbert’s sixteenth problem.

In light of the foregoing, we organize this section as follows. First, we concern ourselves with second-degree polynomial systems. Here, a rigorous and explicit solution is obtained for the open problem of the total number and positions of limit cycles for quadratic polynomial systems. Finally, we aim at the general problem of *n*th-degree polynomial systems.

### 4.1. Second-Degree Polynomial Systems

We now devote ourselves to the problem of the maximum number and relative positions of limit cycles for quadratic polynomial systems. Much research has been carried out to solve this problem from the viewpoint of CBT, but so far, to no avail. Worth mentioning is the theorem of S. L. Shi derived in 1979 and its later generalization by Leonov and Kuznetsova in 2008; see Refs. [101,102,103].

The results of these and other previous investigations suggest that the maximum number of limit cycles for two-dimensional polynomial quadratic systems is equal to four [22,97,102,103]. But is it true? An affirmative answer is usually conventional, but the proper mathematical development of the problem with the treatment of GBT will allow us to state a definitive answer. To that end, we shall solve the following problem:

**Problem** **1.** *What is the maximum number and position of Poincaré limit cycles (isolated periodic trajectories) for the differential equation*

(86) $$\left\{\begin{array}{c} \frac{ds_{1}}{d\tau }=P_{2}\left(s_{1},s_{2}\right)=\sum_{j=0}^{2}\sum_{i=0}^{j}a_{i\;j-i}{\left(s_{1}\right)}^{i}{\left(s_{2}\right)}^{j-i},\\ \frac{ds_{2}}{d\tau }=Q_{2}\left(s_{1},s_{2}\right)=\sum_{j=0}^{2}\sum_{i=0}^{j}b_{i\;j-i}{\left(s_{1}\right)}^{i}{\left(s_{2}\right)}^{j-i}, \end{array}\right.$$

*or equivalently,*

(87) $$\begin{array}{c} \left\{\begin{array}{cc} & \frac{ds_{1}}{d\tau }=s_{2}a_{01}+s_{2}^{2}a_{02}+s_{1}a_{10}+s_{1}s_{2}a_{11}+s_{1}^{2}a_{20},\\ & \frac{ds_{2}}{d\tau }=s_{2}b_{01}+s_{2}^{2}b_{02}+s_{1}b_{10}+s_{1}s_{2}b_{11}+s_{1}^{2}b_{20}, \end{array}\right. \end{array}$$

where we take both $a_{00}$ and $b_{00}$ to be zero. Otherwise, Equation (85) would not possess equilibrium points.

We henceforth denote this the *two-dimensional version of Hilbert’s sixteenth challenge*. The general solution of this latter problem is provided by demonstrating the following theorem:

**Theorem** **3.** *In the framework of GBT, if the dynamical system under consideration is described by a real polynomial system of degree two with no common linear factors, Equation (87), then it has the maximum number of four limit cycles, and the relative positions of these isolated periodic trajectories are to be determined by the singular points of the magnitude of the scalar curvature for the polynomial of the second order.*

**Proof.**  To verify the statements of the problem as well as demonstrate that quadratic polynomial systems have the maximum number of four isolated periodic trajectories, we must show that the expected result is consistent with the covariant definition of limit cycles. Hence, we must prove that the corresponding scalar curvature of Equation (87) is positive, and the magnitude of R has four singularities.

Based on the above, this proof is organized as follows. First, we derive the corresponding Riemannian metric of Equation (87) from the geometrical methods of GBT and Equations (5)–(7). Second, we devote ourselves to the computation of the scalar curvature of Equation (87). Third, we dedicate ourselves to the sign interpretation of R to determine the character of the trajectories of the state space. Finally, we shall confine our attention to the singularities of the magnitude of curvature R by employing the methods developed in the preceding pages. Following the program of Section 2 and Section 3, the corresponding metric of Equation (87) is written as follows:

(88) $$\begin{array}{c} d\ell^{2}=2\left[{\left(a_{10}+s_{2}a_{11}+2s_{1}a_{20}\right)}^{2}+{\left(b_{10}+s_{2}b_{11}+2s_{1}b_{20}\right)}^{2}\right]{\left(ds_{1}\right)}^{2}\\ +2\left[{\left(a_{01}+2s_{2}a_{02}+s_{1}a_{11}\right)}^{2}+{\left(b_{01}+2s_{2}b_{02}+s_{1}b_{11}\right)}^{2}\right]{\left(ds_{2}\right)}^{2}, \end{array}$$

which is a positive definite and geometrically invariant Riemannian metric. The next stage is reached by the computation of the scalar curvature associated with Equation (88). Accordingly, the Riemannian metric, Equation (88), imposes a curvature R on the two-dimensional manifold of the parameter space of the quadratic system considered here. After some straightforward algebraic manipulations, the corresponding scalar curvature of Equation (87) is expressed by

(89) $$\begin{array}{cc} R & =[(\left(2{\left(2s_{1}a_{20}+s_{2}a_{11}+a_{10}\right)}^{2}+{\left(2s_{1}b_{20}+s_{2}b_{11}+b_{10}\right)}^{2}\right){\left(4a_{11}\left(s_{1}a_{11}+2s_{2}a_{02}+a_{01}\right)+2b_{11}\left(s_{1}b_{11}+2s_{2}b_{02}+b_{01}\right)\right)}^{2}\\ & +\left(2{\left(s_{1}a_{11}+2s_{2}a_{02}+a_{01}\right)}^{2}+{\left(s_{1}b_{11}+2s_{2}b_{02}+b_{01}\right)}^{2}\right)\left(8a_{20}\left(2s_{1}a_{20}+s_{2}a_{11}+a_{10}\right)+4b_{20}\left(2s_{1}b_{20}+s_{2}b_{11}+b_{10}\right)\right)\\ & \times \left(4a_{11}\left(s_{1}a_{11}+2s_{2}a_{02}+a_{01}\right)+2b_{11}\left(s_{1}b_{11}+2s_{2}b_{02}+b_{01}\right)\right)+\left(2{\left(s_{1}a_{11}+2s_{2}a_{02}+a_{01}\right)}^{2}+{\left(s_{1}b_{11}+2s_{2}b_{02}+b_{01}\right)}^{2}\right)\\ & \times {\left(4a_{11}\left(2s_{1}a_{20}+s_{2}a_{11}+a_{10}\right)+2b_{11}\left(2s_{1}b_{20}+s_{2}b_{11}+b_{10}\right)\right)}^{2}-8\left(2a_{11}^{2}+b_{11}^{2}\right)\\ & \times \left(2{\left(s_{1}a_{11}+2s_{2}a_{02}+a_{01}\right)}^{2}+{\left(s_{1}b_{11}+2s_{2}b_{02}+b_{01}\right)}^{2}\right)\left(2{\left(2s_{1}a_{20}+s_{2}a_{11}+a_{10}\right)}^{2}+{\left(2s_{1}b_{20}+s_{2}b_{11}+b_{10}\right)}^{2}\right)\\ & +\left(8a_{02}\left(s_{1}a_{11}+2s_{2}a_{02}+a_{01}\right)+4b_{02}\left(s_{1}b_{11}+2s_{2}b_{02}+b_{01}\right)\right)\left(4a_{11}\left(2s_{1}a_{20}+s_{2}a_{11}+a_{10}\right)+2b_{11}\left(2s_{1}b_{20}+s_{2}b_{11}+b_{10}\right)\right)\\ & \times \left(2{\left(2s_{1}a_{20}+s_{2}a_{11}+a_{10}\right)}^{2}+{\left(2s_{1}b_{20}+s_{2}b_{11}+b_{10}\right)}^{2}\right)){\left(-2\right)}^{-1}]F{\left(s_{1},s_{2}\right)}^{-2}, \end{array}$$

in which

(90) $$\begin{array}{cc} F(s_{1},s_{2}) & =H_{1}\left(s_{1},s_{2}\right)H_{2}\left(s_{1},s_{2}\right)\\ & =\left[\left(2{\left(s_{1}a_{11}+2s_{2}a_{02}+a_{01}\right)}^{2}+{\left(s_{1}b_{11}+2s_{2}b_{02}+b_{01}\right)}^{2}\right)\right]\\ & \times \left[\left(2{\left(2s_{1}a_{20}+s_{2}a_{11}+a_{10}\right)}^{2}+{\left(2s_{1}b_{20}+s_{2}b_{11}+b_{10}\right)}^{2}\right)\right]. \end{array}$$

Indeed, it is evident from the computation of R that Equation (89) is a real multivariable scalar function. From the scalar curvature associated with the quadratic system, we now devote ourselves to the geometrical interpretation of the sign and magnitude of R. The positiveness of Equation (89) depends upon the following lemma:

**Lemma** **1.** *The sum of the indices at each of the equilibrium points $p_{1},\ldots ,p_{k}$ of the dynamical system under study, Equation (87), is positive, and so, we have R>0 if $a_{10}b_{01}>b_{10}a_{01}$.*

**Proof.**  According to the positivity condition,

$$R>0\;\mathrm{for}\;\chi \left(M\right)=\sum_{i=1}^{k}I_{i}>0.$$

Therefore, to discuss the sign of Equation (89), one must demonstrate that χ(M)>0 for Equation (87).

Following the program outlined in the preceding sections, we may observe from Equation (87) that $P_{2}\left(s_{1},s_{2}\right)$ and $Q_{2}\left(s_{1},s_{2}\right)$ are real homogeneous polynomials of degree 2 with no common linear factors. It should be remarked that the polynomials $P_{n}\left(s_{1},s_{2}\right)$ and $Q_{n}\left(s_{1},s_{2}\right)$ are said to be homogeneous if all their nonzero terms have the same total degree [104,105]. Furthermore, homogeneous polynomials with no common linear factors are said to be those for which $s_{1}Q_{1}-s_{2}P_{1}\neq 0$.

Owing to the fact that the nonzero terms of the polynomials involved in Equation (89) have the same total degree and $s_{1}Q_{1}-s_{2}P_{1}\neq 0$, we then are led to conclude that Equation (87) has a unique equilibrium point at the origin [27,105]. Having determined the critical point of the dynamical system under study, we can immediately evaluate the Jacobian matrix of Equation (87) in $p_{1}=\left(0,0\right)$, thus obtaining

(91) $$J\left(p_{1}\right)=\left[\begin{array}{cc} a_{10} & a_{01}\\ b_{10} & b_{01} \end{array}\right].$$

From this, it naturally follows that

(92) $$\begin{array}{c} \det\;J=a_{10}b_{01}-b_{10}a_{01},\;\mathrm{Tr}\;J=a_{10}+b_{01},\\ \;{\left(\mathrm{Tr}\;J\right)}^{2}-4\left(\det\;J\right)={\left(a_{10}-b_{01}\right)}^{2}+4a_{01}b_{10}. \end{array}$$

Bearing this in mind, for the Euler characteristic of M, or equivalently the index $I_{1}\left(p_{1}\right)$, to be positive, we must now demonstrate that the equilibrium point of Equation (87) cannot be a saddle type because the index of a saddle is negative. Since the determinant of the Jacobian matrix has the value of $\det\;J=a_{10}b_{01}-b_{10}a_{01}$, a necessary condition for the positiveness of χ(M) is that detJ>0. That is, $a_{10}b_{01}>b_{10}a_{01}$.

According to this necessary condition upon the real linear factors, we then immediately identify the index $I_{1}\left(p_{1}\right)$ or the Euler characteristic of M to be

(93) $$\chi \left(M\right)=I_{1}\left(p_{1}\right)=1.$$

From the hypothesis that the index $I_{1}$ of the equilibrium point $p_{1}=\left(0,0\right)$ is positive, it follows that χ(M)=1 for the necessary condition that $a_{10}b_{01}>b_{10}a_{01}$. Hence, Equations (28) and (93) immediately imply that R>0. This completes the proof of the lemma. □

On account of the positivity of Equation (93) under the necessary condition that $a_{10}b_{01}>b_{10}a_{01}$, we then are led to conclude that the dissipative second-degree polynomial system Equation (87) has isolated periodic orbits in the phase portrait, because of the geometrical interpretation of the sign of the curvature R and the covariant definition of limit cycles. Nevertheless, that raises the mathematical question of the two-dimensional version of the sixteenth problem of Hilbert: What is the maximum number and positions for the limit cycles (isolated periodic trajectories) of Equation (87)?

In the framework of GBT, we define a limit cycle as the periodic state in which the scalar curvature R is positive and whose magnitude, |R|, diverges to infinity at non-symmetrical singularities. With this definition in mind, one way to answer the question raised above is by imposing that Equation (90) should vanish at the critical points of the magnitude of scalar curvature because |R| is continuous except at the critical points $p_{i}^{C}$, in which $F(s_{1},s_{2})$ is zero. Consequently, in order to determine the critical points $p_{i}^{C}$ such that the magnitude of R diverges to infinity, one must seek the exact solutions of the homogeneous polynomial equation

(94) $$F\left(s_{1},s_{2}\right)=H_{1}\left(s_{1},s_{2}\right)H_{2}\left(s_{1},s_{2}\right)=0,$$

which naturally poses a challenge since the exact solutions of algebraic equations, such as Equation (94), or roots of polynomial expressions, Equation (90), are difficult to obtain.

Nonetheless, this apparent difficulty may be resolved by a deeper level of analysis and understanding of the curvature R presented above. On recalling the definition of algebraic curves (see Appendix A), we may recognize that Equation (94) becomes, in fact, an algebraic plane curve as a natural consequence of the restriction that imposes $F(s_{1},s_{2})=0$ at the singularities of the magnitude of scalar curvature.

With the aid of the theorem of Bézout, which is a generalization of the fundamental theorem of algebra, and the theory of resultants of polynomial equations, the number of common points of Equation (94) and its correspondent asymptotes is equal to the number of real solutions of Equation (94). This comprehension is more general than the original description of the problem and certainly enlarges our perspective about the curvature R since we then are led to conclude that the problem of finding the singularities of the magnitude of scalar curvature is, therefore, equivalent to determining the intersection points of Equation (94) with its inclined asymptotes [106,107,108,109,110,111,112,113,114].

Although the understanding outlined above seems to clarify the problem of finding the singularities of |R|, proper mathematical development is required to allow a more accurate conclusion. Thus, we now seek a method that enables us to retain the evident relation between singularities of the magnitude of R and asymptotes of algebraic curves.

The desired approach is to be found by the method of geometric characteristics, which provides a general method for constructing the zeroes or roots of Equation (90) subject to Equation (94) stated above [84,85]. The Appendix A of this paper is devoted to introducing the method and presenting its general procedure for determining the singularities of |R|.

In light of the foregoing, we now dedicate ourselves to the singularities of the magnitude of Equation (89), for which the method outlined above is capable of giving an exact solution. Closely following the program of the Appendix A, we then find exactly four singular points for the magnitude of scalar curvature, namely,

(95) $$p_{1}^{C}=p_{2}^{C}=\left(\frac{a_{01}b_{02}-a_{02}b_{01}}{a_{02}b_{11}-a_{11}b_{02}},\frac{a_{01}b_{11}-a_{11}b_{01}}{2\left(a_{11}b_{02}-a_{02}b_{11}\right)}\right),$$

and

(96) $$p_{3}^{C}=p_{4}^{C}=\left(-\frac{a_{10}b_{11}-a_{11}b_{10}}{2\left(a_{20}b_{11}-a_{11}b_{20}\right)},\frac{a_{10}b_{20}-a_{20}b_{10}}{a_{20}b_{11}-a_{11}b_{20}}\right),$$

from which we conclude that the solutions of Equation (94), or the zeroes of Equation (90), are of multiplicity 2. A rederivation of the critical points obtained above by employing the method of geometric characteristics is given in the Appendix A.

As a consistency check, we now verify explicitly that Equations (95) and (96) satisfy Equation (94), which implies that $|R|\to \infty \;\mathrm{when}\;F(s_{1},s_{2})\to 0$. To this end, we substitute each of the critical points above into Equation (90).

In this way, we then verify that

(97) $$\begin{array}{cc} F(p_{1,2}^{C}) & =[2{\left(a_{01}+\frac{a_{11}\left(a_{01}b_{02}-a_{02}b_{01}\right)}{a_{02}b_{11}-a_{11}b_{02}}+\frac{a_{02}\left(a_{01}b_{11}-a_{11}b_{01}\right)}{a_{11}b_{02}-a_{02}b_{11}}\right)}^{2}\\ & +{\left(b_{01}+\frac{b_{11}\left(a_{01}b_{02}-a_{02}b_{01}\right)}{a_{02}b_{11}-a_{11}b_{02}}+\frac{b_{02}\left(a_{01}b_{11}-a_{11}b_{01}\right)}{a_{11}b_{02}-a_{02}b_{11}}\right)}^{2}\;]\\ & \times [2{\left(a_{10}+\frac{2a_{20}\left(a_{01}b_{02}-a_{02}b_{01}\right)}{a_{02}b_{11}-a_{11}b_{02}}+\frac{a_{11}\left(a_{01}b_{11}-a_{11}b_{01}\right)}{2(a_{11}b_{02}-a_{02}b_{11})}\right)}^{2}\\ & +{\left(b_{10}+\frac{2b_{20}\left(a_{01}b_{02}-a_{02}b_{01}\right)}{a_{02}b_{11}-a_{11}b_{02}}+\frac{b_{11}\left(a_{01}b_{11}-a_{11}b_{01}\right)}{2(a_{11}b_{02}-a_{02}b_{11})}\right)}^{2}\;]=0, \end{array}$$

and

(98) $$\begin{array}{cc} F(p_{3,4}^{C}) & =[2{\left(\frac{2a_{02}\left(a_{10}b_{20}-a_{20}b_{10}\right)}{a_{20}b_{11}-a_{11}b_{20}}-\frac{a_{11}\left(a_{10}b_{11}-a_{11}b_{10}\right)}{2(a_{20}b_{11}-a_{11}b_{20})}+a_{01}\right)}^{2}\\ & +{\left(\frac{2b_{02}\left(a_{10}b_{20}-a_{20}b_{10}\right)}{a_{20}b_{11}-a_{11}b_{20}}-\frac{b_{11}\left(a_{10}b_{11}-a_{11}b_{10}\right)}{2(a_{20}b_{11}-a_{11}b_{20})}+b_{01}\right)}^{2}\;]\\ & \times [2{\left(-\frac{a_{20}\left(a_{10}b_{11}-a_{11}b_{10}\right)}{a_{20}b_{11}-a_{11}b_{20}}+\frac{a_{11}\left(a_{10}b_{20}-a_{20}b_{10}\right)}{a_{20}b_{11}-a_{11}b_{20}}+a_{10}\right)}^{2}\\ & +{\left(-\frac{b_{20}\left(a_{10}b_{11}-a_{11}b_{10}\right)}{a_{20}b_{11}-a_{11}b_{20}}+\frac{b_{11}\left(a_{10}b_{20}-a_{20}b_{10}\right)}{a_{20}b_{11}-a_{11}b_{20}}+b_{10}\right)}^{2}\;]=0. \end{array}$$

Therefore, from the hypothesis that the dynamical system under consideration is a second-degree polynomial system with no common linear factors and the necessary condition that $a_{10}b_{01}>b_{10}a_{01}$, it has been demonstrated that the scalar curvature of Equation (87) is a positive multivariable function and the magnitude of R diverges to infinity at four real singular points. From the definition of limit cycles in the framework of GBT, the total number of divergences of the magnitude of R to infinity gives the maximum number of limit cycles whose relative positions are determined by the singular points of |R|.

On account of the fact that the scalar curvature of the dissipative polynomial system of degree two, Equation (87), is positive and the magnitude of R diverges to infinity at four real singular points, it follows, together with the definition of limit cycles, that the dynamical system under consideration, Equation (87), has the maximum number of four limit cycles, with their relative positions given by the critical points, Equations (95) and (96), where the magnitude of the scalar curvature becomes singular. This is the result that we desired to demonstrate, thus completing the proof. □

With these lessons before us, we then conclude that polynomial systems of degree two, Equation (87), under the appropriate and necessary consideration that $a_{10}b_{01}>b_{10}a_{01}$, have the maximum number of four isolated periodic trajectories whose relative positions are determined by the critical singular points in which the magnitude of scalar curvature diverges to infinity. This result provides a successful response to the two-dimensional version of the sixteenth problem of Hilbert. In addition, our results again enforce the correctness of GBT and the definition of Section 3, since the results found for the second-degree polynomial system, Equation (87), are in complete agreement with the standpoint of previous works in which several authors have asserted that quadratic polynomial systems have the maximum number of four limit cycles [22,97,101,103].

In the following subsection, we profitably return to the research problem raised above and devote ourselves to the second part of the sixteenth problem of Hilbert.

### 4.2. nth-Degree Polynomial Systems

Armed with the foundations of GBT, we now dedicate ourselves to the problem of the number and relative positions of limit cycles for *n*th-degree polynomial systems. More precisely, we provide the solution to the following problem:

**Problem** **2.** *What is the maximum number and position of Poincaré’s limit cycles (isolated periodic trajectories) for the differential equations*

(99) $$\left\{\begin{array}{c} \frac{ds_{1}}{d\tau }=P_{n}\left(s_{1},s_{2}\right)=\sum_{j=0}^{n}\sum_{i=0}^{j}a_{i\;j-i}{\left(s_{1}\right)}^{i}{\left(s_{2}\right)}^{j-i},\\ \frac{ds_{2}}{d\tau }=Q_{n}\left(s_{1},s_{2}\right)=\sum_{j=0}^{n}\sum_{i=0}^{j}b_{i\;j-i}{\left(s_{1}\right)}^{i}{\left(s_{2}\right)}^{j-i}. \end{array}\right.$$

As one may observe, this is the proper second part of Hilbert’s sixteenth problem, whose corresponding solution is provided by demonstrating the following theorem:

**Theorem** **4.** *In the framework of GBT, if the dynamical system under consideration is described by a real polynomial system of degree n≥2 with no common linear factors, Equation (99), then it has the maximum number of 2(n−1)(4(n−1)−2) limit cycles and the relative positions of these isolated periodic trajectories are to be determined by the singularities of the magnitude of the scalar curvature of the nth-degree polynomial system.*

**Proof.**  To verify the statements of that theorem and demonstrate that polynomial systems of degree n≥2 have the maximum number of 2(n−1)(4(n−1)−2) isolated periodic trajectories with their relative positions given by the singularities of the magnitude of the scalar curvature of the system under study, one must show that this result is consistent with the definition of limit cycles from the viewpoint of GBT.

Consequently, we must prove that the corresponding scalar curvature of Equation (99) is positive and the magnitude of R becomes singular at 2(n−1)(4(n−1)−2) critical points. With this purpose in mind, we must treat the cases n=2 and n>2 separately.

From Theorem 3, however, we have already demonstrated that the scalar curvature of the dissipative polynomial system of degree two, Equation (87), is a positive multivariate function, under the necessary condition that $a_{10}b_{01}>b_{10}a_{01}$. Further, we have proved that the magnitude of R diverges to infinity at four singular points. On account of the fact that the scalar curvature of the dissipative polynomial system of degree two, Equation (87), is positive and the magnitude of R has four real singularities, it follows, together with the covariant definition of limit cycles, that quadratic polynomial systems with degree n=2 have the maximum number of four limit cycles, with their relative positions being determined by the critical points, Equations (95) and (96), where the magnitude of the scalar curvature becomes singular. Since 4=2(2−1)(4(2−1)−2) for n=2, the two sides agree, and this completes the proof of the first case by virtue of Theorem 3.

Hence, it is now our task to demonstrate the statements of Theorem 4 for n>2. Based on the above, this proof is organized as follows. First, we derive the corresponding Riemannian metric of Equation (99) from the geometrical methods of GBT and Equations (5)–(8). Second, we dedicate ourselves to the computation of the scalar curvature of the state space of Equation (99). Third, we devote ourselves to the sign interpretation of R to determine the character of the trajectories of the state space. Finally, we devote ourselves to the singularities of the magnitude of the scalar curvature by employing the methods developed in the preceding pages. Again, following the program of Section 2 and Section 3, the corresponding metric of Equation (99) is then written as follows:

(100) $$\begin{array}{c} d\ell^{2}=2[(\;\sum_{j=0}^{n}\sum_{i=0}^{j}is_{1}^{i-1}s_{2}^{j-i}a_{i,j-i}{)}^{2}+(\;\sum_{j=0}^{n}\sum_{i=0}^{j}is_{1}^{i-1}s_{2}^{j-i}b_{i,j-i}{)}^{2}]{\left(ds_{1}\right)}^{2}\\ +2[(\;\sum_{j=0}^{n}\sum_{i=0}^{j}s_{1}^{i}\left(js_{2}^{-i+j-1}-is_{2}^{-i+j-1}\right)a_{i,j-i}{)}^{2}+(\;\sum_{j=0}^{n}\sum_{i=0}^{j}s_{1}^{i}\left(js_{2}^{-i+j-1}-is_{2}^{-i+j-1}\right)b_{i,j-i}{)}^{2}]{\left(ds_{2}\right)}^{2}, \end{array}$$

which is a positive definite and geometrically invariant Riemannian metric.

The next step relies on the computation of R. The Riemannian metric, Equation (100), imposes a curvature R on the two-dimensional manifold of the parameter space of the dynamical system under consideration. After going through some straightforward algebraic manipulations, the scalar curvature R related to the *n*th-degree polynomial system Equation (99) is given by

(101) $$\begin{array}{cc} R & =-\Upsilon_{n}{\left(s_{1},s_{2}\right)}^{-2}\\ \; & \times [\{\;(\;\sum_{j=0}^{n}\sum_{i=0}^{j}s_{1}^{i}\left(js_{2}^{-i+j-1}-is_{2}^{-i+j-1}\right)a_{ij-i}{)}^{2}+(\;\sum_{j=0}^{n}\sum_{i=0}^{j}s_{1}^{i}\left(js_{2}^{-i+j-1}-is_{2}^{-i+j-1}\right)b_{ij-i}{)}^{2}\;\}\\ & \times \{(\;\sum_{j=0}^{n}\sum_{i=0}^{j}is_{1}^{i-1}s_{2}^{j-i}a_{ij-i})\sum_{j=0}^{n}\sum_{i=0}^{j}is_{1}^{i-1}\left(js_{2}^{-i+j-1}-is_{2}^{-i+j-1}\right)a_{ij-i}\\ & +(\;\sum_{j=0}^{n}\sum_{i=0}^{j}is_{1}^{i-1}s_{2}^{j-i}b_{ij-i})\sum_{j=0}^{n}\sum_{i=0}^{j}is_{1}^{i-1}\left(js_{2}^{-i+j-1}-is_{2}^{-i+j-1}\right)b_{ij-i}{\}}^{2}\\ & +\{(\;\sum_{j=0}^{n}\sum_{i=0}^{j}is_{1}^{i-1}s_{2}^{j-i}a_{ij-i}{)}^{2}+(\;\sum_{j=0}^{n}\sum_{i=0}^{j}is_{1}^{i-1}s_{2}^{j-i}b_{ij-i}{)}^{2}\}\\ & \times \{(\;\sum_{j=0}^{n}\sum_{i=0}^{j}s_{1}^{i}\left(js_{2}^{-i+j-1}-is_{2}^{-i+j-1}\right)a_{ij-i})\sum_{j=0}^{n}\sum_{i=0}^{j}s_{1}^{i}(j\left(-is_{2}^{-i+j-2}+js_{2}^{-i+j-2}-s_{2}^{-i+j-2}\right)\\ & -i\left(-is_{2}^{-i+j-2}+js_{2}^{-i+j-2}-s_{2}^{-i+j-2}\right))a_{ij-i}+(\;\sum_{j=0}^{n}\sum_{i=0}^{j}s_{1}^{i}\left(js_{2}^{-i+j-1}-is_{2}^{-i+j-1}\right)b_{ij-i}\;)\\ & \times \sum_{j=0}^{n}\sum_{i=0}^{j}s_{1}^{i}\left(j\left(-is_{2}^{-i+j-2}+js_{2}^{-i+j-2}-s_{2}^{-i+j-2}\right)-i\left(-is_{2}^{-i+j-2}+js_{2}^{-i+j-2}-s_{2}^{-i+j-2}\right)\right)b_{ij-i}\}\\ & \times \{((\;\sum_{j=0}^{n}\sum_{i=0}^{j}is_{1}^{i-1}s_{2}^{j-i}a_{ij-i})\sum_{j=0}^{n}\sum_{i=0}^{j}is_{1}^{i-1}\left(js_{2}^{-i+j-1}-is_{2}^{-i+j-1}\right)a_{ij-i}\\ & +(\;\sum_{j=0}^{n}\sum_{i=0}^{j}is_{1}^{i-1}s_{2}^{j-i}b_{ij-i})\sum_{j=0}^{n}\sum_{i=0}^{j}is_{1}^{i-1}\left(js_{2}^{-i+j-1}-is_{2}^{-i+j-1}\right)b_{ij-i})\\ \; & +((\;\sum_{j=0}^{n}\sum_{i=0}^{j}is_{1}^{i-1}s_{2}^{j-i}a_{ij-i}{)}^{2}+(\;\sum_{j=0}^{n}\sum_{i=0}^{j}is_{1}^{i-1}s_{2}^{j-i}b_{ij-i}{)}^{2})\}\\ \; & \times \{(\;\sum_{j=0}^{n}\sum_{i=0}^{j}is_{1}^{i-1}\left(js_{2}^{-i+j-1}-is_{2}^{-i+j-1}\right)a_{ij-i})\sum_{j=0}^{n}\sum_{i=0}^{j}s_{1}^{i}\left(js_{2}^{-i+j-1}-is_{2}^{-i+j-1}\right)a_{ij-i}\\ & +(\;\sum_{j=0}^{n}\sum_{i=0}^{j}is_{1}^{i-1}\left(js_{2}^{-i+j-1}-is_{2}^{-i+j-1}\right)b_{ij-i})\sum_{j=0}^{n}\sum_{i=0}^{j}s_{1}^{i}\left(js_{2}^{-i+j-1}-is_{2}^{-i+j-1}\right)b_{ij-i}{\}}^{2}\\ & +\{(\;\sum_{j=0}^{n}\sum_{i=0}^{j}is_{1}^{i-1}s_{2}^{j-i}a_{ij-i})\sum_{j=0}^{n}\sum_{i=0}^{j}i\left(is_{1}^{i-2}-s_{1}^{i-2}\right)s_{2}^{j-i}a_{ij-i}\\ & +(\;\sum_{j=0}^{n}\sum_{i=0}^{j}is_{1}^{i-1}s_{2}^{j-i}b_{ij-i})\sum_{j=0}^{n}\sum_{i=0}^{j}i\left(is_{1}^{i-2}-s_{1}^{i-2}\right)s_{2}^{j-i}b_{ij-i}\}\\ \; & \times \{(\;\sum_{j=0}^{n}\sum_{i=0}^{j}is_{1}^{i-1}\left(js_{2}^{-i+j-1}-is_{2}^{-i+j-1}\right)a_{ij-i})\sum_{j=0}^{n}\sum_{i=0}^{j}s_{1}^{i}\left(js_{2}^{-i+j-1}-is_{2}^{-i+j-1}\right)a_{ij-i}\\ \; & +(\;\sum_{j=0}^{n}\sum_{i=0}^{j}is_{1}^{i-1}\left(js_{2}^{-i+j-1}-is_{2}^{-i+j-1}\right)b_{ij-i})\sum_{j=0}^{n}\sum_{i=0}^{j}s_{1}^{i}\left(js_{2}^{-i+j-1}-is_{2}^{-i+j-1}\right)b_{ij-i}\}\\ \; & \times \{(\;\sum_{j=0}^{n}\sum_{i=0}^{j}s_{1}^{i}\left(js_{2}^{-i+j-1}-is_{2}^{-i+j-1}\right)a_{ij-i}{)}^{2}+(\;\sum_{j=0}^{n}\sum_{i=0}^{j}s_{1}^{i}\left(js_{2}^{-i+j-1}-is_{2}^{-i+j-1}\right)b_{ij-i}{)}^{2}\}\\ & -\{(\;\sum_{j=0}^{n}\sum_{i=0}^{j}is_{1}^{i-1}s_{2}^{j-i}a_{ij-i}{)}^{2}+(\;\sum_{j=0}^{n}\sum_{i=0}^{j}is_{1}^{i-1}s_{2}^{j-i}b_{ij-i}{)}^{2}\}\\ \; & \times \{(\;\sum_{j=0}^{n}\sum_{i=0}^{j}s_{1}^{i}\left(js_{2}^{-i+j-1}-is_{2}^{-i+j-1}\right)a_{ij-i}{)}^{2}+(\;\sum_{j=0}^{n}\sum_{i=0}^{j}s_{1}^{i}\left(js_{2}^{-i+j-1}-is_{2}^{-i+j-1}\right)b_{ij-i}{)}^{2}\}\\ & \times \{(\;\sum_{j=0}^{n}\sum_{i=0}^{j}is_{1}^{i-1}\left(js_{2}^{-i+j-1}-is_{2}^{-i+j-1}\right)a_{ij-i}{)}^{2}+(\;\sum_{j=0}^{n}\sum_{i=0}^{j}is_{1}^{i-1}\left(js_{2}^{-i+j-1}-is_{2}^{-i+j-1}\right)b_{ij-i}{)}^{2}\\ & +(\;\sum_{j=0}^{n}\sum_{i=0}^{j}s_{1}^{i}\left(js_{2}^{-i+j-1}-is_{2}^{-i+j-1}\right)a_{ij-i})\sum_{j=0}^{n}\sum_{i=0}^{j}i\left(is_{1}^{i-2}-s_{1}^{i-2}\right)\left(js_{2}^{-i+j-1}-is_{2}^{-i+j-1}\right)a_{ij-i}\\ & +(\;\sum_{j=0}^{n}\sum_{i=0}^{j}s_{1}^{i}\left(js_{2}^{-i+j-1}-is_{2}^{-i+j-1}\right)b_{ij-i})\sum_{j=0}^{n}\sum_{i=0}^{j}i\left(is_{1}^{i-2}-s_{1}^{i-2}\right)\left(js_{2}^{-i+j-1}-is_{2}^{-i+j-1}\right)b_{ij-i}\}\\ & -\{(\;\sum_{j=0}^{n}\sum_{i=0}^{j}is_{1}^{i-1}s_{2}^{j-i}a_{ij-i}{)}^{2}+(\;\sum_{j=0}^{n}\sum_{i=0}^{j}is_{1}^{i-1}s_{2}^{j-i}b_{ij-i}{)}^{2}\}\\ & \times \{(\;\sum_{j=0}^{n}\sum_{i=0}^{j}s_{1}^{i}\left(js_{2}^{-i+j-1}-is_{2}^{-i+j-1}\right)a_{ij-i}{)}^{2}+(\;\sum_{j=0}^{n}\sum_{i=0}^{j}s_{1}^{i}\left(js_{2}^{-i+j-1}-is_{2}^{-i+j-1}\right)b_{ij-i}{)}^{2}\}], \end{array}$$

in which

(102) $$\begin{array}{c} \Upsilon_{n}\left(s_{1},s_{2}\right)=[(\;\sum_{j=0}^{n}\sum_{i=0}^{j}s_{2}^{j-i}\left(\frac{\partial }{\partial s_{1}}s_{1}^{i}\right)a_{i\;j-i}{)}^{2}+(\sum_{j=0}^{n}\sum_{i=0}^{j}s_{2}^{j-i}\left(\frac{\partial }{\partial s_{1}}s_{1}^{i}\right)b_{i\;j-i}{)}^{2}]\\ \times [(\;\sum_{j=0}^{n}\sum_{i=0}^{j}s_{1}^{i}\left(\frac{\partial }{\partial s_{2}}s_{2}^{j-i}\right)a_{i\;j-i}{)}^{2}+\left(\;\sum_{j=0}^{n}\sum_{i=0}^{j}s_{1}^{i}{\left(\frac{\partial }{\partial s_{2}}s_{2}^{j-i})b_{i\;j-i}\right)}^{2}\;\right]. \end{array}$$

It is evident from the computation of R that Equation (101) is a real multivariable scalar function. With the knowledge of R, we may now devote ourselves to the geometrical interpretation of its sign and magnitude.

The positiveness of Equation (101) depends upon the following lemma:

**Lemma** **2.** *The sum of the indices at each of the equilibrium points $p_{1},\ldots ,p_{k}$ of the dynamical system under study, Equation (99), is positive, and so, we have R>0 if $a_{10}b_{01}>b_{10}a_{01}$.*

**Proof.**  According to the positivity condition,

(103) $$R>0\;\mathrm{for}\;\chi \left(M\right)=\sum_{i=1}^{k}I_{i}>0.$$

Therefore, to determine the sign of Equation (101) we must demonstrate that χ(M)>0 for Equation (99).

Following closely the program outlined in the preceding sections, it is not difficult to recognize from Equation (99) that $P_{n}\left(s_{1},s_{2}\right)$ and $Q_{n}\left(s_{1},s_{2}\right)$ are real homogeneous polynomials of degree *n* with no common real linear factors. Since the nonzero terms of the polynomials involved in Equation (101) have the same total degree and $s_{1}Q_{n}-s_{2}P_{n}\neq 0$, we thus are led to conclude that the systems $ds_{1}/d\tau =P_{n}\left(s_{1},s_{2}\right)$ and $ds_{2}/d\tau =Q_{n}\left(s_{1},s_{2}\right)$ have a unique critical point at p=(0,0). Under the linearization of the *n*th-degree polynomial system about the origin, which permits the neglect of all but linear terms of Equation (99), the Jacobian matrix of Equation (99) evaluated in p=(0,0) becomes

(104) $$J\left(p_{1}\right)=\left[\begin{array}{cc} a_{10} & a_{01}\\ b_{10} & b_{01} \end{array}\right].$$

From this, it follows that

(105) $$\begin{array}{c} \det\;J=a_{10}b_{01}-b_{10}a_{01},\;\mathrm{Tr}J=a_{10}+b_{01}, \end{array}$$

(106) $$\begin{array}{c} {\left(\mathrm{Tr}\;J\right)}^{2}-4\left(\det\;J\right)={\left(a_{10}-b_{01}\right)}^{2}+4a_{01}b_{10}. \end{array}$$

In as much as the determinant of the Jacobian matrix has the value of $\det\;J=a_{10}b_{01}-b_{10}a_{01}$, it is evident, once again, that a necessary condition for the positiveness of χ(M) is that detJ>0, or equivalently, that $a_{10}b_{01}>b_{10}a_{01}$. Bearing this in mind, one may immediately identify the index I(p)=I((0,0)) or the Euler characteristic of M to be

(107) χ(M)=1.

From the hypothesis that the index at the equilibrium point p=(0,0) of Equation (99) is positive, it then follows that χ(M)=1, by virtue of the necessary condition that $a_{10}b_{01}>b_{10}a_{01}$. Hence, Equations (103) and (107) immediately imply that R>0, and this completes the proof of the lemma. □

On account of the positivity of Equation (101) under the necessary condition that $a_{10}b_{01}>b_{10}a_{01}$, we then are led to conclude that the *n*th-degree polynomial system Equation (99) has isolated periodic orbits in the phase portrait, because of the geometrical interpretation of the sign of curvature R and the definition of limit cycles constructed in Section 3. That brings us to the mathematical question of the sixteenth problem of Hilbert: What should be the maximum number and positions for the limit cycles of Equation (99)?

From the definition of limit cycles in the framework of GBT, the total number of divergences of the magnitude of R to infinity gives the maximum number of limit cycles of Equation (99), with their relative positions being determined by the singularities of |R|. With this definition before us, one way to answer the question raised above is by determining the maximum number of singularities of the magnitude of the scalar curvature, which thus requires us to impose that Equation (102) must be zero.

More specifically, to determine the singular points $p_{i}^{C}$ such that the magnitude of the scalar curvature diverges to infinity, we must then direct our attention to the challenge of finding the total number of solutions of

(108) $$\begin{array}{c} \Upsilon_{n}\left(s_{1},s_{2}\right)=[(\;\sum_{j=0}^{n}\sum_{i=0}^{j}s_{2}^{j-i}\left(\frac{\partial }{\partial s_{1}}s_{1}^{i}\right)a_{i\;j-i}{)}^{2}+(\sum_{j=0}^{n}\sum_{i=0}^{j}s_{2}^{j-i}\left(\frac{\partial }{\partial s_{1}}s_{1}^{i}\right)b_{i\;j-i}{)}^{2}]\\ \times [(\;\sum_{j=0}^{n}\sum_{i=0}^{j}s_{1}^{i}\left(\frac{\partial }{\partial s_{2}}s_{2}^{j-i}\right)a_{i\;j-i}{)}^{2}+\left(\;\sum_{j=0}^{n}\sum_{i=0}^{j}s_{1}^{i}{\left(\frac{\partial }{\partial s_{2}}s_{2}^{j-i})b_{i\;j-i}\right)}^{2}\;\right]=0, \end{array}$$

which is a real and positive multivariate polynomial equation.

We recall from the method of geometric characteristics that the problem of finding the singular points of the magnitude of a scalar curvature is equivalent to that of finding intersection points of algebraic curves with their asymptotes. As we may recognize, Equation (108) is an algebraic curve of degree 4(n−1), as a consequence of the condition that $\Upsilon_{n}\left(s_{1},s_{2}\right)=0$ at $p_{i}^{C}$. Hence, the number of common points of the denominator of the scalar curvature, Equation (101), and its asymptotes is therefore equal to the number of solutions of Equation (108) by virtue of the well-known Bézout theorem. Hence, in order to determine the total number of solutions of Equation (108), we carry out the program of the method of geometric characteristics mentioned in the Appendix A. For this, however, it proves convenient to rewrite Equation (108) in terms of a more appropriate form. For this purpose, the following lemma is useful.

**Lemma** **3.** *For every natural number n≥2,*

$$\begin{array}{cc} \Upsilon_{n}\left(s_{1},s_{2}\right) & =[(\;\sum_{j=0}^{n}\sum_{i=0}^{j}s_{2}^{j-i}\left(\frac{\partial }{\partial s_{1}}s_{1}^{i}\right)a_{i\;j-i}{)}^{2}+(\sum_{j=0}^{n}\sum_{i=0}^{j}s_{2}^{j-i}\left(\frac{\partial }{\partial s_{1}}s_{1}^{i}\right)b_{i\;j-i}{)}^{2}]\\ & \times [(\;\sum_{j=0}^{n}\sum_{i=0}^{j}s_{1}^{i}\left(\frac{\partial }{\partial s_{2}}s_{2}^{j-i}\right)a_{i\;j-i}{)}^{2}+\left(\;\sum_{j=0}^{n}\sum_{i=0}^{j}s_{1}^{i}{\left(\frac{\partial }{\partial s_{2}}s_{2}^{j-i})b_{i\;j-i}\right)}^{2}\;\right]\\ & =\sum_{j=0}^{4(n-1)}\sum_{i=0}^{4(n-1)-j}{\left(\frac{s_{1}}{s_{2}}\right)}^{-i-j+4(n-1)}s_{2}^{4(n-1)-j}c_{-i-j+4(n-1)\;\;i}\;\;, \end{array}$$

*whence,*

$$c_{-i-j+4(n-1)\;\;i}=\frac{1}{i!\left(-i-j+4(n-1)\right)!}\left(\frac{\partial^{-i-j+4(n-1)}}{\partial s_{1}^{-i-j+4(n-1)}}\frac{\partial^{i}}{\partial s_{2}^{i}}\Upsilon_{n}\left(s_{1},s_{2}\right)\right)\;at\;\left(s_{1},s_{2}\right)=\left(0,0\right),$$

*in which it is understood that $c_{-i-j+4(n-1)\;\;i}$ depends on (a,b).*

**Proof.**  For each n≥2, let S(n) denote the statement

(109) $$\begin{array}{cc} S(n) & :[(\;\sum_{j=0}^{n}\sum_{i=0}^{j}s_{2}^{j-i}\left(\frac{\partial }{\partial s_{1}}s_{1}^{i}\right)a_{i\;j-i}{)}^{2}+(\sum_{j=0}^{n}\sum_{i=0}^{j}s_{2}^{j-i}\left(\frac{\partial }{\partial s_{1}}s_{1}^{i}\right)b_{i\;j-i}{)}^{2}]\\ & \times [(\;\sum_{j=0}^{n}\sum_{i=0}^{j}s_{1}^{i}\left(\frac{\partial }{\partial s_{2}}s_{2}^{j-i}\right)a_{i\;j-i}{)}^{2}+\left(\;\sum_{j=0}^{n}\sum_{i=0}^{j}s_{1}^{i}{\left(\frac{\partial }{\partial s_{2}}s_{2}^{j-i})b_{i\;j-i}\right)}^{2}\;\right] \end{array}$$

(110) $$\begin{array}{cc} & =\sum_{j=0}^{4(n-1)}\sum_{i=0}^{4(n-1)-j}{\left(\frac{s_{2}}{s_{1}}\right)}^{-i-j+4(n-1)}s_{2}^{4(n-1)-j}c_{-i-j+4(n-1)\;\;i}. \end{array}$$

First, let us demonstrate that the statement S(n) holds for n=2, which constitutes the base step of our proof by induction. It is evident for n=2 that the left-hand side of S(2) is

$$\begin{array}{c} \begin{array}{cc} \; & \;\;4\left[\left(a_{11}^{2}+b_{11}^{2}\right)\left(a_{20}^{2}+b_{20}^{2}\right)\right]s_{1}^{4}\\ & +4\left[a_{20}a_{11}^{3}+b_{11}b_{20}a_{11}^{2}+\left(a_{20}b_{11}^{2}+4a_{02}\left(a_{20}^{2}+b_{20}^{2}\right)\right)a_{11}+b_{11}\left(4b_{02}a_{20}^{2}+b_{20}\left(b_{11}^{2}+4b_{02}b_{20}\right)\right)\right]s_{1}^{3}s_{2}\\ & +4\left[b_{10}b_{20}a_{11}^{2}+2a_{01}\left(a_{20}^{2}+b_{20}^{2}\right)a_{11}+2b_{01}b_{11}b_{20}^{2}+2a_{20}^{2}b_{01}b_{11}+a_{10}a_{20}\left(a_{11}^{2}+b_{11}^{2}\right)+b_{10}b_{11}^{2}b_{20}\right]s_{1}^{3}\\ & +\;\;\;[a_{11}^{4}+2\left(b_{11}^{2}+8a_{02}a_{20}\right)a_{11}^{2}+16b_{11}\left(a_{20}b_{02}+a_{02}b_{20}\right)a_{11}+b_{11}^{4}+16a_{20}^{2}b_{02}^{2}+16b_{02}^{2}b_{20}^{2}\\ & +16b_{02}b_{11}^{2}b_{20}+16a_{02}^{2}\left(a_{20}^{2}+b_{20}^{2}\right)]s_{2}^{2}s_{1}^{2}\\ \; & +2[b_{10}b_{11}^{3}+4b_{01}b_{20}b_{11}^{2}+4a_{11}a_{20}b_{01}b_{11}+a_{11}^{2}b_{10}b_{11}+8b_{02}b_{10}b_{20}b_{11}+8b_{01}b_{02}b_{20}^{2}+8a_{20}^{2}b_{01}b_{02}\\ & +a_{10}\left(a_{11}^{3}+\left(b_{11}^{2}+8a_{02}a_{20}\right)a_{11}+8a_{20}b_{02}b_{11}\right)\\ & +8a_{02}a_{11}b_{10}b_{20}+4a_{01}\left(a_{20}a_{11}^{2}+b_{11}b_{20}a_{11}+2a_{02}\left(a_{20}^{2}+b_{20}^{2}\right)\right)]s_{2}s_{1}^{2}\\ \; & +\;\;\;[4\left(a_{20}^{2}+b_{20}^{2}\right)a_{01}^{2}+8a_{11}b_{10}b_{20}a_{01}+4a_{20}^{2}b_{01}^{2}+a_{11}^{2}b_{10}^{2}+b_{10}^{2}b_{11}^{2}+4b_{01}^{2}b_{20}^{2}+8a_{10}a_{20}\left(a_{01}a_{11}+b_{01}b_{11}\right)\\ & +a_{10}^{2}\left(a_{11}^{2}+b_{11}^{2}\right)+8b_{01}b_{10}b_{11}b_{20}]s_{1}^{2}\\ \; & +4\left[4\left(a_{11}a_{20}+b_{11}b_{20}\right)a_{02}^{2}+a_{11}\left(a_{11}^{2}+b_{11}^{2}\right)a_{02}+b_{02}\left(b_{11}^{3}+a_{11}^{2}b_{11}+4b_{02}b_{20}b_{11}+4a_{11}a_{20}b_{02}\right)\right]s_{2}^{3}s_{1}\\ & +2[b_{01}b_{11}^{3}+4b_{02}b_{10}b_{11}^{2}+a_{11}^{2}b_{01}b_{11}+4a_{10}a_{11}b_{02}b_{11}+8b_{01}b_{02}b_{20}b_{11}+8a_{10}a_{20}b_{02}^{2}+8a_{11}a_{20}b_{01}b_{02}\\ & +a_{01}a_{11}\left(a_{11}^{2}+b_{11}^{2}\right)+8b_{02}^{2}b_{10}b_{20}+8a_{02}^{2}\left(a_{10}a_{20}+b_{10}b_{20}\right)+4a_{02}\left(a_{10}a_{11}^{2}+b_{10}b_{11}a_{11}+2a_{01}\left(a_{11}a_{20}+b_{11}b_{20}\right)\right)]s_{1}s_{2}^{2}\\ \; & +4[\left(a_{11}a_{20}+b_{11}b_{20}\right)a_{01}^{2}+a_{11}\left(a_{10}a_{11}+b_{10}b_{11}\right)a_{01}+a_{11}a_{20}b_{01}^{2}+b_{01}b_{10}b_{11}^{2}+4a_{10}a_{20}b_{01}b_{02}+b_{02}b_{10}^{2}b_{11}\\ & +a_{10}a_{11}b_{01}b_{11}+a_{10}^{2}b_{02}b_{11}+4b_{01}b_{02}b_{10}b_{20}+b_{01}^{2}b_{11}b_{20}+a_{02}\left(a_{11}a_{10}^{2}+4a_{01}a_{20}a_{10}+b_{10}\left(a_{11}b_{10}+4a_{01}b_{20}\right)\right)]s_{1}s_{2}\\ \; & +2\left[(2\left(a_{10}a_{20}+b_{10}b_{20}\right)a_{01}^{2}+a_{11}\left(a_{10}^{2}+b_{10}^{2}\right)a_{01}+b_{01}\left(b_{11}a_{10}^{2}+2a_{20}b_{01}a_{10}+b_{10}\left(b_{10}b_{11}+2b_{01}b_{20}\right)\right)\right]s_{1}\\ & +4\left[\left(a_{02}^{2}+b_{02}^{2}\right)\left(a_{11}^{2}+b_{11}^{2}\right)\right]s_{2}^{4}\\ & +4\left[(2\left(a_{10}a_{11}+b_{10}b_{11}\right)a_{02}^{2}+a_{01}\left(a_{11}^{2}+b_{11}^{2}\right)a_{02}+b_{02}\left(b_{01}a_{11}^{2}+2a_{10}b_{02}a_{11}+b_{11}\left(2b_{02}b_{10}+b_{01}b_{11}\right)\right)\right]s_{2}^{3}\\ & +\;\;\;[\left(a_{11}^{2}+b_{11}^{2}\right)a_{01}^{2}+8a_{02}\left(a_{10}a_{11}+b_{10}b_{11}\right)a_{01}+a_{11}^{2}b_{01}^{2}+4a_{10}^{2}b_{02}^{2}+4b_{02}^{2}b_{10}^{2}+b_{01}^{2}b_{11}^{2}+8a_{10}a_{11}b_{01}b_{02}\\ & +4a_{02}^{2}\left(a_{10}^{2}+b_{10}^{2}\right)+8b_{01}b_{02}b_{10}b_{11}]s_{2}^{2}\\ \; & +2\left[\left(a_{10}a_{11}+b_{10}b_{11}\right)a_{01}^{2}+2a_{02}\left(a_{10}^{2}+b_{10}^{2}\right)a_{01}+b_{01}\left(2b_{02}a_{10}^{2}+a_{11}b_{01}a_{10}+b_{10}\left(2b_{02}b_{10}+b_{01}b_{11}\right)\right)\right]s_{2}\\ \; & +\;\;\;\left[\left(a_{01}^{2}+b_{01}^{2}\right)\left(a_{10}^{2}+b_{10}^{2}\right)\right] \end{array} \end{array}$$

and the right-hand side of S(2) is

(111) $$\begin{array}{cc} c_{40}s_{1}^{4}+c_{31}s_{1}^{3}s_{2}+c_{30}s_{1}^{3}+c_{22}s_{1}^{2}s_{2}^{2}+c_{21}s_{1}^{2}s_{2}+c_{20}s_{1}^{2} & +c_{13}s_{1}s_{2}^{3}+c_{12}s_{1}s_{2}^{2}\\ \; & +c_{11}s_{1}s_{2}+c_{10}s_{1}+c_{04}s_{2}^{4}+c_{03}s_{2}^{3}+c_{02}s_{2}^{2}+c_{01}s_{2}+c_{00}, \end{array}$$

where

$$\begin{array}{c} \begin{array}{cc} c_{40} & =\;\;\;\;\;\left[\frac{6}{24}\left(2a_{11}^{2}+2b_{11}^{2}\right)\left(8a_{20}^{2}+8b_{20}^{2}\right)\right],\\ c_{31} & =\frac{1}{6}\left[3\left(2a_{11}^{2}+2b_{11}^{2}\right)\left(4a_{11}a_{20}+4b_{11}b_{20}\right)+3\left(4a_{02}a_{11}+4b_{02}b_{11}\right)\left(8a_{20}^{2}+8b_{20}^{2}\right)\right],\\ c_{30} & =\frac{1}{6}\left[3\left(2a_{11}^{2}+2b_{11}^{2}\right)\left(4a_{10}a_{20}+4b_{10}b_{20}\right)+3\left(2a_{01}a_{11}+2b_{01}b_{11}\right)\left(8a_{20}^{2}+8b_{20}^{2}\right)\right],\\ c_{22} & =\frac{1}{4}\left[{\left(2a_{11}^{2}+2b_{11}^{2}\right)}^{2}+4\left(4a_{02}a_{11}+4b_{02}b_{11}\right)\left(4a_{11}a_{20}+4b_{11}b_{20}\right)+\left(8a_{02}^{2}+8b_{02}^{2}\right)\left(8a_{20}^{2}+8b_{20}^{2}\right)\right], \end{array} \end{array}$$

$$\begin{array}{c} \begin{array}{cc} c_{21} & =\frac{1}{2}[\left(2a_{10}a_{11}+2b_{10}b_{11}\right)\left(2a_{11}^{2}+2b_{11}^{2}\right)+2\left(4a_{02}a_{11}+4b_{02}b_{11}\right)\left(4a_{10}a_{20}+4b_{10}b_{20}\right)\\ & +2\left(2a_{01}a_{11}+2b_{01}b_{11}\right)\left(4a_{11}a_{20}+4b_{11}b_{20}\right)+\left(4a_{01}a_{02}+4b_{01}b_{02}\right)\left(8a_{20}^{2}+8b_{20}^{2}\right)],\\ c_{20} & =\frac{1}{2}\left[\left(a_{10}^{2}+b_{10}^{2}\right)\left(2a_{11}^{2}+2b_{11}^{2}\right)+2\left(2a_{01}a_{11}+2b_{01}b_{11}\right)\left(4a_{10}a_{20}+4b_{10}b_{20}\right)+\left(a_{01}^{2}+b_{01}^{2}\right)\left(8a_{20}^{2}+8b_{20}^{2}\right)\right],\\ c_{13} & =\frac{1}{6}\left[3\left(4a_{02}a_{11}+4b_{02}b_{11}\right)\left(2a_{11}^{2}+2b_{11}^{2}\right)+3\left(8a_{02}^{2}+8b_{02}^{2}\right)\left(4a_{11}a_{20}+4b_{11}b_{20}\right)\right],\\ c_{12} & =\frac{1}{2}[2\left(4a_{02}a_{11}+4b_{02}b_{11}\right)\left(2a_{10}a_{11}+2b_{10}b_{11}\right)+\left(2a_{01}a_{11}+2b_{01}b_{11}\right)\left(2a_{11}^{2}+2b_{11}^{2}\right)\\ & +\left(8a_{02}^{2}+8b_{02}^{2}\right)\left(4a_{10}a_{20}+4b_{10}b_{20}\right)+2\left(4a_{01}a_{02}+4b_{01}b_{02}\right)\left(4a_{11}a_{20}+4b_{11}b_{20}\right)],\\ c_{11} & =[\left(a_{10}^{2}+b_{10}^{2}\right)\left(4a_{02}a_{11}+4b_{02}b_{11}\right)+\left(2a_{01}a_{11}+2b_{01}b_{11}\right)\left(2a_{10}a_{11}+2b_{10}b_{11}\right)\\ & +\left(4a_{01}a_{02}+4b_{01}b_{02}\right)\left(4a_{10}a_{20}+4b_{10}b_{20}\right)+\left(a_{01}^{2}+b_{01}^{2}\right)\left(4a_{11}a_{20}+4b_{11}b_{20}\right)],\\ c_{10} & =\left[\left(a_{10}^{2}+b_{10}^{2}\right)\left(2a_{01}a_{11}+2b_{01}b_{11}\right)+\left(a_{01}^{2}+b_{01}^{2}\right)\left(4a_{10}a_{20}+4b_{10}b_{20}\right)\right],\\ c_{04} & =\frac{6}{24}\left[\left(8a_{02}^{2}+8b_{02}^{2}\right)\left(2a_{11}^{2}+2b_{11}^{2}\right)\right],\\ c_{03} & =\frac{1}{6}\left[3\left(8a_{02}^{2}+8b_{02}^{2}\right)\left(2a_{10}a_{11}+2b_{10}b_{11}\right)+3\left(4a_{01}a_{02}+4b_{01}b_{02}\right)\left(2a_{11}^{2}+2b_{11}^{2}\right)\right],\\ c_{02} & =\frac{1}{2}\left[\left(8a_{02}^{2}+8b_{02}^{2}\right)\left(a_{10}^{2}+b_{10}^{2}\right)+2\left(4a_{01}a_{02}+4b_{01}b_{02}\right)\left(2a_{10}a_{11}+2b_{10}b_{11}\right)+\left(a_{01}^{2}+b_{01}^{2}\right)\left(2a_{11}^{2}+2b_{11}^{2}\right)\right],\\ c_{01} & =\;\;\;\;\left[\left(4a_{01}a_{02}+4b_{01}b_{02}\right)\left(a_{10}^{2}+b_{10}^{2}\right)+\left(a_{01}^{2}+b_{01}^{2}\right)\left(2a_{10}a_{11}+2b_{10}b_{11}\right)\right], \end{array} \end{array}$$

and

$$\begin{array}{cc} c_{00} & =\left[\left(a_{01}^{2}+b_{01}^{2}\right)\left(a_{10}^{2}+b_{10}^{2}\right)\right], \end{array}$$

from which one may conclude that the left- and right-hand sides of S(2) are equal. Consequently, the statement is clearly true for n=2 as well, proving the base case.

The next stage is reached by stating the inductive step. For this purpose, let us fix some k≥2 and assume the inductive hypothesis

(112) $$\begin{array}{cc} S(k) & :\;\;[(\;\sum_{j=0}^{k}\sum_{i=0}^{j}s_{2}^{j-i}\left(\frac{\partial }{\partial s_{1}}s_{1}^{i}\right)a_{i\;j-i}{)}^{2}+(\sum_{j=0}^{k}\sum_{i=0}^{j}s_{2}^{j-i}\left(\frac{\partial }{\partial s_{1}}s_{1}^{i}\right)b_{i\;j-i}{)}^{2}]\\ & \times [(\;\sum_{j=0}^{k}\sum_{i=0}^{j}s_{1}^{i}\left(\frac{\partial }{\partial s_{2}}s_{2}^{j-i}\right)a_{i\;j-i}{)}^{2}+\left(\;\sum_{j=0}^{k}\sum_{i=0}^{j}s_{1}^{i}{\left(\frac{\partial }{\partial s_{2}}s_{2}^{j-i})b_{i\;j-i}\right)}^{2}\;\right]\\ & =\sum_{j=0}^{4(k-1)}\sum_{i=0}^{4(k-1)-j}{\left(\frac{s_{1}}{s_{2}}\right)}^{-i-j+4(k-1)}s_{2}^{4(k-1)-j}c_{-i-j+4(k-1)\;\;i}, \end{array}$$

where

(113) $$c_{-i-j+4(k-1)\;\;i}=\frac{1}{i!\left(-i-j+4(k-1)\right)!}\left(\frac{\partial^{-i-j+4(k-1)}}{\partial s_{1}^{-i-j+4(k-1)}}\frac{\partial^{i}}{\partial s_{2}^{i}}\Upsilon_{k}\left(s_{1},s_{2}\right)\right)\;\mathrm{at}\;\left(s_{1},s_{2}\right)=\left(0,0\right)\;\;\;,$$

to be true.

The decisive step now is to prove that

(114) $$\begin{array}{cc} S(k+1) & :\;\;[(\;\sum_{j=0}^{k+1}\sum_{i=0}^{j}s_{2}^{j-i}\left(\frac{\partial }{\partial s_{1}}s_{1}^{i}\right)a_{i\;j-i}{)}^{2}+(\;\sum_{j=0}^{k+1}\sum_{i=0}^{j}s_{2}^{j-i}\left(\frac{\partial }{\partial s_{1}}s_{1}^{i}\right)b_{i\;j-i}{)}^{2}]\\ & \times [(\;\sum_{j=0}^{k+1}\sum_{i=0}^{j}s_{1}^{i}\left(\frac{\partial }{\partial s_{2}}s_{2}^{j-i}\right)a_{i\;j-i}{)}^{2}+(\;\sum_{j=0}^{k+1}\sum_{i=0}^{j}s_{1}^{i}\left(\frac{\partial }{\partial s_{2}}s_{2}^{j-i}\right)b_{i\;j-i}{)}^{2}]\\ & =\sum_{j=0}^{4k}\sum_{i=0}^{4k-j}{\left(\frac{s_{1}}{s_{2}}\right)}^{-i-j+4k}s_{2}^{4k-j}c_{-i-j+4k\;\;i}\;, \end{array}$$

in which

(115) $$c_{-i-j+4k\;\;i}=\frac{1}{i!\left(-i-j+4k\right)!}\left(\frac{\partial^{-i-j+4k}}{\partial s_{1}^{-i-j+4k}}\frac{\partial^{i}}{\partial s_{2}^{i}}\Upsilon_{k+1}\left(s_{1},s_{2}\right)\right)\;\mathrm{at}\;\left(s_{1},s_{2}\right)=\left(0,0\right).$$

Beginning with the left-hand side of S(k+1), we have

$$\begin{array}{c} \;\;\;\;\;\;[(\;\sum_{j=0}^{k+1}\sum_{i=0}^{j}s_{2}^{j-i}\left(\frac{\partial }{\partial s_{1}}s_{1}^{i}\right)a_{i\;j-i}{)}^{2}+(\;\sum_{j=0}^{k+1}\sum_{i=0}^{j}s_{2}^{j-i}\left(\frac{\partial }{\partial s_{1}}s_{1}^{i}\right)b_{i\;j-i}{)}^{2}]\\ \times [(\;\sum_{j=0}^{k+1}\sum_{i=0}^{j}s_{1}^{i}\left(\frac{\partial }{\partial s_{2}}s_{2}^{j-i}\right)a_{i\;j-i}{)}^{2}+(\;\sum_{j=0}^{k+1}\sum_{i=0}^{j}s_{1}^{i}\left(\frac{\partial }{\partial s_{2}}s_{2}^{j-i}\right)b_{i\;j-i}{)}^{2}]\\ =\;\;\;\;\;\;\left[{\left(\sum_{j=0}^{k}\sum_{i=0}^{j}is_{1}^{i-1}s_{2}^{j-i}a_{i\;j-i}+\sum_{i=0}^{k+1}is_{1}^{i-1}s_{2}^{-i+k+1}a_{i\;-i+k+1}\right)}^{2}\right.\\ \left.\;\;\;\;\;\;\;\;\;\;\;+{\left(\sum_{j=0}^{k}\sum_{i=0}^{j}is_{1}^{i-1}s_{2}^{j-i}b_{i\;j-i}+\sum_{i=0}^{k+1}is_{1}^{i-1}s_{2}^{-i+k+1}b_{i\;-i+k+1}\right)}^{2}\;\right]\\ \times \left[{\left(\sum_{j=0}^{k}\sum_{i=0}^{j}\left(j-i\right)s_{1}^{i}s_{2}^{-i+j-1}a_{i\;j-i}+\sum_{i=0}^{k+1}\left(-i+k+1\right)s_{1}^{i}s_{2}^{k-i}a_{i\;-i+k+1}\right)}^{2}\;\;\;\;\;\;\;\right.\\ \left.\;\;\;\;\;\;\;\;\;+{\left(\sum_{j=0}^{k}\sum_{i=0}^{j}\left(j-i\right)s_{1}^{i}s_{2}^{-i+j-1}b_{i\;j-i}+\sum_{i=0}^{k+1}\left(-i+k+1\right)s_{1}^{i}s_{2}^{k-i}b_{i\;-i+k+1}\right)}^{2}\;\right]\\ =[(\;\sum_{i=0}^{k+1}is_{1}^{i-1}s_{2}^{-i+k+1}a_{i\;-i+k+1}{)}^{2}+(\;\sum_{i=0}^{k+1}is_{1}^{i-1}s_{2}^{-i+k+1}b_{i\;-i+k+1}{)}^{2}\\ \;\;\;\;\;\;+(\;\sum_{j=0}^{k}\sum_{i=0}^{j}is_{1}^{i-1}s_{2}^{j-i}a_{i\;j-i}{)}^{2}+(\;\sum_{j=0}^{k}\sum_{i=0}^{j}is_{1}^{i-1}s_{2}^{j-i}b_{i\;j-i}{)}^{2}\\ +2(\;\sum_{i=0}^{k+1}is_{1}^{i-1}s_{2}^{-i+k+1}b_{i\;-i+k+1})\sum_{j=0}^{k}\sum_{i=0}^{j}is_{1}^{i-1}s_{2}^{j-i}b_{i\;j-i}+2(\;\sum_{i=0}^{k+1}is_{1}^{i-1}s_{2}^{-i+k+1}a_{i\;-i+k+1})\sum_{j=0}^{k}\sum_{i=0}^{j}is_{1}^{i-1}s_{2}^{j-i}a_{i\;j-i}]\\ \times [(\;\sum_{i=0}^{k+1}\left(-i+k+1\right)s_{1}^{i}s_{2}^{k-i}a_{i\;-i+k+1}{)}^{2}+(\;\sum_{i=0}^{k+1}\left(-i+k+1\right)s_{1}^{i}s_{2}^{k-i}b_{i\;-i+k+1}{)}^{2}\;\;\;\;\;\;\;\\ \;\;\;\;\;\;\;\;\;\;\;\;+(\;\sum_{j=0}^{k}\sum_{i=0}^{j}\left(j-i\right)s_{1}^{i}s_{2}^{-i+j-1}b_{i\;j-i}{)}^{2}+(\;\sum_{j=0}^{k}\sum_{i=0}^{j}\left(j-i\right)s_{1}^{i}s_{2}^{-i+j-1}a_{i\;j-i}{)}^{2}\\ +2(\;\sum_{i=0}^{k+1}\left(-i+k+1\right)s_{1}^{i}s_{2}^{k-i}a_{i\;-i+k+1})\sum_{j=0}^{k}\sum_{i=0}^{j}\left(j-i\right)s_{1}^{i}s_{2}^{-i+j-1}a_{i\;j-i}\;\;\;\;\;\;\;\;\;\;\\ +2(\;\sum_{i=0}^{k+1}\left(-i+k+1\right)s_{1}^{i}s_{2}^{k-i}b_{i\;-i+k+1})\sum_{j=0}^{k}\sum_{i=0}^{j}\left(j-i\right)s_{1}^{i}s_{2}^{-i+j-1}b_{i\;j-i}]\;\;\;\;\;\;\\ =[(\;\sum_{j=0}^{k}\sum_{i=0}^{j}\left(j-i\right)s_{1}^{i}s_{2}^{-i+j-1}a_{i\;j-i}{)}^{2}+(\;\sum_{j=0}^{k}\sum_{i=0}^{j}\left(j-i\right)s_{1}^{i}s_{2}^{-i+j-1}b_{i\;j-i}{)}^{2}]\\ \times [(\;\sum_{j=0}^{k}\sum_{i=0}^{j}is_{1}^{i-1}s_{2}^{j-i}a_{i\;j-i}{)}^{2}+(\;\sum_{j=0}^{k}\sum_{i=0}^{j}is_{1}^{i-1}s_{2}^{j-i}b_{i\;j-i}{)}^{2}]\;\;\;\\ +[(\;\sum_{i=0}^{k+1}is_{1}^{i-1}s_{2}^{-i+k+1}a_{i\;-i+k+1}{)}^{2}+(\;\sum_{i=0}^{k+1}is_{1}^{i-1}s_{2}^{-i+k+1}b_{i\;-i+k+1}{)}^{2}]\\ \times [(\;\sum_{j=0}^{k}\sum_{i=0}^{j}\left(j-i\right)s_{1}^{i}s_{2}^{-i+j-1}a_{i\;j-i}{)}^{2}+(\;\sum_{j=0}^{k}\sum_{i=0}^{j}\left(j-i\right)s_{1}^{i}s_{2}^{-i+j-1}b_{i\;j-i}{)}^{2}]\;\;\\ {}[2(\;\sum_{i=0}^{k+1}is_{1}^{i-1}s_{2}^{-i+k+1}a_{i\;-i+k+1})(\;\sum_{j=0}^{k}\sum_{i=0}^{j}is_{1}^{i-1}s_{2}^{j-i}a_{i\;j-i})]\\ \times [(\;\sum_{j=0}^{k}\sum_{i=0}^{j}\left(j-i\right)s_{1}^{i}s_{2}^{-i+j-1}a_{i\;j-i}{)}^{2}+(\;\sum_{j=0}^{k}\sum_{i=0}^{j}\left(j-i\right)s_{1}^{i}s_{2}^{-i+j-1}b_{i\;j-i}{)}^{2}]\\ +2(\;\sum_{i=0}^{k+1}is_{1}^{i-1}s_{2}^{-i+k+1}b_{i\;-i+k+1})\sum_{j=0}^{k}\sum_{i=0}^{j}is_{1}^{i-1}s_{2}^{j-i}b_{i\;j-i}\\ \times [(\;\sum_{j=0}^{k}\sum_{i=0}^{j}\left(j-i\right)s_{1}^{i}s_{2}^{-i+j-1}a_{i\;j-i}{)}^{2}+(\;\sum_{j=0}^{k}\sum_{i=0}^{j}\left(j-i\right)s_{1}^{i}s_{2}^{-i+j-1}b_{i\;j-i}{)}^{2}]\\ +[(\;\sum_{i=0}^{k+1}\left(-i+k+1\right)s_{1}^{i}s_{2}^{k-i}a_{i\;-i+k+1}{)}^{2}+(\;\sum_{i=0}^{k+1}\left(-i+k+1\right)s_{1}^{i}s_{2}^{k-i}b_{i\;-i+k+1}{)}^{2}]\\ \times [(\;\sum_{j=0}^{k}\sum_{i=0}^{j}is_{1}^{i-1}s_{2}^{j-i}a_{i\;j-i}{)}^{2}+(\;\sum_{j=0}^{k}\sum_{i=0}^{j}is_{1}^{i-1}s_{2}^{j-i}b_{i\;j-i}{)}^{2}]\\ +[(\;\sum_{i=0}^{k+1}\left(-i+k+1\right)s_{1}^{i}s_{2}^{k-i}a_{i\;-i+k+1}{)}^{2}+(\;\sum_{i=0}^{k+1}\left(-i+k+1\right)s_{1}^{i}s_{2}^{k-i}b_{i\;-i+k+1}{)}^{2}]\\ \times [(\;\sum_{i=0}^{k+1}is_{1}^{i-1}s_{2}^{-i+k+1}a_{i\;-i+k+1}{)}^{2}+(\;\sum_{i=0}^{k+1}is_{1}^{i-1}s_{2}^{-i+k+1}b_{i\;-i+k+1}{)}^{2}]\\ +2\left(\sum_{i=0}^{k+1}is_{1}^{i-1}s_{2}^{-i+k+1}a_{i\;-i+k+1}\right)\left({\left(\sum_{i=0}^{k+1}\left(-i+k+1\right)s_{1}^{i}s_{2}^{k-i}a_{i\;-i+k+1}\right)}^{2}\right.\\ \left.+{\left(\sum_{i=0}^{k+1}\left(-i+k+1\right)s_{1}^{i}s_{2}^{k-i}b_{i\;-i+k+1}\right)}^{2}\right)\sum_{j=0}^{k}\sum_{i=0}^{j}is_{1}^{i-1}s_{2}^{j-i}a_{i\;j-i}\\ +2\left(\sum_{i=0}^{k+1}is_{1}^{i-1}s_{2}^{-i+k+1}b_{i\;-i+k+1}\right)\left({\left(\sum_{i=0}^{k+1}\left(-i+k+1\right)s_{1}^{i}s_{2}^{k-i}a_{i\;-i+k+1}\right)}^{2}\right.\\ \left.+{\left(\sum_{i=0}^{k+1}\left(-i+k+1\right)s_{1}^{i}s_{2}^{k-i}b_{i\;-i+k+1}\right)}^{2}\right)\sum_{j=0}^{k}\sum_{i=0}^{j}is_{1}^{i-1}s_{2}^{j-i}b_{i\;j-i}\\ +2\left(\sum_{i=0}^{k+1}\left(-i+k+1\right)s_{1}^{i}s_{2}^{k-i}a_{i\;-i+k+1}\right)\left(\sum_{j=0}^{k}\sum_{i=0}^{j}\left(j-i\right)s_{1}^{i}s_{2}^{-i+j-1}a_{i\;j-i}\right)\\ \times \left[{\left(\sum_{j=0}^{k}\sum_{i=0}^{j}is_{1}^{i-1}s_{2}^{j-i}a_{i\;j-i}\right)}^{2}+{\left(\sum_{j=0}^{k}\sum_{i=0}^{j}is_{1}^{i-1}s_{2}^{j-i}b_{i\;j-i}\right)}^{2}\right]\\ +2\left(\sum_{i=0}^{k+1}\left(-i+k+1\right)s_{1}^{i}s_{2}^{k-i}a_{i\;-i+k+1}\right)\left({\left(\sum_{i=0}^{k+1}is_{1}^{i-1}s_{2}^{-i+k+1}a_{i\;-i+k+1}\right)}^{2}\right.\\ \left.+{\left(\sum_{i=0}^{k+1}is_{1}^{i-1}s_{2}^{-i+k+1}b_{i\;-i+k+1}\right)}^{2}\right)\sum_{j=0}^{k}\sum_{i=0}^{j}\left(j-i\right)s_{1}^{i}s_{2}^{-i+j-1}a_{i\;j-i}\\ +4(\;\sum_{i=0}^{k+1}\left(-i+k+1\right)s_{1}^{i}s_{2}^{k-i}a_{i\;-i+k+1})(\;\sum_{i=0}^{k+1}is_{1}^{i-1}s_{2}^{-i+k+1}a_{i\;-i+k+1})\\ \times (\;\sum_{j=0}^{k}\sum_{i=0}^{j}\left(j-i\right)s_{1}^{i}s_{2}^{-i+j-1}a_{i\;j-i})\sum_{j=0}^{k}\sum_{i=0}^{j}is_{1}^{i-1}s_{2}^{j-i}a_{i\;j-i}\\ +4(\;\sum_{i=0}^{k+1}\left(-i+k+1\right)s_{1}^{i}s_{2}^{k-i}a_{i\;-i+k+1})(\sum_{i=0}^{k+1}is_{1}^{i-1}s_{2}^{-i+k+1}b_{i\;-i+k+1})\\ \times (\;\sum_{j=0}^{k}\sum_{i=0}^{j}\left(j-i\right)s_{1}^{i}s_{2}^{-i+j-1}a_{i\;j-i})\sum_{j=0}^{k}\sum_{i=0}^{j}is_{1}^{i-1}s_{2}^{j-i}b_{i\;j-i}\\ +2(\;\sum_{i=0}^{k+1}\left(-i+k+1\right)s_{1}^{i}s_{2}^{k-i}b_{i\;-i+k+1})(\;\sum_{j=0}^{k}\sum_{i=0}^{j}\left(j-i\right)s_{1}^{i}s_{2}^{-i+j-1}b_{i\;j-i})\\ \times [(\;\sum_{j=0}^{k}\sum_{i=0}^{j}is_{1}^{i-1}s_{2}^{j-i}a_{i\;j-i}{)}^{2}+(\;\sum_{j=0}^{k}\sum_{i=0}^{j}is_{1}^{i-1}s_{2}^{j-i}b_{i\;j-i}{)}^{2}]\\ +2\left(\sum_{i=0}^{k+1}\left(-i+k+1\right)s_{1}^{i}s_{2}^{k-i}b_{i\;-i+k+1}\right)\left({\left(\sum_{i=0}^{k+1}is_{1}^{i-1}s_{2}^{-i+k+1}a_{i\;-i+k+1}\right)}^{2}\right.\\ \left.+{\left(\sum_{i=0}^{k+1}is_{1}^{i-1}s_{2}^{-i+k+1}b_{i\;-i+k+1}\right)}^{2}\right)\sum_{j=0}^{k}\sum_{i=0}^{j}\left(j-i\right)s_{1}^{i}s_{2}^{-i+j-1}b_{i\;j-i}\\ +4(\;\sum_{i=0}^{k+1}is_{1}^{i-1}s_{2}^{-i+k+1}a_{i\;-i+k+1})(\;\sum_{i=0}^{k+1}\left(-i+k+1\right)s_{1}^{i}s_{2}^{k-i}b_{i\;-i+k+1})\\ \times (\;\sum_{j=0}^{k}\sum_{i=0}^{j}is_{1}^{i-1}s_{2}^{j-i}a_{i\;j-i})\sum_{j=0}^{k}\sum_{i=0}^{j}\left(j-i\right)s_{1}^{i}s_{2}^{-i+j-1}b_{i\;j-i}\\ +4(\;\sum_{i=0}^{k+1}\left(-i+k+1\right)s_{1}^{i}s_{2}^{k-i}b_{i\;-i+k+1})(\;\sum_{i=0}^{k+1}is_{1}^{i-1}s_{2}^{-i+k+1}b_{i\;-i+k+1})\\ \times (\;\sum_{j=0}^{k}\sum_{i=0}^{j}\left(j-i\right)s_{1}^{i}s_{2}^{-i+j-1}b_{i\;j-i})\sum_{j=0}^{k}\sum_{i=0}^{j}is_{1}^{i-1}s_{2}^{j-i}b_{i\;j-i}\\ =\sum_{j=0}^{4k-4}\sum_{i=0}^{-j+4k-4}s_{2}^{-j+4k-4}c_{-i-j+4k-4\;\;i}{\left(\frac{s_{1}}{s_{2}}\right)}^{-i-j+4k-4}\;\;\;\;\;\;\;\;\;\;\;\;\left(\mathrm{by}\;\;S\left(k\right)\right)\\ +[\sum_{i=0}^{4k-3}s_{2}^{4k-3}c_{-i+4k-3\;\;i}{\left(\frac{s_{1}}{s_{2}}\right)}^{-i+4k-3}+\sum_{i=0}^{4k-2}s_{2}^{4k-2}c_{-i+4k-2\;\;i}{\left(\frac{s_{1}}{s_{2}}\right)}^{-i+4k-2}\\ +\sum_{i=0}^{4k-1}s_{2}^{4k-1}c_{-i+4k-1\;\;i}{\left(\frac{s_{1}}{s_{2}}\right)}^{-i+4k-1}+\sum_{i=0}^{4k}s_{2}^{4k}c_{4k-i\;\;i}{\left(\frac{s_{1}}{s_{2}}\right)}^{4k-i}]\\ =\sum_{j=0}^{4k}\sum_{i=0}^{4k-j}s_{2}^{4k-j}c_{-i-j+4k\;\;i}{\left(\frac{s_{1}}{s_{2}}\right)}^{-i-j+4k},\;\;\;\;\;\;\;\;\;\;\;\;\;\;\;\;\;\; \end{array}$$

which agrees with the right-hand side of S(k+1) by virtue of the well-known recursive definition [115,116].

This completes the inductive step S(k)→S(k+1), and so the proof is complete. Therefore, by the principle of mathematical induction, for all n≥2, S(n) is true. □

This lemma, together with the condition that $\Upsilon_{n}\left(s_{1},s_{2}\right)=0$ at the singularities of the magnitude of scalar curvature, then informs us that Equation (108) reduces to

(116) $$\Upsilon_{n}\left(s_{1},s_{2}\right)=\sum_{j=0}^{4(n-1)}s_{2}^{4(n-1)-j}\Psi_{4(n-1)-j}\left(\frac{s_{1}}{s_{2}}\right)=0,$$

where, according to Lemma 3 above,

(117) $$\Psi_{4(n-1)-j}\left(\frac{s_{1}}{s_{2}}\right)=\sum_{i=0}^{4(n-1)-j}{\left(\frac{s_{1}}{s_{2}}\right)}^{-i-j+4(n-1)}c_{-i-j+4(n-1)\;\;i},$$

is a polynomial in $s_{1}/s_{2}$ of degree 4(n−1)−j, in which

(118) $$c_{-i-j+4(n-1)\;\;i}=\frac{1}{i!\left(-i-j+4(n-1)\right)!}\left(\frac{\partial^{-i-j+4(n-1)}}{\partial s_{1}^{-i-j+4(n-1)}}\frac{\partial^{i}}{\partial s_{2}^{i}}\Upsilon_{n}\left(s_{1},s_{2}\right)\right)\;\mathrm{at}\;\left(s_{1},s_{2}\right)=\left(0,0\right).$$

The motivation for constructing Equation (116) was that it forms, in terms of homogeneous polynomials, a more valuable expression to determine the maximum number of singularities of scalar curvature, for which the method of geometric characteristics is capable of providing an exact solution. Finally, it should be observed that the procedure adopted above to simplify the denominator equation of R using mathematical induction is not a new idea here. In fact, it has various applications, particularly in simplifying products of power sums. For more details, see chapter 9 of Ref. [116].

As outlined in the Appendix A, the problem of determining the singular points of |R| coincides with that of finding the intersection points of algebraic curves with their asymptotes. As we may recognize, Equation (116) is an algebraic curve of degree 4(n−1) as a consequence of the boundary condition that $\Upsilon_{n}\left(s_{1},s_{2}\right)=0$ at $p_{i}^{C}$. Hence, the number of common points of the new expression of the denominator of the scalar curvature and its asymptotes must be equal to the number of solutions of Equation (116) by virtue of Bézout’s theorem. Therefore, in order to determine the total number of solutions of Equation (116), we carry out the program of the method of geometric characteristics mentioned in Appendix A. For this, we start by working forward from the hypothesis that

(119) $$s_{1}=\kappa s_{2}+\sigma$$

is an asymptote of Equation (116), where the angular and linear coefficients, κ and σ, respectively, are finite. From the method of geometric characteristics, we must substitute Equation (119) into Equation (116) to determine κ and σ. As a result, we then encounter

(120) $$\begin{array}{c} \sum_{j=0}^{4(n-1)}s_{2}^{4(n-1)-j}\Psi_{4(n-1)-j}\left(\kappa +\frac{\sigma }{s_{2}}\right)=s_{2}^{4(n-1)}\Psi_{4(n-1)}\left(\kappa +\frac{\sigma }{s_{2}}\right)+s_{2}^{4(n-1)-1}\Psi_{4(n-1)-1}\left(\kappa +\frac{\sigma }{s_{2}}\right)\\ +s_{2}^{4(n-1)-2}\Psi_{4(n-1)-2}\left(\kappa +\frac{\sigma }{s_{2}}\right)+\cdots +s_{2}^{0}\Psi_{0}\left(\kappa +\frac{\sigma }{s_{2}}\right)=0. \end{array}$$

We may now employ Taylor’s series expansion theorem to deduce that

(121) $$\begin{array}{cc} s_{2}^{4(n-1)}\Psi_{4(n-1)}\left(\kappa \right) & +s_{2}^{4(n-1)-1}\left[\Psi_{4(n-1)-1}\left(\kappa \right)+\sigma \frac{d\Psi_{4(n-1)}\left(\kappa \right)}{ds_{2}}\right]\\ & +s_{2}^{4(n-1)-2}\left[\left(\frac{\sigma^{2}}{2!}\right)\frac{d^{2}\Psi_{4(n-1)}\left(\kappa \right)}{ds_{2}^{2}}+\sigma \frac{d\Psi_{4(n-1)-1}\left(\kappa \right)}{ds_{2}}+\Psi_{4(n-1)-2}\left(\kappa \right)\right]+\ldots =0. \end{array}$$

According to the method of geometric characteristics, if Equation (119) is an asymptote of Equation (116), then the values of κ and σ are given by

(122) $$\Psi_{4(n-1)}\left(\kappa \right)=0,$$

and

(123) $$\begin{array}{c} \sigma_{i}=-\left[\frac{\Psi_{4(n-1)-1}\left(\kappa \right)}{\frac{d\Psi_{4(n-1)}\left(\kappa \right)}{ds_{2}}}\right]\;\mathrm{at}\;\kappa =\kappa_{i}\;\mathrm{with}\;i=1,2,3,\ldots ,4\left(n-1\right)\;, \end{array}$$

in which it is understood that the real part of the solutions of Equations (122) and (123) is to be taken by virtue of the method of geometric characteristics.

Before continuing, it will prove useful to determine the maximum number of asymptotes related to Equation (108) from the knowledge of Equation (122). To this end, we make use of the following lemma:

**Lemma** **4.** *If a and b are real coefficients with the property that Equation (122) has only complex conjugate pairs of roots, then Equation (108), or alternatively, Equation (116), has the maximum number of 2(n−1) asymptotes.*

**Proof.**  It should be noted that an algebraic curve of degree *n* cannot have more than *n* asymptotes, as outlined in the Appendix A. Nevertheless, one must resist the impulse to conclude that Equation (116) would lead 4(n−1) asymptotes by virtue of the nature of the solutions of Equation (122). So, to prove this lemma, let us return to Equation (108), displayed in the form

(124) $$\begin{array}{c} \Upsilon_{n}\left(s_{1},s_{2}\right)=[(\;\sum_{j=0}^{n}\sum_{i=0}^{j}is_{1}^{i-1}s_{2}^{j-i}a_{i,j-i}{)}^{2}+(\;\sum_{j=0}^{n}\sum_{i=0}^{j}is_{1}^{i-1}s_{2}^{j-i}b_{i,j-i}{)}^{2}]\\ \times [(\;\sum_{j=0}^{n}\sum_{i=0}^{j}s_{1}^{i}\left(js_{2}^{-i+j-1}-is_{2}^{-i+j-1}\right)a_{i,j-i}{)}^{2}+(\;\sum_{j=0}^{n}\sum_{i=0}^{j}s_{1}^{i}\left(js_{2}^{-i+j-1}-is_{2}^{-i+j-1}\right)b_{i,j-i}{)}^{2}]=0. \end{array}$$

The equation of $\Upsilon_{n}\left(s_{1},s_{2}\right)$ presented earlier differs from Equation (124) only by the development of the derivatives of $s_{1}$ and $s_{2}$ involved in Equation (108). As one can readily see, the denominator of R is a positive multivariable polynomial equation since it is a product of polynomial expressions squared. Consequently, we then are led to conclude that Equation (122) is a univariate positive polynomial equation of degree 4(n−1) as a result of the positivity of Equation (108), or Equation (116), and the method of geometric characteristics. From Descartes’ sign rule [77], however, it then naturally follows that Equations (122), and consequently, Equation (123), cannot have real solutions, i.e., the polynomial equations corresponding to the angular and linear coefficients can have only 4(n−1) pairs of complex conjugate roots.

In accordance with the method of geometric characteristics, the solutions of the polynomial equations of the angular and linear coefficients cannot be imaginary because only the real part of those coefficients is significant in the framework of GBT. In this latter situation, therefore, the real parts of the solutions for the angular and linear coefficients are to be extracted. Hence, instead of 4(n−1) complex conjugate pairs of roots, Equation (122) has 2(n−1) real roots, and hence, Equation (108), or alternatively, Equation (116), has the maximum number of 2(n−1) asymptotes, thus completing the proof. □

To conclude, by virtue of Descartes’ sign rule and the positivity of Equation (108), it is evident that Equation (122) has only complex conjugate pairs of roots. Nonetheless, because the method of geometric characteristics states that the real parts of the complex solutions for Equations (122) and (123) should be extracted, since only those are significant from the viewpoint of GBT, it then follows that Equation (122) has 2(n−1) real roots, and therefore, Equation (108), or alternatively, Equation (116), has the maximum number of 2(n−1) asymptotes, which was the result to be demonstrated.

Based on the above, Equation (121) then reduces to

(125) $$s_{2}^{4(n-1)-2}\left[\left(\frac{\sigma^{2}}{2!}\right)\frac{d^{2}\Psi_{4(n-1)}\left(\kappa \right)}{ds_{2}^{2}}+\sigma \frac{d\Psi_{4(n-1)-1}\left(\kappa \right)}{ds_{2}}+\Psi_{4(n-1)-2}\left(\kappa \right)\right]+\ldots =0,$$

from which it follows that Equation (125) is a polynomial of degree 4(n−1)−2, and consequently, yields 4(n−1)−2 values of $s_{2}$. Consequently, Equation (125) teaches us that the inclined asymptote of Equation (119) intersects the algebraic curve of Equation (116) in (4(n−1)−2) points.

From the method of geometric characteristics, the problem of finding the singularities of the magnitude of scalar curvature is equivalent to determining the intersection points of Equation (116) with its inclined asymptotes. On account of the fact that an oblique asymptote, Equation (119), intersects the algebraic curve of Equation (116) in (4(n−1)−2) points and the number of asymptotes of Equation (116) is 2(n−1) by virtue of Lemma 4, it then follows that the maximum number of singularities of Equation (101) is, therefore, equal to 2(n−1)(4(n−1)−2), which was the result to be demonstrated.

From the hypothesis that the dynamical system under consideration is a polynomial system of degree *n* with no common linear factors, we have proved that the scalar curvature associated with Equation (99) is a positive multivariable function under the necessary condition that $a_{10}b_{01}>b_{10}a_{01}$. In addition, we have demonstrated that the magnitude of R diverges to infinity at 2(n−1)(4(n−1)−2) real singular points. From the definition of limit cycles in the framework of GBT, the total number of distinct divergences of the magnitude of R to infinity gives the maximum number of limit cycles, with their relative positions being determined by the singularities of |R|.

As outlined above, the problem of determining the singular points of the magnitude of scalar curvature R corresponds to finding the intersection points of an algebraic curve with its asymptotes. As we may recognize, Equation (116) is an algebraic curve of degree 4(n−1) as a consequence of the boundary condition that $\Upsilon_{n}\left(s_{1},s_{2}\right)=0$ at $p_{i}^{C}$. Hence, the number of common points of the denominator of R and its asymptotes must be equal to the number of solutions of Equation (116) by virtue of Bézout’s theorem.

On account of the fact that the scalar curvature of the dissipative polynomial system of degree n≥2 Equation (99) is positive, and the magnitude of R diverges to infinity at 2(n−1)(4(n−1)−2) real singularities, it then follows, together with the covariant definition of limit cycles, that the dynamical system under consideration, Equation (99), has the maximum number of 2(n−1)(4(n−1)−2) limit cycles whose relative positions are the critical points in which the magnitude of scalar curvature becomes singular, thus establishing the conclusion and completing the proof. □

Hence, we may conclude that the covariant formulation of GBT unambiguously provides the maximum number and relative positions of limit cycles of *n*th-degree polynomial systems. In addition, this result naturally implies that a vector field in the plane has only a finite number of limit cycles, which is in remarkable agreement with the corollaries of the non-accumulative theorem [117,118,119].

In terms of the above discussion, we close this section by constructing a new definition for the maximum number of limit cycles of *n*th-degree polynomial systems, which at times is called the Hilbert number. It should be remarked from previous sections that the number of zeroes of the denominator of the scalar curvature depends directly on the real constant coefficients $a_{ij}$ and $b_{ij}$ of the polynomial system of *n*th degree.

Bearing this in mind, let $H_{n}\left(a,b\right)$ denote the number of limit cycles of the *n*th-degree polynomial system Equation (99), with real constant coefficients (a,b) such that (a,b) $\in R^{\left(n+1)(n+2\right)}$. In light of Theorem 4, an alternative definition for Hilbert’s number can now be formally defined by

(126) $$\begin{array}{cc} H_{n}\left(a,b\right) & ={\mathrm{Sup}}_{n\in N}\left\{\pi \left(R^{-1}\right):\deg R^{-1}\leq 4\left(n-1\right)\right\}\\ & =2(n-1)(4(n-1)-2) \end{array}$$

for all $\left(a,b\right)\in R^{\left(n+1)(n+2\right)}$. Here, $\pi (R^{-1})$ denotes the number of zeroes of the denominator of scalar curvature $R(s_{1},s_{2})$, while deg means “the degree of”. In writing $R^{-1}$, we mean 1/R rather than the reciprocal of R.

As a final remark, it is evident that Theorem 4, along with the new definition for the number of Hilbert, is in remarkable agreement with the results obtained some time ago by other authors and in somewhat different contexts [6,117,118,119].

To conclude, we have, so far, developed and extended many features of the covariant formulation of GBT to investigate isolated periodic trajectories of dynamical systems in the framework of differential equations. In the foregoing, it is verified that the covariant formulation of GBT can unambiguously account for the maximum number and position of limit cycles of dynamical systems in the framework of differential equations. As a result, this provides a successful solution to the sixteenth challenge of Hilbert.

## 5. Conclusions

In the present paper, the covariant formulation of GBT has been developed to provide an affirmative answer to the elementary problem of the maximum number and relative positions of isolated periodic trajectories of dynamical systems in the framework of differential equations, which is known as the second half of the sixteenth challenge of Hilbert.

As outlined previously, attempts to predict both the number and position of limit cycles from the perspective of CBT have been beset by several difficulties, attributable in most part to falling results and their lack of consistency. Moreover, the standard methods usually employed in the study of limit cycles have several drawbacks, since they only provide clues about the location of the possible limit cycles in the phase portraits.

Despite the contradictory results on the understanding of Hilbert’s sixteenth challenge, it has been pointed out that the application of invariant theories and geometric methods for the investigation of dynamical systems could not only provide an alternative perspective to construct a complete solution to this challenge but also contribute to solving other open problems of qualitative theory of differential equations. Therefore, this suggests that CBT unquestionably requires revision, and it would be desirable to incorporate those aspects into a new theoretical description and investigation of dynamical systems.

This goal has been achieved by a new covariant and geometric formulation of bifurcation theory without introducing fundamentally new concepts. In the geometric bifurcation theory, a Riemannian metrical and invariant structure of the parameter space is introduced and investigated for dynamical systems in the framework of differential equations. Consequently, we have applied the covariant formulation of GBT to study the behavior of the curvature R for the elementary problem of limit cycles. With our aim of providing a satisfactory answer to the sixteenth challenge of Hilbert, it became necessary to construct a proper and suitable covariant definition for limit cycles in the framework of GBT.

As a result of several theoretical improvements and extensions, along with numerical results for various circumstances involving dynamical systems with different numbers of isolated periodic trajectories, we could covariantly define limit cycles from the standpoint of GBT to be that periodic state in which the scalar curvature is positive in the neighborhood of the critical points of the dynamical system and whose magnitude, |R|, diverges to infinity at its singular points.

Precisely, a two-dimensional dissipative system with only one limit cycle is that in which the scalar curvature is positive and whose magnitude diverges to infinity at singular points symmetrical with respect to the origin. Alternatively, two-dimensional dissipative systems with more than one limit cycle are understood to be those in which the curvature R is positive, and the magnitude of R diverges to infinity at non-symmetrical singularities. Consequently, we are led to conclude that the total number of divergences of |R| to infinity gives the total number of limit cycles in the phase portrait of the dynamic system under study. Finally, if R≤0, then the two-dimensional dissipative system under consideration has no limit cycles, according to the geometrical viewpoint of the sign of the curvature scalar.

With the covariant definition of limit cycles in the framework of GBT, we then dedicated ourselves to the statements of the second half of Hilbert’s sixteenth challenge.

The first problem to which we turned our attention, however, was that of the maximum number of limit cycles of second-degree polynomial systems. It was also confirmed that R is a positive multivariable function under the necessary condition that $a_{10}b_{01}>b_{10}a_{01}$ for a real polynomial system of degree two with no common linear factors. Additionally, we have demonstrated that the magnitude of R diverges to infinity at four real singular points. Because the scalar curvature is positive and |R| diverges to infinity at four singular points, it naturally follows, together with the covariant definition of limit cycles, that quadratic polynomial systems have the maximum number of four limit cycles.

Having derived the foundations of the geometric bifurcation theory, we finally devoted ourselves to the problem of the number and relative positions of limit cycles for *n*th-degree polynomial systems, which is the second part of Hilbert’s sixteenth problem.

From the hypothesis that the dynamical system under consideration is a real polynomial system of degree *n* with no common linear factors and the necessary condition that $a_{10}b_{01}>b_{10}a_{01}$, we demonstrated that the scalar curvature associated with Equation (99) is a positive multivariable function. Furthermore, we also proved that the magnitude of R diverges to infinity at 2(n−1)(4(n−1)−2) real singular points.

On account of the fact that the scalar curvature of polynomial systems of degree n≥2, Equation (99), is positive and the magnitude of R diverges to infinity at 2(n−1)(4(n−1)−2) real singular points, it then follows, together with the definition of limit cycles, that real polynomial systems of degree *n* with no common linear factors have the maximum number of 2(n−1)(4(n−1)−2) limit cycles, with their relative positions given by the singular points of |R|.

It should be remarked that the conclusions thus obtained throughout this work for polynomial differential equations of degree n≥2 reveal that a vector field in the plane has only a finite number of limit cycles from the standpoint of GBT, which is in remarkable agreement with the non-accumulative theorem.

In light of the aforementioned, we are led to conclude that the covariant formulation of GBT and the method of geometric characteristics can unambiguously account for the maximum number and relative positions of limit cycles for dynamical systems governed by polynomial differential equations. As a natural consequence of the development of those geometrical methods, we have demonstrated that *n*th-degree polynomial systems have the maximum number of 2(n−1)(4(n−1)−2) limit cycles, with their relative positions being determined by the critical points in which the magnitude of R becomes singular. In view of the results found, this provides the proper successful solution to the second half of Hilbert’s sixteenth problem. Furthermore, the geometrical methods introduced here add a new theoretical possibility to analytically investigate limit cycles that, we believe, may be an avenue for future developments of nonlinear dynamics.

## Figures and Tables

**Table 1:** Shows the notation for symbols appearing in more than one place in this paper. Because of the large number of symbols in GBT, we may occasionally use the same symbol for different quantities. The usage, however, should be clear from the context.

Notation	Meaning
X,Y	Rational integral functions
x,y	Real variables
*r*	Dimension of the parameter space *X*
τ	Time
$\beta_{i}$	Momenta
$s_{i}$	Order parameters
$m_{i}$	Control parameters
$p_{i}\left(s_{1}^{*},s_{2}^{*}\right)\equiv p_{i}$	Equilibrium points
$p_{i}^{C}\left(s_{1}^{*},s_{2}^{*}\right)\equiv p_{i}^{C}$	Critical/singular points of R
Det	Determinant
J	Jacobian matrix
$\rho \left(\beta_{1},\beta_{2}\right)=\rho \left(\beta \right)$	Generalized Gaussian probability density
*T*	Period of time
Ω	Family of probability distributions
*X*	Parameter space
M	Riemannian manifold
$G_{\alpha \;\mu }\left(X\right)$	Fisher covariant symmetric tensor
$G_{\alpha \;\mu }$	Metric elements
*G*	Determinant of $G_{\alpha \;\mu }\left(X\right)$
$R_{\xi \;\eta \;l}^{\alpha }$	Fourth-rank Riemannian curvature tensor
R	Scalar curvature
|R|	Magnitude of scalar curvature
$\varphi (s_{1},s_{2})$	Dulac function
$I_{i}=I_{i}\left(p_{i}\right)$	Index of M
χ(M)	Euler–Poincaré characteristic of M
Tr	Trace
Q	Saddle value/quantity
$\Lambda_{i}$	Eigenvalues
Res(.)	Resultant

**Table 2:** Shows the behavior of the scalar curvature R for dynamical systems described by several bifurcations. Here, we give the dimension of the parameter space (*r*), sign of R, local structural stability, and the divergence of R, in which the sign convention of Weinberg is understood [25,35,36].

Bifurcations	*r*	R Sign	Local Structural Stability	Divergence
Saddle-node [25]	2	−	Unstable	Bifurcation point
Transcritical [25]	2	−	Unstable	Bifurcation point
Supercritical pitchfork [25]	2	+/−	Stable/unstable	Bifurcation point
Subcritical pitchfork [25]	2	−/+	Unstable/stable	Bifurcation point
Homoclinic [25] (unperturbed oscillator)	3	+	Stable	Bifurcation point
Homoclinic [25] (perturbed oscillator)	3	+	Stable	Bifurcation point
Saddle-node [26] (2D Kuramoto model)	2	−	Unstable	Bifurcation point

## Data Availability

Data are contained within the article.

## Appendix A. The Method of Geometric Characteristics

In this section, we discuss a method for obtaining rigorous singularities of the magnitude of scalar curvature. The techniques employed here are most commonly seen in algebraic geometry, but we will see that the concepts prove useful in the study of limit cycles from the standpoint of GBT as well.

As we saw in great detail in Section 2, the positive definite and geometrically invariant Riemannian metric

(A1) $$d\ell^{2}=G_{11}{\left(ds_{1}\right)}^{2}+G_{22}{\left(ds_{2}\right)}^{2}$$

defines a fourth-rank Riemannian curvature tensor $R_{\xi \;\eta \;l}^{\alpha }$ in terms of the derivatives of the nonlinear functions $\Phi_{1}$ and $\Phi_{2}$. The full contraction of $R_{\xi \;\eta \;l}^{\alpha }$ yields the scalar curvature R, which is a rational function expressed as follows,

(A2) $$R=\frac{Z(s_{1},s_{2})}{W(s_{1},s_{2})},$$

where we may assume with no loss of generality that

(A3) $$Z\left(s_{1},s_{2}\right)=Z_{n}\left(s_{1},s_{2}\right)+Z_{n-1}\left(s_{1},s_{2}\right)+\ldots +Z_{1}\left(s_{1},s_{2}\right)+Z_{0},$$

where

$$\begin{array}{cc} Z_{n}\left(s_{1},s_{2}\right) & =\sum_{i+j=0}^{n}e_{ij}s_{1}^{i}s_{2}^{j},\\ Z_{n-1}\left(s_{1},s_{2}\right) & =\sum_{\nu +r=0}^{n-1}b_{\nu r}s_{1}^{\nu }s_{2}^{r},\;\ldots \end{array}$$

and

(A4) $$W\left(s_{1},s_{2}\right)=W_{n}\left(s_{1},s_{2}\right)+W_{n-1}\left(s_{1},s_{2}\right)+\ldots +W_{1}\left(s_{1},s_{2}\right)+W_{0},$$

whence,

$$\begin{array}{cc} W_{n}\left(s_{1},s_{2}\right) & =\sum_{i+j=0}^{n}h_{ij}s_{1}^{i}s_{2}^{j},\\ W_{n-1}\left(s_{1},s_{2}\right) & =\sum_{\nu +r=0}^{n-1}b_{\nu r}s_{1}^{\nu }s_{2}^{r},\ldots \end{array}$$

In the framework of GBT, we define a limit cycle as the periodic state where the scalar curvature R is positive and whose magnitude, |R|, diverges to infinity at non-symmetrical singularities. Another way of expressing the latter statement is to impose that $W(s_{1},s_{2})$ should vanish at the critical points of the magnitude of scalar curvature because |R| is continuous except at $p_{i}^{C}$ where the denominator $W(s_{1},s_{2})$ is zero. Consequently, in order to determine the critical points $p_{i}^{C}$ such that the magnitude of R diverges to infinity, one must now seek the exact solutions of the homogeneous polynomial equation

(A5) $$W(s_{1},s_{2})=0,$$

which naturally poses a challenge since the exact solutions of algebraic equations, such as Equation (A5), or roots of polynomial expressions, Equation (A4), are difficult to obtain.

To circumvent this difficulty, however, we now present and illustrate the method of geometric characteristics, which provides a general approach to constructing singularities of the magnitude of scalar curvature. To that end, we first exploit the connection between the curvature R and the asymptotes of *n*th-degree algebraic curves by presenting a deeper level of analysis of R from the viewpoint of the algebraic geometry of curves and asymptotes of rational functions.

Let $A(s_{1},s_{2})$ be a polynomial written in groups of homogeneous terms in $s_{1}$ and $s_{2}$ of the form

(A6) $$A_{n}\left(s_{1},s_{2}\right)+A_{n-1}\left(s_{1},s_{2}\right)+\ldots +A_{1}\left(s_{1},s_{2}\right)+A_{0}=0,$$

where

(A7) $$A_{n}\left(s_{1},s_{2}\right)=\sum_{i+j=0}^{n}a_{ij}s_{1}^{i}s_{2}^{j},\;\ldots$$

The set of points $\left(s_{1},s_{2}\right)$ in the plane such that $A(s_{1},s_{2})=0$ forms an algebraic curve γ of order *n*. Another way of presenting this plane algebraic curve is

(A8) $$A\left(s_{1},s_{2}\right)=\sum_{j=0}^{n}s_{2}^{n-j}H_{n-j}\left(\frac{s_{1}}{s_{2}}\right)=0,$$

where $H_{n-j}\left(s_{1}/s_{2}\right)$ is a polynomial in $s_{1}/s_{2}$ of degree n−j.

As an example of Equation (A6), or alternatively, Equation (A8), one has the algebraic curves of degree one

(A9) $$a_{00}+s_{2}a_{01}+s_{1}a_{10}=0,$$

which denote straight lines. Conversely, algebraic curves of degree two,

(A10) $$a_{00}+s_{2}a_{01}+s_{2}^{2}a_{02}+s_{1}a_{10}+s_{1}s_{2}a_{11}+s_{1}^{2}a_{20}=0,$$

are conic sections [120,121,122,123,124].

Bearing this in mind, we then recognize that

(A11) $$W\left(s_{1},s_{2}\right)=W_{n}\left(s_{1},s_{2}\right)+W_{n-1}\left(s_{1},s_{2}\right)+\ldots +W_{1}\left(s_{1},s_{2}\right)+W_{0}=0,$$

denotes, in fact, an algebraic plane curve as a natural consequence of the restriction that imposes $W(s_{1},s_{2})=0$ at the singularities of the magnitude of scalar curvature. In connection with the latter conclusion, it should be remarked that Equation (A11), being an algebraic curve, can therefore have at most, i.e., no more than, *n* oblique asymptotes of the form

(A12) $$s_{1}-\kappa s_{2}-\sigma =0,$$

which is essentially a straight line or an algebraic curve of degree one, where κ and σ are the angular and linear coefficients, respectively. With the aid of the theorem of Bézout, which is a generalization of the fundamental theorem of algebra, and the theory of resultants of polynomial equations, the number of common points of Equation (A11) and its correspondent asymptotes is equal to the number of real solutions of Equation (A11). This comprehension is more general than the original description of the problem and certainly enlarges our perspective about the curvature R since we then are led to conclude that the problem of finding the singularities of the magnitude of scalar curvature is, therefore, equivalent to determining the intersection points of Equation (A11) with its inclined asymptotes [106,107,108,109,110,111,112,113,114].

With this in mind, the method of geometric characteristics, which we shall use to construct the singularities of the magnitude of scalar curvature, is the following. From the computation of scalar curvature, we first impose the condition that its denominator must vanish at the singularities of the magnitude of R. Second, we then rewrite the denominator equation of R as a product of polynomial factors, where both are set equal to zero as a natural consequence of the previous condition. From the knowledge of the resulting polynomial equations, we devote ourselves to their asymptotes. Finally, we obtain the singularities of the magnitude of scalar curvature by determining the intersection points of the polynomial equations associated with each factor of the denominator equation of R with their corresponding asymptotes by employing the resultant theory.

Before proceeding with the practical illustration of the method, it is helpful to explain first the general procedure, with a revised account of its mathematical techniques.

Following the program outlined above, we start by deriving the scalar curvature, Equation (A2), and consequently, we impose the condition that its denominator must vanish at the singularities of the magnitude of R, which leads us to Equation (A11).

The next stage is reached by writing the denominator equation of R as a product of polynomial factors. In view of Equation (8) and the previous results found in Section 3, however, it is not difficult to verify that the denominator of R is already written in terms of a product of two polynomial factors. With no loss of generality, we then may assume that the denominator equation for R could be alternatively expressed as follows:

(A13) $$W\left(s_{1},s_{2}\right)=M\left(s_{1},s_{2}\right)U\left(s_{1},s_{2}\right)=0,$$

whence,

(A14) $$\left\{\begin{array}{cc} M(s_{1},s_{2}) & =\sum_{i+j=0}^{n}{\tilde{q}}_{ij}s_{1}^{i}s_{2}^{j},\\ U(s_{1},s_{2}) & =\sum_{i+j=0}^{m}{\tilde{k}}_{ij}s_{1}^{i}s_{2}^{j}. \end{array}\right.$$

Although the same letter is used, there shouldn’t be any danger of confusing this *m* with the control parameter in Section 2.

To complete the second stage of our program, let us look back at Equation (A13). As outlined above, $M(s_{1},s_{2})$ and $U(s_{1},s_{2})$ are factors of the polynomial expression of the denominator of scalar curvature. As a consequence of Equation (A13), and remembering that $M\left(s_{1},s_{2}\right)U\left(s_{1},s_{2}\right)=0$, means one or both $M(s_{1},s_{2})$ and $U(s_{1},s_{2})$ must be zero; we then have

(A15) $$M\left(s_{1},s_{2}\right)=\sum_{i+j=0}^{n}{\tilde{q}}_{ij}s_{1}^{i}s_{2}^{j}=0,$$

and, of course,

(A16) $$U\left(s_{1},s_{2}\right)=\sum_{i+j=0}^{m}{\tilde{k}}_{ij}s_{1}^{i}s_{2}^{j}=0.$$

An alternative representation of the polynomial equations above can be obtained by rewriting Equations (A15) and (A16) as follows:

(A17) $$M\left(s_{1},s_{2}\right)=\sum_{j=0}^{n}s_{2}^{n-j}\Psi_{n-j}\left(\frac{s_{1}}{s_{2}}\right)=0,$$

and

(A18) $$U\left(s_{1},s_{2}\right)=\sum_{j=0}^{m}s_{2}^{m-j}\Theta_{m-j}\left(\frac{s_{1}}{s_{2}}\right)=0,$$

in which $\Psi_{n-j}\left(s_{1}/s_{2}\right)$ and $\Theta_{m-j}\left(s_{1}/s_{2}\right)$ are polynomials expressed in terms of $s_{1}$ and $s_{2}$.

We now proceed to the next stage, in which the asymptotes of $M(s_{1},s_{2})$ and $U(s_{1},s_{2})$ are obtained separately with the aid of series expansions of homogeneous polynomial equations [83,84,85,87,120]. With this purpose in mind, we begin by constructing the asymptotes of Equations (A17).

Let us consider

(A19) $$s_{1}=\kappa s_{2}+\sigma$$

to be the asymptote of Equation (A17) with the property that both κ and σ are real. To proceed further, one must calculate the values of κ and σ. For this, we divide both sides of Equation (A17) by $s_{2}^{n-j}$ and tend $s_{2}$ to infinity. On recalling the definition of asymptotes, one then encounters

(A20) $$\Psi_{n}\left(\kappa \right)=0,$$

which is a polynomial expressed in terms of κ. This fact basically teaches us that the solutions of Equation (A20), of which only the real part is significant, provide the values of the possible angular coefficients of Equation (A19).

Having obtained the angular coefficient κ by solving Equation (A19), we now dedicate ourselves to the computation of the possible values of linear coefficients σ. To that end, let us substitute Equation (A19) into Equation (A17), from which we obtain

(A21) $$\begin{array}{cc} M\left(\kappa s_{2}+\sigma ,s_{2}\right)=M\left(s_{2}\right) & =\sum_{j=0}^{n}s_{2}^{n-j}\Psi_{n-j}\left(\kappa +\frac{\sigma }{s_{2}}\right)\\ & =s_{2}^{n}\Psi_{n}\left(\kappa +\frac{\sigma }{s_{2}}\right)+s_{2}^{n-1}\Psi_{n-1}\left(\kappa +\frac{\sigma }{s_{2}}\right)\\ & +s_{2}^{n-2}\Psi_{n-2}\left(\kappa +\frac{\sigma }{s_{2}}\right)+\cdots +s_{2}^{0}\Psi_{0}\left(\kappa +\frac{\sigma }{s_{2}}\right)=0, \end{array}$$

where Equation (A17) becomes a polynomial written in terms of $s_{2}$. The decisive step now is to employ the theorem of Taylor to expand $\Psi_{n-j}\left(\kappa +\sigma /s_{2}\right)$ of Equation (A21) in terms of $s_{2}$. As a result, we find

(A22) $$\begin{array}{cc} M(s_{2}) & =\left[\Psi_{n}\left(\kappa \right)+\left(\frac{\sigma }{s_{2}}\right)\frac{d\Psi_{n}\left(\kappa \right)}{ds_{2}}+\frac{1}{2!}{\left(\frac{\sigma }{s_{2}}\right)}^{2}\frac{d^{2}\Psi_{n}\left(\kappa \right)}{ds_{2}^{2}}+\ldots \right]s_{2}^{n}\\ & +\left[\Psi_{n-1}\left(\kappa \right)+\left(\frac{\sigma }{s_{2}}\right)\frac{d\Psi_{n-1}\left(\kappa \right)}{ds_{2}}+\frac{1}{2!}{\left(\frac{\sigma }{s_{2}}\right)}^{2}\frac{d^{2}\Psi_{n-1}\left(\kappa \right)}{ds_{2}^{2}}+\ldots \right]s_{2}^{n-1}\\ & +\left[\Psi_{n-2}\left(\kappa \right)+\left(\frac{\sigma }{s_{2}}\right)\frac{d\Psi_{n-2}\left(\kappa \right)}{ds_{2}}+\frac{1}{2!}{\left(\frac{\sigma }{s_{2}}\right)}^{2}\frac{d^{2}\Psi_{n-2}\left(\kappa \right)}{ds_{2}^{2}}+\ldots \right]s_{2}^{n-2}\\ & +\cdots +s_{2}^{0}\Psi_{0}\left(\kappa +\frac{\sigma }{s_{2}}\right)=0, \end{array}$$

or alternatively,

(A23) $$\begin{array}{cc} s_{2}^{n}\Psi_{n}\left(\kappa \right) & +s_{2}^{n-1}\left[\sigma \frac{d\Psi_{n}\left(\kappa \right)}{ds_{2}}+\Psi_{n-1}\left(\kappa \right)\right]\\ & +s_{2}^{n-2}\left[\left(\frac{\sigma^{2}}{2!}\right)\frac{d^{2}\Psi_{n}\left(\kappa \right)}{ds_{2}^{2}}+\sigma \frac{d\Psi_{n-1}\left(\kappa \right)}{ds_{2}}+\Psi_{n-2}\left(\kappa \right)\right]+\cdots +s_{2}^{0}\Psi_{0}\left(\kappa +\frac{\sigma }{s_{2}}\right)=0. \end{array}$$

From Equation (A20), however, it should be remarked that $\Psi_{n}\left(\kappa \right)=0$. Using this fact, and dividing both sides of Equation (A23) by $s_{2}^{n-1}$, we can then make the following rearrangement:

(A24) $$\begin{array}{cc} \sigma \frac{d\Psi_{n}\left(\kappa \right)}{ds_{2}} & +\Psi_{n-1}\left(\kappa \right)\\ & +\frac{1}{s_{2}}\left[\left(\frac{\sigma^{2}}{2!}\right)\frac{d^{2}\Psi_{n}\left(\kappa \right)}{ds_{2}^{2}}+\sigma \frac{d\Psi_{n-1}\left(\kappa \right)}{ds_{2}}+\Psi_{n-2}\left(\kappa \right)\right]+\cdots +\frac{1}{s_{2}^{n-1}}\left[\Psi_{0}\left(\kappa +\frac{\sigma }{s_{2}}\right)\right]=0. \end{array}$$

By taking the limit $s_{2}\to \infty$, we deduce from Equation (A24) that

(A25) $$\sigma =-\left[\frac{\Psi_{n-1}\left(\kappa \right)}{\left(\frac{d\Psi_{n}\left(\kappa \right)}{ds_{2}}\right)}\right]\;\mathrm{at}\;\kappa =\kappa_{i}\;\mathrm{with}\;i=1,2,3\ldots ,n$$

which provides a general expression for the linear coefficients of Equation (A19). Let $\kappa_{i}$ denote the real and distinct solutions of the homogeneous polynomial $\Psi_{n}\left(\kappa \right)$. In addition, let $\sigma_{i}$ be the corresponding values for the linear coefficients obtained from Equation (A25) by introducing $\kappa_{i}$. Hence, the equations for the *n* oblique asymptotes associated with Equation (A17) may be written explicitly in the form

(A26) $$s_{1}=\sigma_{i}\left[1+\left(\frac{\kappa_{i}}{\sigma_{i}}\right)s_{2}\right],\;i=1,2,3,\ldots ,n$$

of which only the real part is significant. As an alternative representation, it will prove useful to rewrite Equations (A26) implicitly as follows:

(A27) $$N_{i}\left(s_{1},s_{2}\right)=s_{1}-\sigma_{i}\left[1+\left(\frac{\kappa_{i}}{\sigma_{i}}\right)s_{2}\right]=0,\;i=1,2,3,\ldots ,n.$$

From this it naturally follows that Equation (A17) may have *n* asymptotes depending on the solutions of Equation (A20). We should remark, parenthetically, that a more direct approach for the calculation of the linear and angular coefficients, and the one we shall actually employ for the construction of the asymptotes of the denominator equation of the scalar curvature, is obtained by simply substituting $s_{1}$ by $s_{2}\kappa +\sigma$ into Equation (A17) and equating both coefficients of $s_{2}^{n}$ and $s_{2}^{n-1}$ of the resulting expression to zero. Going through some straightforward algebraic simplifications, one immediately encounters Equations (A20) and (A25), from which we may determine the values of κ and σ of the asymptotes associated with Equation (A17).

It must be emphasized that if the coefficients of $s_{2}^{n-1}$ vanish by virtue of any possible value of κ, then the corresponding value of σ must be determined by equating the coefficients of the term $s_{2}^{n-2}$ to zero. Nonetheless, if it turns out, once again, that the coefficients of $s_{2}^{n-1}$ vanish on account of any possible value of κ, then one should proceed similarly and equate the coefficients of $s_{2}^{n-3}$ to zero, and so on, until one reaches the coefficient of $s_{2}^{0}$.

From Equation (A17), we now dedicate ourselves to the corresponding asymptotes of Equation (A18). For that, let us assume

(A28) $$s_{1}=\lambda s_{2}+\omega$$

to be the corresponding asymptote of Equation (A18), with the property that both λ and ω are real, where λ and ω denote the angular and linear coefficients of Equation (A28), respectively.

Proceeding in a manner analogous to that of Equation (A17), we are then led to conclude that the equations of the *m* oblique asymptotes related to Equation (A28) are given by

(A29) $$s_{1}=\omega_{i}\left[1+\left(\frac{\lambda_{i}}{\omega_{i}}\right)s_{2}\right]$$

or alternatively,

(A30) $$V_{i}\left(s_{1},s_{2}\right)=s_{1}-\omega_{i}\left[1+\left(\frac{\lambda_{i}}{\omega_{i}}\right)s_{2}\right]=0,$$

in which

(A31) $$\omega_{i}=-\left[\frac{\Psi_{m-1}\left(\lambda \right)}{\left(\frac{d\Psi_{m}\left(\lambda \right)}{ds_{2}}\right)}\right]\;\mathrm{at}\;\lambda =\lambda_{i},$$

where i=1,2,3,…,m.

Before continuing, let us summarize where we are now. From the computation of scalar curvature, we have imposed the condition that its denominator must vanish at the singularities of the magnitude of R. In connection with this latter point, we have written the denominator equation of R as a product of polynomial factors, in which both have been set equal to zero as a natural consequence of the previous condition. Finally, we have constructed the asymptotes of Equation (A13) or the corresponding asymptotes of Equations (A17) and (A18) upon employing the method of expansion of homogeneous polynomials.

With all this before us, the final step of our program is the construction of the singularities of |R| by determining the intersection points of the polynomial equations associated with each factor of the denominator of scalar curvature, Equations (A15) and (A16), with their correspondent asymptotes, Equations (A26) and (A29). For this purpose, it will be helpful to employ and extend the theory of resultants, which is an advantageous approach to solving problems with multivariate polynomial systems.

But before we embark on that outlined technique, however, we first briefly review some aspects of this mathematical formalism that find repeated application in the construction of the singularities of |R|. For this purpose, let

(A32) $$\phi \left(s_{1},s_{2}\right)=\sum_{i+j=0}^{n}{\tilde{u}}_{ij}s_{1}^{i}s_{2}^{j}$$

and

(A33) $$\psi \left(s_{1},s_{2}\right)=\sum_{i+j=0}^{m}{\tilde{v}}_{ij}s_{1}^{i}s_{2}^{j}$$

be two polynomials of degrees *n* and *m*, respectively, where ${\tilde{u}}_{00}{\tilde{v}}_{00}\neq 0$.

On recalling the hidden variable technique [113], we may rewrite the polynomials of ϕ and ψ as polynomials in just $s_{1}$ with coefficients that now depend on $s_{2}$. Precisely,

(A34) $$\phi \left(s_{1}\right)=\sum_{i+j=0}^{n}u_{ij}s_{1}^{i}$$

and

(A35) $$\;\psi \left(s_{1}\right)=\sum_{i+j=0}^{m}v_{ij}s_{1}^{i},$$

whence,

(A36) $$u_{ij}=\left({\tilde{u}}_{ij}s_{2}^{j}\right)\;\mathrm{and}\;v_{ij}=\left({\tilde{v}}_{ij}s_{2}^{j}\right).$$

Now, let us consider, $\alpha_{1},\alpha_{2},\ldots ,\alpha_{n}$ to be the roots of $\phi (s_{1})$. The two equations $\phi (s_{1})=0$ and $\psi (s_{1})=0$ have a root in common if and only if

(A37) $$\psi \left(\alpha_{1}\right)\psi \left(\alpha_{2}\right)\ldots \psi \left(\alpha_{n}\right)=0,$$

which denotes a symmetric function of degree *m* of the roots of the polynomial equation $\phi (s_{1})=0$. Consequently, we define

(A38) $$\mathrm{Res}\left(\phi ,\psi \right)=u_{00}^{n}\psi \left(\alpha_{1}\right)\psi \left(\alpha_{2}\right)\ldots \psi \left(\alpha_{n}\right)$$

to be the resultant of ϕ and ψ, which is a natural consequence of the determinant of a Sylvester matrix of order n+m.

After solving for the roots of Equation (A38), it is a simple matter to substitute those directly into $\phi (s_{1},s_{2})$ to find the roots relative to $s_{2}$. Bearing this in mind, we are then led to conclude that the number of zeroes of $\phi (s_{1},s_{2})$ is equal to the number of common points of $\phi (s_{1},s_{2})$ and $\psi (s_{1},s_{2})$, which is bounded above by the product of their degrees *n* and *m*, respectively, by virtue of Bézout’s theorem.

Having thus established contact with the well-developed theory of resultants, it is now our task to derive the singularities of the magnitude of R by determining the intersection points of the polynomial equations associated with each factor of the denominator equation for R and their corresponding asymptotes. We begin with the equation of the first polynomial factor, Equation (A15).

From Equation (A27), and assuming that Equation (A15) possesses a unique asymptote, for simplicity, we then have

(A39) $$\begin{array}{cc} M(s_{1},s_{2}) & =\sum_{i+j=0}^{n}{\tilde{q}}_{ij}s_{1}^{i}s_{2}^{j}=0,\\ \;N(s_{1},s_{2}) & =s_{1}-\sigma_{1}\left[1+\left(\frac{\kappa_{1}}{\sigma_{1}}\right)s_{2}\right]=0. \end{array}$$

On considering $M(s_{1},s_{2})$ and $N(s_{1},s_{2})$ as polynomials in just $s_{1}$ with coefficients that now depend on $s_{2}$, Equation (A39) now reduces to

(A40) $$\begin{array}{cc} M(s_{1}) & =\sum_{i+j=0}^{n}q_{ij}s_{1}^{i}=0,\\ N(s_{1}) & =s_{1}-\left(\sigma_{1}\left[1+\left(\frac{\kappa_{1}}{\sigma_{1}}\right)s_{2}\right]\right). \end{array}$$

Let $\varepsilon_{1},\varepsilon_{2},\ldots ,\varepsilon_{n}$ be the zeroes of $M(s_{1})$. With the aid of the theory of resultants, we find that the two equations $M(s_{1})=0$ and $N(s_{1})=0$ have a root in common if and only if

(A41) $$\mathrm{Res}(M\left(s_{1}\right),N\left(s_{1}\right))=0.$$

In a manner quite analogous to that presented for Equation (A39), we have, for the equation related to the second polynomial factor, Equation (A16),

(A42) $$\begin{array}{c} U\left(s_{1}\right)=\sum_{i+j=0}^{m}k_{ij}s_{1}^{i}=0,\\ V\left(s_{1}\right)=s_{1}-\omega_{1}\left[1+\left(\frac{\lambda_{1}}{\omega_{1}}\right)s_{2}\right]=0, \end{array}$$

in which, for simplicity, we have assumed that Equation (A16) has a unique asymptote.

Now, assuming $\varphi_{1},\varphi_{2},\ldots ,\varphi_{m}$ as the zeroes of $U(s_{1})$, we have, from the theory of resultants, that $U(s_{1})=0$ and $V(s_{1})=0$ have a root in common if and only if

(A43) $$\mathrm{Res}(U\left(s_{1}\right),V\left(s_{1}\right))=0.$$

From the knowledge of these resultant polynomial equations, the singular points of the magnitude of R are found by directly solving Equations (A41) and (A43) for $s_{1}$, and hence, for $s_{2}$. More specifically, we solve Equations (A41) and (A43) for $s_{1}$. Once the zeroes of the resultant equations relative to $s_{1}$ are found, it is be a simple matter to carry out the program previously mentioned to find the roots associated with $s_{2}$. Therefore, the zeroes of those systems of two polynomial equations, Equations (A39) and (A42), are the critical points for which the denominator of R becomes zero.

To conclude, the method of geometric characteristics supplies us with an approach to determine the maximum number of singularities of the magnitude of R. As we saw, the problem of finding the singularities of |R| is equivalent to determining the intersection points of Equation (A11) with its inclined asymptotes. Precisely, the number of zeroes or roots of the denominator of R, subject to the condition for which 1/R=0 at its singular points, is determined by the total number of intersection points between 1/R=0 and the implicit form of its vertical or oblique asymptotes by virtue of the well-known Bézout theorem and the theory of resultants.

We must emphasize that the procedures adopted above to construct asymptotes of algebraic curves and to determine the maximum number of solutions for systems of real polynomial equations by employing the Bézout theorem are not new ideas here. Precisely, these had already been considered some time ago by other authors and from somewhat different standpoints; see Refs. [83,84,85,87,120,125,126,127].

As a final remark, we wish to emphasize that the solutions of the polynomial equations corresponding to the angular and linear coefficients cannot be imaginary since only the real part of those coefficients is significant in the framework of GBT. Hence, for imaginary solutions of the angular and linear coefficients, we extract their real part in order to proceed with the construction of the singularities of the magnitude of scalar curvature.

With the method of geometric characteristics before us, we now illustrate its utility by applying it to the derivation of Equations (95) and (96) presented in the resolution of the two-dimensional version of the sixteenth problem of Hilbert. For this purpose, it is advisable to return to our starting point, that is, the Riemannian metric of second-degree polynomial systems, Equation (88):

$$\begin{array}{c} d\ell^{2}=2\left[{\left(a_{10}+s_{2}a_{11}+2s_{1}a_{20}\right)}^{2}+{\left(b_{10}+s_{2}b_{11}+2s_{1}b_{20}\right)}^{2}\right]{\left(ds_{1}\right)}^{2}\\ +2\left[{\left(a_{01}+2s_{2}a_{02}+s_{1}a_{11}\right)}^{2}+{\left(b_{01}+2s_{2}b_{02}+s_{1}b_{11}\right)}^{2}\right]{\left(ds_{2}\right)}^{2}. \end{array}$$

In deriving this result, we recall that Equation (88) induces a curvature R on the two-dimensional manifold of the parameter space of Equation (87). Precisely,

(A44) $$\begin{array}{cc} R & =[(\left(2{\left(2s_{1}a_{20}+s_{2}a_{11}+a_{10}\right)}^{2}+{\left(2s_{1}b_{20}+s_{2}b_{11}+b_{10}\right)}^{2}\right){\left(4a_{11}\left(s_{1}a_{11}+2s_{2}a_{02}+a_{01}\right)+2b_{11}\left(s_{1}b_{11}+2s_{2}b_{02}+b_{01}\right)\right)}^{2}\\ & +\left(2{\left(s_{1}a_{11}+2s_{2}a_{02}+a_{01}\right)}^{2}+{\left(s_{1}b_{11}+2s_{2}b_{02}+b_{01}\right)}^{2}\right)\left(8a_{20}\left(2s_{1}a_{20}+s_{2}a_{11}+a_{10}\right)+4b_{20}\left(2s_{1}b_{20}+s_{2}b_{11}+b_{10}\right)\right)\\ & \times \left(4a_{11}\left(s_{1}a_{11}+2s_{2}a_{02}+a_{01}\right)+2b_{11}\left(s_{1}b_{11}+2s_{2}b_{02}+b_{01}\right)\right)+\left(2{\left(s_{1}a_{11}+2s_{2}a_{02}+a_{01}\right)}^{2}+{\left(s_{1}b_{11}+2s_{2}b_{02}+b_{01}\right)}^{2}\right)\\ & \times {\left(4a_{11}\left(2s_{1}a_{20}+s_{2}a_{11}+a_{10}\right)+2b_{11}\left(2s_{1}b_{20}+s_{2}b_{11}+b_{10}\right)\right)}^{2}-8\left(2a_{11}^{2}+b_{11}^{2}\right)\\ & \times \left(2{\left(s_{1}a_{11}+2s_{2}a_{02}+a_{01}\right)}^{2}+{\left(s_{1}b_{11}+2s_{2}b_{02}+b_{01}\right)}^{2}\right)\left(2{\left(2s_{1}a_{20}+s_{2}a_{11}+a_{10}\right)}^{2}+{\left(2s_{1}b_{20}+s_{2}b_{11}+b_{10}\right)}^{2}\right)\\ & +\left(8a_{02}\left(s_{1}a_{11}+2s_{2}a_{02}+a_{01}\right)+4b_{02}\left(s_{1}b_{11}+2s_{2}b_{02}+b_{01}\right)\right)\left(4a_{11}\left(2s_{1}a_{20}+s_{2}a_{11}+a_{10}\right)+2b_{11}\left(2s_{1}b_{20}+s_{2}b_{11}+b_{10}\right)\right)\\ & \times \left(2{\left(2s_{1}a_{20}+s_{2}a_{11}+a_{10}\right)}^{2}+{\left(2s_{1}b_{20}+s_{2}b_{11}+b_{10}\right)}^{2}\right)){\left(-2\right)}^{-1}]F{\left(s_{1},s_{2}\right)}^{-2}, \end{array}$$

whence,

(A45) $$\begin{array}{cc} F(s_{1},s_{2}) & =H_{1}\left(s_{1},s_{2}\right)H_{2}\left(s_{1},s_{2}\right)\\ & =\left[\left(2{\left(s_{1}a_{11}+2s_{2}a_{02}+a_{01}\right)}^{2}+{\left(s_{1}b_{11}+2s_{2}b_{02}+b_{01}\right)}^{2}\right)\right]\\ & \times \left[\left(2{\left(2s_{1}a_{20}+s_{2}a_{11}+a_{10}\right)}^{2}+{\left(2s_{1}b_{20}+s_{2}b_{11}+b_{10}\right)}^{2}\right)\right]. \end{array}$$

From the computation of the scalar curvature, we now dedicate ourselves to the derivation of the singularities of the magnitude of Equation (A44) by following the standard procedure of the method of geometric characteristics. Therefore, we take the first step in this direction by imposing that $F(s_{1},s_{2})$ must be zero. That is to say,

(A46) $$F\left(s_{1},s_{2}\right)=H_{1}\left(s_{1},s_{2}\right)H_{2}\left(s_{1},s_{2}\right)=0,$$

from which it naturally follows that

(A47) $$H_{1}\left(s_{1},s_{2}\right)=\left[2{\left(s_{1}a_{11}+2s_{2}a_{02}+a_{01}\right)}^{2}+{\left(s_{1}b_{11}+2s_{2}b_{02}+b_{01}\right)}^{2}\right]=0,$$

and

(A48) $$H_{2}\left(s_{1},s_{2}\right)=\left[2{\left(2s_{1}a_{20}+s_{2}a_{11}+a_{10}\right)}^{2}+{\left(2s_{1}b_{20}+s_{2}b_{11}+b_{10}\right)}^{2}\right]=0.$$

As outlined previously, $H_{1}\left(s_{1},s_{2}\right)$ and $H_{2}\left(s_{1},s_{2}\right)$ are factors of the polynomial expression of the denominator of scalar curvature. By virtue of Equation (A46), and remembering that $H_{1}\left(s_{1},s_{2}\right)H_{2}\left(s_{1},s_{2}\right)=0$ means one or both $H_{1}\left(s_{1},s_{2}\right)$ and $H_{2}\left(s_{1},s_{2}\right)$ must be zero, this factorized form reveals that the solutions of Equation (A47) can be obtained by setting both factors $H_{1}\left(s_{1},s_{2}\right)$ and $H_{2}\left(s_{1},s_{2}\right)$ equal to zero. This latter operation completes the first step of our program.

Working forward from Equations (A47) and (A48), the second step relies on their correspondent asymptotes. Following closely the method of geometric characteristics, we begin by constructing the oblique asymptotes of Equation (A47).

On substituting $s_{1}=\kappa s_{2}+\sigma$ into Equation (A47), we arrive at

(A49) $$\begin{array}{cc} \; & \left[8a_{02}^{2}+8\kappa a_{02}a_{11}+2\kappa^{2}a_{11}^{2}+4b_{02}^{2}+4\kappa b_{02}b_{11}+\kappa^{2}b_{11}^{2}\right]s_{2}^{2}\\ + & \left[8a_{01}a_{02}+4\kappa a_{01}a_{11}+8\sigma a_{02}a_{11}+4\sigma \kappa a_{11}^{2}+4b_{01}b_{02}+2\kappa b_{01}b_{11}+4\sigma b_{02}b_{11}+2\sigma \kappa b_{11}^{2}\right]s_{2}\\ + & 2a_{01}^{2}+4\sigma a_{01}a_{11}+2\sigma^{2}a_{11}^{2}+b_{01}^{2}+2\sigma b_{01}b_{11}+\sigma^{2}b_{11}^{2}=0. \end{array}$$

Before proceeding, it should be noted that Equation (A49) becomes a polynomial equation of degree two written in terms of $s_{2}$. Therefore, to derive the angular and linear coefficients, we must equate the coefficients associated with $s_{2}^{2}$ and $s_{2}$ to zero, which yields

(A50) $$8a_{02}^{2}+8\kappa a_{02}a_{11}+2\kappa^{2}a_{11}^{2}+4b_{02}^{2}+4\kappa b_{02}b_{11}+\kappa^{2}b_{11}^{2}=0$$

and

(A51) $$8a_{01}a_{02}+4\kappa a_{01}a_{11}+8\sigma a_{02}a_{11}+4\sigma \kappa a_{11}^{2}+4b_{01}b_{02}+2\kappa b_{01}b_{11}+4\sigma b_{02}b_{11}+2\sigma \kappa b_{11}^{2}=0.$$

Solving Equation (A50) for κ, we find

(A52) $$\kappa_{1}=-\frac{2\left(2a_{02}a_{11}+b_{02}b_{11}+\sqrt{2}\sqrt{-{\left(a_{11}b_{02}-a_{02}b_{11}\right)}^{2}}\right)}{2a_{11}^{2}+b_{11}^{2}}$$

and

(A53) $$\kappa_{2}=-\frac{2\left(2a_{02}a_{11}+b_{02}b_{11}-\sqrt{2}\sqrt{-{\left(a_{11}b_{02}-a_{02}b_{11}\right)}^{2}}\right)}{2a_{11}^{2}+b_{11}^{2}}.$$

In accordance with the method of geometric characteristics, we have a corresponding value of σ for each value of κ. Therefore, the next step relies on the computation of the angular coefficients $\sigma_{i}$ with the aid of $\kappa_{i}$ derived above. Following closely the method of geometric characteristics, let us first derive $\sigma_{1}$. Solving Equation (A51) for σ and introducing $\kappa_{1}$ defined by Equation (A52), we arrive at

(A54) $$\begin{array}{c} \sigma_{1}=\frac{1}{\left(a_{11}b_{02}-a_{02}b_{11}\right)\left(2a_{11}^{2}+b_{11}^{2}\right)}[\left(a_{02}b_{01}b_{11}^{2}-a_{11}b_{01}\left(b_{02}b_{11}+\sqrt{2}\sqrt{-{\left(a_{11}b_{02}-a_{02}b_{11}\right)}^{2}}\right)\right.\\ \left.+a_{01}\left(-2a_{11}^{2}b_{02}+2a_{02}a_{11}b_{11}+\sqrt{2}b_{11}\sqrt{-{\left(a_{11}b_{02}-a_{02}b_{11}\right)}^{2}}\right)\right)]. \end{array}$$

On solving Equation (A51) for σ and introducing $\kappa_{2}$, Equation (A53), we find without further difficulty that

(A55) $$\begin{array}{c} \sigma_{2}=\frac{1}{\left(a_{11}b_{02}-a_{02}b_{11}\right)\left(2a_{11}^{2}+b_{11}^{2}\right)}[b_{01}\left(a_{02}b_{11}^{2}+a_{11}\left(-b_{02}b_{11}+\sqrt{2}\sqrt{-{\left(a_{11}b_{02}-a_{02}b_{11}\right)}^{2}}\right)\right)\\ -a_{01}\left(2a_{11}^{2}b_{02}-2a_{02}a_{11}b_{11}+b_{11}\sqrt{2}\sqrt{-{\left(a_{11}b_{02}-a_{02}b_{11}\right)}^{2}}\right)]. \end{array}$$

On extracting the real part in Equations (A52)–(A55), we obtain

(A56) $$\kappa_{1,2}=\kappa =\frac{-2(2a_{02}a_{11}+b_{02}b_{11})}{2a_{11}^{2}+b_{11}^{2}},$$

and

(A57) $$\sigma_{1,2}=\sigma =-\frac{2a_{01}a_{11}+b_{01}b_{11}}{2a_{11}^{2}+b_{11}^{2}}.$$

Thus, the first inclined asymptote of Equation (A46) is, from Equations (A56) and (A57),

(A58) $$s_{1}=-\left(\frac{2(2a_{02}a_{11}+b_{02}b_{11})}{2a_{11}^{2}+b_{11}^{2}}\right)s_{2}-\left(\frac{2a_{01}a_{11}+b_{01}b_{11}}{2a_{11}^{2}+b_{11}^{2}}\right).$$

An alternative representation of Equation (A58), and the one we shall employ for the calculations henceforth, is obtained by rewriting it implicitly as follows:

(A59) $$K\left(s_{1},s_{2}\right)=s_{1}+\left(\frac{2\left(2a_{02}a_{11}+b_{02}b_{11}\right)}{2a_{11}^{2}+b_{11}^{2}}\right)s_{2}+\left(\frac{2a_{01}a_{11}+b_{01}b_{11}}{2a_{11}^{2}+b_{11}^{2}}\right)=0,$$

which denotes an algebraic curve of degree one or a straight line. Proceeding in a manner entirely analogous to that used to obtain the first inclined asymptote, Equation (A59), from Equation (A47), we thereby encounter, from Equation (A48), another asymptote given by

(A60) $$L\left(s_{1},s_{2}\right)=s_{1}+\left(\frac{2a_{11}a_{20}+b_{11}b_{20}}{4a_{20}^{2}+2b_{20}^{2}}\right)+\left(\frac{2a_{10}a_{20}+b_{10}b_{20}}{4a_{20}^{2}+2b_{20}^{2}}\right)=0,$$

in which it is understood that the real part is to be taken.

Hence, we may conclude that Equation (A46) has two oblique asymptotes:

$$\begin{array}{cc} K(s_{1},s_{2}) & =s_{1}+\left(\frac{2\left(2a_{02}a_{11}+b_{02}b_{11}\right)}{2a_{11}^{2}+b_{11}^{2}}\right)s_{2}+\left(\frac{2a_{01}a_{11}+b_{01}b_{11}}{2a_{11}^{2}+b_{11}^{2}}\right)=0, \end{array}$$

and

$$\begin{array}{cc} L(s_{1},s_{2}) & =s_{1}+\left(\frac{2a_{11}a_{20}+b_{11}b_{20}}{4a_{20}^{2}+2b_{20}^{2}}\right)+\left(\frac{2a_{10}a_{20}+b_{10}b_{20}}{4a_{20}^{2}+2b_{20}^{2}}\right)=0. \end{array}$$

But, before continuing with the application of Equations (A59) and (A60) in the derivation of the singularities of the magnitude of scalar curvature, we must note that since Equation (A46) denotes a fourth-degree curve, one then would naturally expect not two, but rather four, different asymptotes.

Nevertheless, the results found above basically teach us that the denominator equation of R, Equation (A13), which constitutes an algebraic curve of degree *n*, can have, therefore, n/2 asymptotes as a natural corollary of the nature of the denominator equation of scalar curvature and the demand that the angular and linear coefficients of the asymptotes associated with it must be real. Somewhat surprisingly, such a conclusion is valid, as it is generally well-known that a plane algebraic curve of degree *n* can have no more than *n* asymptotes [83,84,85,87,120]. Bearing that in mind, we may then write that Equation (A46) possesses two different asymptotes, despite being a fourth-degree curve.

Armed with Equations (A59) and (A60), we may now proceed to the final stage of the method of geometric characteristics. Hence, we now construct the singularities of the magnitude of R with the aid of the theory of resultants. More specifically, we now exploit the intersection points of Equations (A47) and (A48) with their correspondent asymptotes.

Accordingly, let us first apply the mathematical formalism by examining the resultant of Equations (A47) and (A59). So, we start considering the system of equations relating $H_{1}\left(s_{1},s_{2}\right)$ and $K(s_{1},s_{2})$:

(A61) $$\begin{array}{cc} H_{1}\left(s_{1},s_{2}\right) & =\left[\left(2{\left(s_{1}a_{11}+2s_{2}a_{02}+a_{01}\right)}^{2}+{\left(s_{1}b_{11}+2s_{2}b_{02}+b_{01}\right)}^{2}\right)\right]=0,\\ K(s_{1},s_{2}) & =s_{1}+\left(\frac{2\left(2a_{02}a_{11}+b_{02}b_{11}\right)}{2a_{11}^{2}+b_{11}^{2}}\right)s_{2}+\left(\frac{2a_{01}a_{11}+b_{01}b_{11}}{2a_{11}^{2}+b_{11}^{2}}\right)=0. \end{array}$$

On employing $s_{2}$ as a hidden variable, the resultant of Equation (A61) relative to $s_{1}$ is simply

(A62) $$\begin{array}{cc} \mathrm{Res}(H_{1}\left(s_{1}\right),K\left(s_{1}\right)) & =\frac{1}{4{\left(2a_{20}^{2}+b_{20}^{2}\right)}^{2}}[4a_{11}^{2}a_{20}^{2}b_{10}^{2}-8a_{10}a_{11}a_{20}^{2}b_{10}b_{11}-16s_{1}a_{11}a_{20}^{3}b_{10}b_{11}+4a_{10}^{2}a_{20}^{2}b_{11}^{2}\\ & +16s_{1}a_{10}a_{20}^{3}b_{11}^{2}+16s_{1}^{2}a_{20}^{4}b_{11}^{2}+16s_{1}a_{11}^{2}a_{20}^{2}b_{10}b_{20}-16s_{1}a_{10}a_{11}a_{20}^{2}b_{11}b_{20}\\ & -32s_{1}^{2}a_{11}a_{20}^{3}b_{11}b_{20}+16s_{1}^{2}a_{11}^{2}a_{20}^{2}b_{20}^{2}+2a_{11}^{2}b_{10}^{2}b_{20}^{2}-4a_{10}a_{11}b_{10}b_{11}b_{20}^{2}\\ & -8s_{1}a_{11}a_{20}b_{10}b_{11}b_{20}^{2}+2a_{10}^{2}b_{11}^{2}b_{20}^{2}+8s_{1}a_{10}a_{20}b_{11}^{2}b_{20}^{2}+8s_{1}^{2}a_{20}^{2}b_{11}^{2}b_{20}^{2}\\ & +8s_{1}a_{11}^{2}b_{10}b_{20}^{3}-8s_{1}a_{10}a_{11}b_{11}b_{20}^{3}-16s_{1}^{2}a_{11}a_{20}b_{11}b_{20}^{3}+8s_{1}^{2}a_{11}^{2}b_{20}^{4}], \end{array}$$

whose roots are

(A63) $$s_{1}=-\frac{a_{10}b_{11}-a_{11}b_{10}}{2\left(a_{20}b_{11}-a_{11}b_{20}\right)}\;\mathrm{and}\;s_{1}=-\frac{-a_{11}b_{10}+a_{10}b_{11}}{2\left(a_{20}b_{11}-a_{11}b_{20}\right)}.$$

In other words, Equation (A62) is a quadratic polynomial expression with a double root at

(A64) $$s_{1}=-\frac{a_{10}b_{11}-a_{11}b_{10}}{2\left(a_{20}b_{11}-a_{11}b_{20}\right)}.$$

Upon substituting Equation (A64) into Equation (A61) and solving the resulting system of equations with respect to $s_{2}$, we find another double root at

(A65) $$s_{2}=\frac{-a_{20}b_{10}+a_{10}b_{20}}{a_{20}b_{11}-a_{11}b_{20}}.$$

Bearing this in mind, we may then write that the solutions of the system of equations relating $H_{1}\left(s_{1},s_{2}\right)$ and $K(s_{1},s_{2})$, Equation (A61), are

(A66) $$p_{1}^{C}=p_{2}^{C}=\left(-\frac{a_{10}b_{11}-a_{11}b_{10}}{2(a_{20}b_{11}-a_{11}b_{20})},\frac{-a_{20}b_{10}+a_{10}b_{20}}{a_{20}b_{11}-a_{11}b_{20}}\right).$$

With the aid of the theory of resultants, one may find, analogously to Equation (A61), that the solutions of the system of equations relating $H_{2}\left(s_{1},s_{2}\right)$ and $L(s_{1},s_{2})$,

(A67) $$\begin{array}{cc} H_{2}\left(s_{1},s_{2}\right) & =\left[\left(2{\left(2s_{1}a_{20}+s_{2}a_{11}+a_{10}\right)}^{2}+{\left(2s_{1}b_{20}+s_{2}b_{11}+b_{10}\right)}^{2}\right)\right]=0, \end{array}$$

(A68) $$\begin{array}{cc} L(s_{1},s_{2}) & =s_{1}+\left(\frac{2a_{11}a_{20}+b_{11}b_{20}}{4a_{20}^{2}+2b_{20}^{2}}\right)+\left(\frac{2a_{10}a_{20}+b_{10}b_{20}}{4a_{20}^{2}+2b_{20}^{2}}\right)=0, \end{array}$$

are simply

(A69) $$p_{3}^{C}=p_{4}^{C}=\left(-\frac{a_{10}b_{11}-a_{11}b_{10}}{2(a_{20}b_{11}-a_{11}b_{20})},\frac{a_{10}b_{20}-a_{20}b_{10}}{a_{20}b_{11}-a_{11}b_{20}}\right),$$

from which we conclude that the solutions of Equation (A46), or the zeroes of Equation (A45), are of multiplicity 2.

As a useful check on those results, we verify that the singularities derived above must satisfy Equation (A46). To this end, we evaluate Equation (A46) at Equations (A66) and (A69). Consequently, we find that

(A70) $$\begin{array}{cc} F(p_{1,2}^{C}) & =[2\left(2{\left(a_{01}+\frac{a_{11}\left(a_{01}b_{02}-a_{02}b_{01}\right)}{a_{02}b_{11}-a_{11}b_{02}}+\frac{a_{02}\left(a_{01}b_{11}-a_{11}b_{01}\right)}{a_{11}b_{02}-a_{02}b_{11}}\right)}^{2}\right.\\ & \left.+{\left(b_{01}+\frac{b_{11}\left(a_{01}b_{02}-a_{02}b_{01}\right)}{a_{02}b_{11}-a_{11}b_{02}}+\frac{b_{02}\left(a_{01}b_{11}-a_{11}b_{01}\right)}{a_{11}b_{02}-a_{02}b_{11}}\right)}^{2}\right)\\ & \times \left(2{\left(a_{10}+\frac{2a_{20}\left(a_{01}b_{02}-a_{02}b_{01}\right)}{a_{02}b_{11}-a_{11}b_{02}}+\frac{a_{11}\left(a_{01}b_{11}-a_{11}b_{01}\right)}{2(a_{11}b_{02}-a_{02}b_{11})}\right)}^{2}\right.\\ & \left.+{\left(b_{10}+\frac{2b_{20}\left(a_{01}b_{02}-a_{02}b_{01}\right)}{a_{02}b_{11}-a_{11}b_{02}}+\frac{b_{11}\left(a_{01}b_{11}-a_{11}b_{01}\right)}{2(a_{11}b_{02}-a_{02}b_{11})}\right)}^{2}\right)]=0, \end{array}$$

and

(A71) $$\begin{array}{cc} F(p_{3,4}^{C}) & =[2\left(2{\left(\frac{2a_{02}\left(a_{10}b_{20}-a_{20}b_{10}\right)}{a_{20}b_{11}-a_{11}b_{20}}-\frac{a_{11}\left(a_{10}b_{11}-a_{11}b_{10}\right)}{2(a_{20}b_{11}-a_{11}b_{20})}+a_{01}\right)}^{2}\right.\\ & \left.+{\left(\frac{2b_{02}\left(a_{10}b_{20}-a_{20}b_{10}\right)}{a_{20}b_{11}-a_{11}b_{20}}-\frac{b_{11}\left(a_{10}b_{11}-a_{11}b_{10}\right)}{2(a_{20}b_{11}-a_{11}b_{20})}+b_{01}\right)}^{2}\right)\\ & \times \left(2{\left(-\frac{a_{20}\left(a_{10}b_{11}-a_{11}b_{10}\right)}{a_{20}b_{11}-a_{11}b_{20}}+\frac{a_{11}\left(a_{10}b_{20}-a_{20}b_{10}\right)}{a_{20}b_{11}-a_{11}b_{20}}+a_{10}\right)}^{2}\right.\\ & \left.+{\left(-\frac{b_{20}\left(a_{10}b_{11}-a_{11}b_{10}\right)}{a_{20}b_{11}-a_{11}b_{20}}+\frac{b_{11}\left(a_{10}b_{20}-a_{20}b_{10}\right)}{a_{20}b_{11}-a_{11}b_{20}}+b_{10}\right)}^{2}\right)]=0. \end{array}$$

Therefore, we are led to conclude that Equations (A66) and (A69) are indeed the solutions of Equation (A46), which was the result we desired to demonstrate. Furthermore, the singular points derived above through the method of geometric characteristics teach us that the maximum number of four critical points leads to the divergence of the magnitude of Equation (A44) to infinity.
